# Adaptation of Organisms by Resonance of RNA Transcription with the Cellular Redox Cycle

**DOI:** 10.1371/journal.pone.0025270

**Published:** 2011-09-28

**Authors:** Viktor Stolc, Alena Shmygelska, Yuri Griko

**Affiliations:** 1 NASA Ames Research Center, Moffett Field, California, United States of America; 2 Carnegie Mellon University Silicon Valley, Moffett Field, California, United States of America; University of California Riverside, United States of America

## Abstract

Sequence variation in organisms differs across the genome and the majority of mutations are caused by oxidation, yet its origin is not fully understood. It has also been shown that the reduction-oxidation reaction cycle is the fundamental biochemical cycle that coordinates the timing of all biochemical processes in the cell, including energy production, DNA replication, and RNA transcription. We show that the temporal resonance of transcriptome biosynthesis with the oscillating binary state of the reduction-oxidation reaction cycle serves as a basis for non-random sequence variation at specific genome-wide coordinates that change faster than by accumulation of chance mutations. This work demonstrates evidence for a universal, persistent and iterative feedback mechanism between the environment and heredity, whereby acquired variation between cell divisions can outweigh inherited variation.

## Introduction

Time is an essential non-random variable of the information content and processing functions in cellular systems. Temporal coordination is fundamentally maintained by the oscillating reduction-oxidation reactions, i.e. the redox cycle. The redox cycle consists of successive forward and reverse transfers of electrons and protons in which reduced nicotinamide nucleotides drive the reduction of soluble low molecular weight thiols (e.g. glutathione), or disulfide bridges in proteins (e.g. thioredoxin and glutaredoxin) [1]. Molecular oxygen (O_2_) and hydrogen sulfide (H_2_S) act reversibly as diffusible allosteric gas effectors, affecting the redox cycle in opposite direction [1], [2]. The successive and reversible transfer of electrons and protons alternatively reverses functionally active or inactive states in many, if not most protein and RNA enzymes. While the activating functions of metabolism are easily recognizable, they are indivisible from the more subtle inactivating function of metabolic stasis. Both functions are reversibly maintained by the endogenous production of H_2_S, which inhibits the electron transport in most aerobic organisms by antagonism of the oxygen binding sites with sulfur. The reoxidation is catalyzed by endogenously generated reactive oxygen species (ROS) or by reaction with molecular oxygen (O_2_) in aerobic organisms [1].

Importantly, the redox cycle is universal and intrinsic among the three domains of life (archaea, bacteria, and eukaryotes), and despite its antiquity, it is mediated by highly conserved components that are genetically encoded and invariant to phylogenetic detection. The ubiquitous nature of the redox cycle presently extends to the Earth's redox state, which is an emergent property of microbial life on the planetary scale [3]. In cells, it is used for reversible activation or inactivation of proteins by altering their folding, structure, and function [4]–[6]. The cycle not only alternatively changes the functional activity of individual proteins by direct flipping of their “on" and “off" functional states, but also causes alternating global reprogramming of the “on/off" functional state of all proteins and RNAs within a given cell by a rhythmic RNA transcription mechanism [1]. The redox cycle appears to be generated endogenously, at least in part, by the rhythm of the transcriptome biosynthesis, which can oscillate multiple times between cell divisions [7]. Thus, many biological functions are possible from a single chemical function that oscillates in a binary state that is reduced or oxidized as the only possible values and operations on such values, remarkably synonymous with Boolean logic [8]. The ability to reversibly alternate between the “on" and “off" functional states enables gene products to compartmentalize essential reactions and even entire pathways in cellular metabolism temporally to avoid fundamental chemical conflict with each other.

The effect of a change in concentration, temperature, or other environmental conditions on the chemical equilibrium of the redox cycle, specifically its chemical products that are inherently reactive, unstable, and self-regenerating, shifts the equilibrium to counter-act the imposed change according to the Le Chatelier's Principle [9]. Importantly, environment-directed variation of heredity is a fundamental consequence of the Le Chatelier's principle because cellular functions, which are modulated by the redox cycle, including transcriptome biosynthesis, can be not only modulated in amplitude but also synchronized temporally by signals that originate in the external physical environment, particularly when such perturbations are punctuated in phase, or in resonance, with the endogenous redox cycle, which oscillates many times in a day [1]. For example, external perturbations of the redox cycle can modulate its amplitude and period (e.g. highly diffusible drugs, H_2_S, CO, and acetaldehyde) [1], [10]–[11].

A periodic increase in the oxidized cellular state by the environment may result in a universal responsive variation of the genetic code and therefore provide a substrate for speciation. This hypothesis is supported by the accumulation of oxidant-damaged nuclear DNA in aging cells and organisms [12], and the discontinuity of species originally detected as the absence of gradualism in the fossil record. The later observation led to the punctuated equilibrium theory, which attempts to explain static that dominates the history of most fossil records [13]. Additionally, the emergence and extinction of species has been correlated with the time of transitions in the geological cycles of carbon, sulfur, and oxygen [14]. Consistently, the present universal cellular metabolism responds equivalently to appropriate concentrations of O_2_ with activity, and by inactivity to H_2_S in aerobic organisms, or *vice-versa* in anaerobic organisms. The fundamental mechanism for the reversal of active and inactive metabolic states is encoded by the reversible direction of successive transfers of electrons and protons in the universal cellular metabolism [15].

Several experimental results suggest that mutation rate is non-uniform and varies across the genome by at least an order of magnitude in some organisms; however, such mutations appeared to be random [16]. Moreover, hypermutation under metabolic stress is increased significantly in microorganisms and in human cells [17], [18]. The unexpectedly high rate of mutation during nutritional stress may be caused by RNA transcription [19], which may be a major cause of transition mutations in nature [20].

We hypothesized that the temporal nature of the redox cycle and its chemical products, such as the endogenously generated ROS produced during the oxidative phase and thiols produced during the reductive phase, may not only balance the chemical equilibrium of the redox cycle, but also accumulate non-random DNA sequence variation, i.e. directed with respect to selective conditions of the environment, during RNA transcription. The mechanism is based on the asymmetric chemical reactivity in the transcribed DNA strands. Specifically, an exposure of the non-transcribed strand and coincident protection of the transcribed single stranded DNA∶RNA hybrid is caused by the transcription complex. The asymmetry results in differential reactivity of the two DNA strands to oxidation at actively transcribed loci. In contrast, DNA replication does not result in an asymmetric chemical reactivity of the two strands.

This effect would oscillate periodically and differentially for each gene, and thus non-randomly among genes, because the level of ROS varies during the redox cycle and decreases the reducing equivalents of NAD(P)H and H_2_S correspondingly to a minimum level in the oxidative phase when a subset of genes are transcribed [2]. Analysis of H_2_S production profiles during perturbation of the redox cycle previously revealed that the amount of H_2_S production is closely linked with cellular oxidative stress by periodic inhibition of respiratory activity, which correspondingly increases ROS periodically in the oxidative phase [21]. Moreover, the biochemical nature of the reductive and oxidative phases has been comprehensively analyzed and defined using gas and mass spectrometry of reduced and oxidized metabolites, which oscillate correspondingly in the appropriate phases of the redox cycle [22], [23].

Thus, the frequency of RNA transcription-induced mutation would accumulate in genes that are transcribed periodically, restricted to the time when the chemically reactive potential generated by the oxidative phase of the cycle is at its maximum. In contrast, genes transcribed exclusively during the reductive phase of the cycle would be less subject to chemically induced mutation, as was shown previously for the frequency of mutation from genome replication restricted to the reductive phase [7], [24]–[25]. As a result, the temporal resonance of RNA transcription with the oscillating binary state of the redox cycle would provide a fundamental and constant template for adaptation of the genetic code to the environment, “overwriting" any replication-based errors. Specifically, replication was previously shown to be gated by not one, but multiple redox cycles [7], during which H_2_S acts in a manner similar to cyanide by binding to the heme in cytochrome c oxidase and inhibits electron transport and ATP production, which leads to an increased ROS production [26]. This fundamental and universal mechanism of H_2_S-induced ROS production during the redox cycle applies to all aerobic cells. Thus, the maximum ROS production is induced persistently in each oxidative phase of the redox cycle as a response to the production of H_2_S in the preceding reductive phase. Consistently, the *cys4* mutant yeast strain that is a partial loss-of-function deficiency in H_2_S synthesis lacks a metabolic cycle, supporting the relationship of mutual induction between H_2_S and ROS [25].

The actual limit in the buffering capacity of the reducing equivalents to protect the genome from mutation by ROS is similar to the limited fidelity of other biochemical reactions, and it has been specifically demonstrated by decreasing the reductive power and increasing ROS in a yeast strain with a deletion of *TSA1* and/or *TSA2* genes that code the Thioredoxin peroxidases. The mutant strains fail to adequately neutralize ROS, which results in a mutator phenotype with as much as 2000% higher rate of mutation than the wild type strain [27]. Yeast cells that have the *TSA1* gene deleted are specifically sensitive to oxidative stress when the mitochondrial function is inhibited [28], as is persistently repeated in the redox cycle by H_2_S [21]. In general, blocking the electron transport chain by metabolites, such as H_2_S, results in ROS production [21]. Thus, a consequence of the redox cycle can be single-base-pair mutations caused by oxidative damage. Additionally, even large-scale genome instabilities can be the result of oxidative damage, and consistently, such large-scale genome changes can also result from a defect in the repair of abasic sites caused by oxidation, or as a consequence of imbalance in the nucleotide pool, rather than necessarily be independent events [29]–[34]. Remarkably, oxidation is an intrinsically periodic product of metabolism that is fundamental for adaptation of organisms because modulation of its amplitude beyond the buffering capacity of the reductive phase by resonance with the dynamically changing environment can result in changes of the genome sequence that can specify persistence *vs.* extinction.

Previously, circumstantial evidence for an environmentally responsive and simultaneously adaptive nature of mutation has been suggested [35]–[37] and actively debated [38]. A hierarchical, yet stochastic, and complex regulation of multiple cellular timing mechanisms had been suggested for adaptation without a heritable component [39]. In contrast, we present systematic evidence that the cellular redox cycle enables a simple, deterministic and heritable mechanism of phenotypic adaptation in response to an arbitrary challenge by the environment, consistently with the Le Chatelier's principle.

## Results

**Figure 1:**
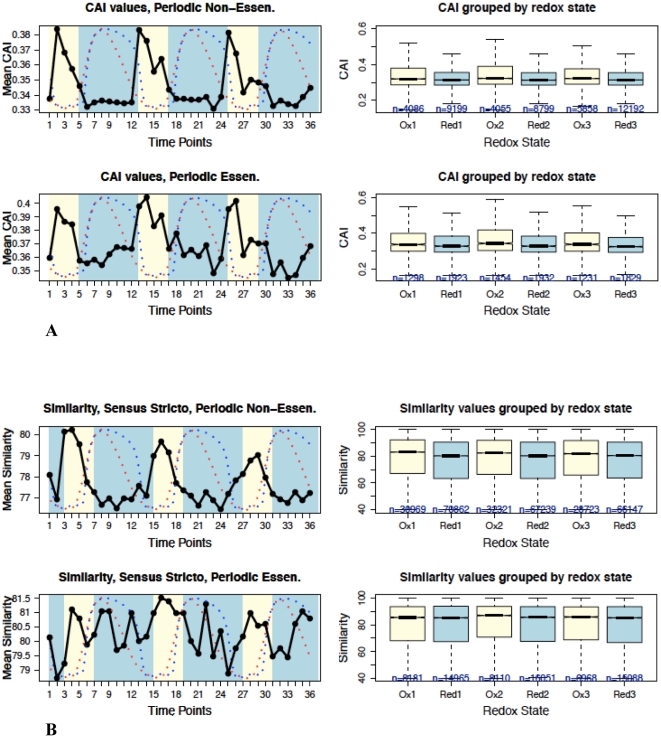
Yeast codons and phylogenetic conservation biased by the redox state. The relationship between dissolved oxygen (blue curve), hydrogen sulfide (red curve) (from reference 2), codon adaptation index (CAI, black line), phylogenetic protein-coding sequence similarity, the number of genes expressed during the redox cycle, and Spearman's correlation between CAI and log-normalized RNA expression during the redox cycle of yeast *S. cerevisiae*. Yellow color (time points 1–4) is the oxidative phase, and the blue color (time points 5–12) is the reductive phase. Boxes show median values with statistical significance with notches, (if the notches in the two boxes do not overlap, then it is a ‘strong evidence’ that their medians differ [60], first quantile (25%) and third quantile (75%); whiskers indicate minimum and maximum values. **A.** The oscillation of CAI values matching the period of the fluctuations in the oxidation state. **B.** The oscillation of yeast phylogenetic sequence similarity matching the period of the fluctuations in the oxidation state. Protein sequence conservation in *Saccharomyces sensu stricto* clade is graphed relative to the *S. cerevisiae* redox cycle.

**Figure 2:**
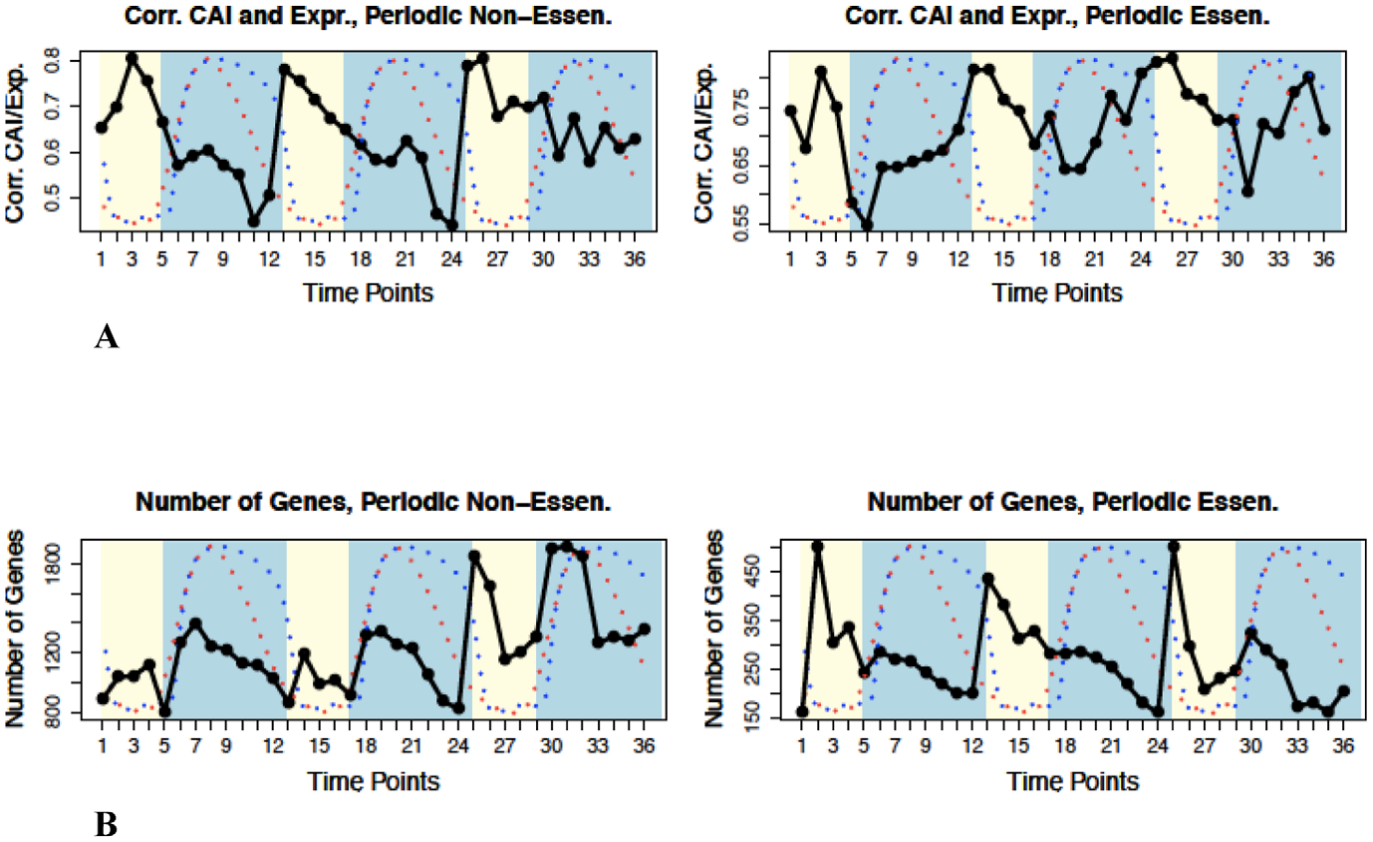
Transcript amplitudes and CAI correlate, and the numbers of expressed genes oscillate with the redox state. **A.** The oscillation of Spearman's correlation between CAI values and log-normalized RNA expression shows that a stronger correlation matches the oxidative phase. **B.** The oscillation in the number of genes expressed during the redox cycle. The number of essential genes (right panel) peak during the oxidative phase and the number of non-essential genes (left panel) peak during the reductive phase.

**Figure 3:**
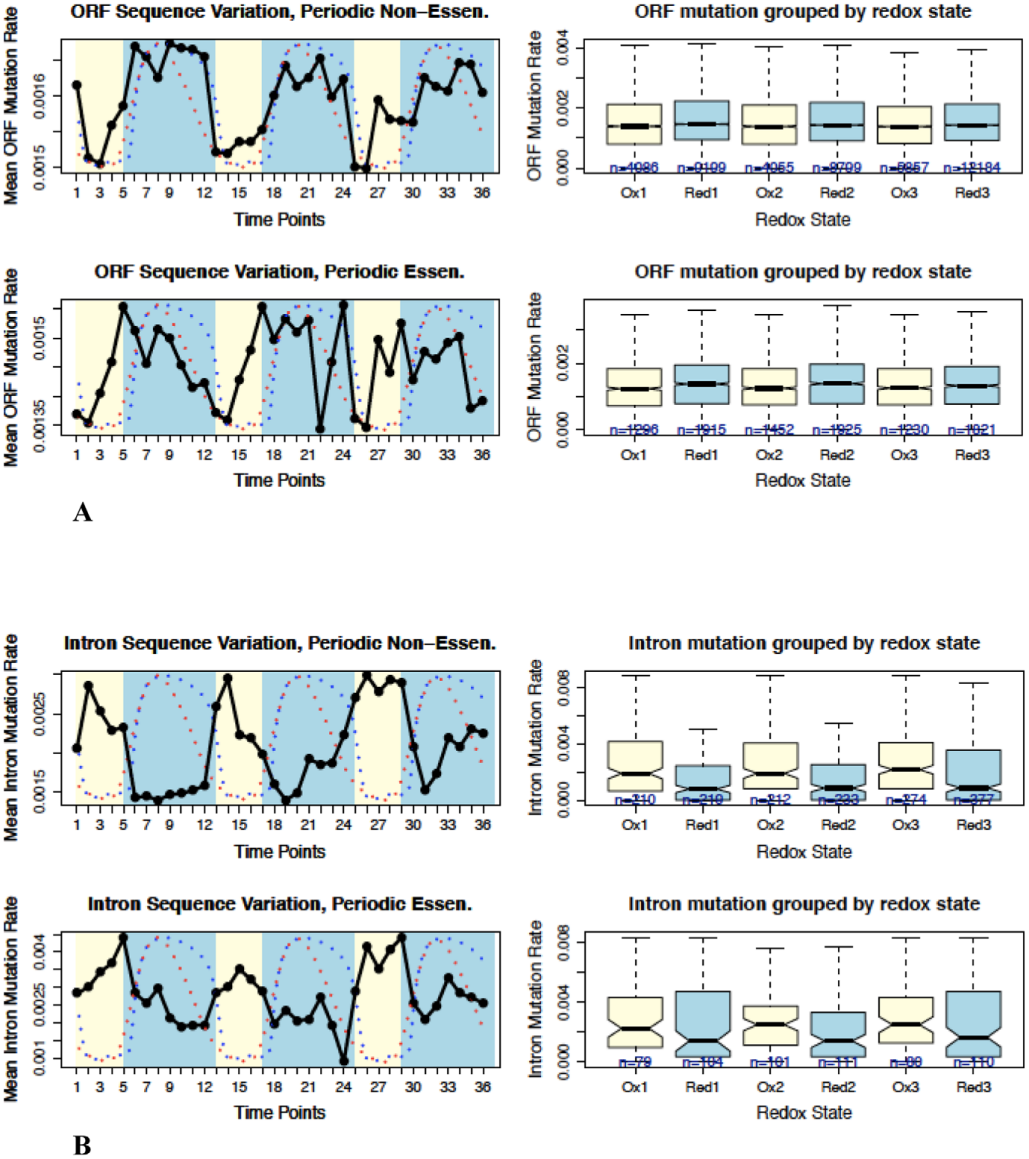
Genome-wide frequency of sequence variation biased differentially in gene structure elements by the redox state. The relationship between dissolved oxygen (blue dots), hydrogen sulfide (red dots) (from reference 2), and the frequency of sequence variation during the redox cycle of yeast *S. cerevisiae*. The opposite phased oscillations of the frequency of sequence variation values (black lines) in ORFs (**A**), and introns (**B**), match the period of the fluctuations in the redox states. The frequency of sequence variation is based on 101,343 distinct segregating sites of SNPs from 63 ecologically and geographically diverse types of laboratory-derived and wild type yeast strains.

To determine whether or not the universal feedback loop between H_2_S and ROS enables variation of the genome sequence and adaptation, the yeast genome and transcriptome were examined according to various self-consistent and coherent criteria correlated at discrete time intervals of the redox cycle to determine their phase of variation relative to the oxidized and reduced states. The chemically reactive potential generated by the oxidative phase of the redox cycle is at its maximum during the transition between high and low residual dissolved oxygen, and is at its minimum during the transition between low and high accumulated dissolved hydrogen sulfide [1], [2]. Specifically, we examined codon bias, i.e. codon adaptation index (CAI) [40] (**Fig. 1A**), variations of genome-wide RNA transcript levels, the number of genes (**Fig. 2A**, **Fig. 2B**, and **fig. S6**), and the frequency of sequence variation between regions of the *S. cerevisiae* genome transcribed in the oxidative *vs.* reductive phase of the redox cycle (**Fig. 3** **and fig. S4**). We also examined the degree of adaptation in the *S. cerevisiae* genome by analysis of phylogenetic sequence variation in all known yeast genes (**Fig. 1B** **. and fig. S3**).

### Resonance of the yeast transcriptome synthesis with the redox cycle

We analyzed yeast *S. cerevisiae* data from the transcriptional redox attractor cycle (TRAC) [10], and transcriptome data from similarly oscillating respiratory bursts during the yeast metabolic cycle (YMC) [24]. We identified individual open reading frames (ORFs) that were expressed periodically and aperiodically during each measured time interval in the redox cycle of both data sets (Supplemental Data S1). We treated the microarray data from the short (TRAC) and long-period (YMC) respiratory oscillations separately to identify agreement and disagreement between the datasets for each open reading frame (ORF) individually (Supplemental Data S1). Our method of analysis, described in Appendix S1, revealed a significantly greater degree of coherence between the two datasets than was previously reported. Our results resolve the issue about which genes peak at what phase of the cycle in favor of phase coherence in gene expression between the two datasets despite the difference in the length of the cycle periods.

Periodic ORFs oscillating with the redox cycle composed 3,614 out of 4,231 ORFs (∼85.4%) having consistent expression between the two data sets [10], [24], while 617 genes (∼14.6%) were non-periodic (see the Methods section for how the consistency of expression and periodicity was defined). The number of non-essential genes was 3,217 (∼76.0%) and number of essential genes was 1,014 (∼24.0%). Most non-essential genes are transcribed during the reductive phase, while most of the essential genes are transcribed in the oxidative phase (**Fig. 2B**), resulting in separate consideration.

### Resonance of the yeast codon bias with the redox cycle

ORFs transcribed at the time when the chemically reactive potential generated by the oxidative phase of the redox cycle is at its maximum, possess maximum bias (**Fig. 1A**). Conversely, the minimum bias is encoded in ORFs transcribed at the time when the chemically reactive potential generated by the oxidative phase is at its minimum. CAI values at synonymous sites of *S. cerevisiae* are on average ∼0.03 higher in the oxidative phase than in the reductive phase (*p*-value<2.2×10^−16^, one-sided Wilcoxon rank sum test). This corresponds to ∼9% increase in the mean CAI value for genes transcribed during the oxidative phase over the mean CAI value of genes transcribed during the reductive phase. The values given for the second cycle were chosen as representative. Similar oscillations were observed in all three cycles, **Table S1**. The increase in CAI values indicates that the two different frequencies of sequence variation at synonymous codons of genes transcribed in the oxidative and reductive phases are not equal, nor arbitrary.

The average CAI values for essential genes are ∼0.03 higher than for non-essential genes since the number of essential genes peaks at the oxidative phase (**Fig. 2B**), reflecting the difference in CAI values observed between the genes transcribed in the oxidative *vs.* reductive phase. Despite the differences in the pattern of gene number oscillation between the essential and non-essential genes, i.e. the number of essential genes peak during the oxidative phase and the number of non-essential genes peak during the reductive phase, the CAI value oscillation and oscillations of RNA expressions are the same for both groups.

Moreover, the CAI values are biased in genes expressed during the oxidative phase of the cycle, despite the fact that the relative expression levels of genes are similar or even higher in the reductive phase of the redox cycle [7]. This result shows that the level of transcription does not drive sequence variation independently of the redox phase. A higher rate of sequence variation may at first suggest that the CAI values would loose bias, because biased codons represent only a single option out of several codon redundancy options that are likely produced by the variation of sequence through the repair of oxidized bases. In reality, the persistent variation of sequence by repair of oxidized bases maintains codon bias when combined with selection. For example, a comparison of CAI values for genes encoding the *S. cerevisiae* cytoplasmic and mitochondrial ribosomes shows that CAI values are biased by the oxidative phase of the redox cycle, and not by some other independent factor. Specifically, genes encoding the cytoplasmic ribosome subunits are co-transcribed in the oxidative phase and have expression levels similar to genes that encode the mitochondrial ribosome, which, in contrast, is encoded by genes that are co-transcribed in the reductive phase of the redox cycle [24]. Yet, despite the similar levels of gene expression between the two different types of ribosomes-coding genes, the cytoplasmic ribosome has more than double the average CAI value of the mitochondrial ribosome, i.e. 0.761 *vs.* 0.297. This result clearly demonstrates that CAI values are biased by the oxidative phase, and not by transcription level independent of the redox phase in which the corresponding genes are transcribed. Consistently with the CAI bias, the average amino acid sequence similarity between the yeast and human ribosomes is 60% in the cytoplamsic ribosome *vs.* 33% in the mitochondrial ribosome. The least conserved yeast cytoplasmic ribosome protein, Rpp1B, has 37% sequence similarity with its human orthologue, and the most conserved yeast cytoplasmic ribosome protein, Rpl40B, has 90% sequence similarity with its human orthologue. In contrast, the least conserved yeast mitochondrial ribosome protein, Mrpl3, has 20% sequence similarity with its human orthologue, and the most conserved yeast protein, Mrps12, has 55% sequence similarity.

Remarkably, the correlation between the amplitude of genome-wide RNA transcripts and CAI values also oscillates with the redox cycle (Spearman's rank method, *p*-value<10^−10^ for all 36 time points for both essential and non-essential genes, **Fig. 2A**). The correlation is stronger during the oxidative phase and relaxes during the reductive phase, suggesting that CAI values during the oxidative phase are more representative of gene transcription levels, consistent with a higher RNA turnover in the oxidative environment. **Figure S9** shows the result of modeling the relationship between CAI values and the total log-normalized RNA expression level in the oxidative phase for each gene in *S. cerevisiae*.

Additionally, because RNA turnover is higher in the oxidative *vs.* reductive phase of the redox cycle, it was verified that the rate of RNA synthesis alone does not have a role in differentiating CAI values for the two types of ribosomes-coding genes independently of the redox phases. The rates of yeast mRNA synthesis, determined previously [41], were compared for all the ribosomal genes to show that the turnover of the mRNAs that code for the cytoplasmic and mitochondrial ribosomes are the same despite the fact that the induced rate of mRNA synthesis on average is two times higher in the oxidative phase for the cytoplasmic ribosome-coding ORFs compared to the reductive phase for the mitochondrial ribosome-coding ORFs. However, this result is due to an equivalent difference in the length of the oxidative *vs.* reductive phase during which the respective genes are transcribed. Specifically, the duration of the oxidative phase is half of the reductive phase, and this difference also accounts for the same maximum amplitude of RNA transcripts for the two different ribosomes, which are expressed exclusively in the opposite phases of the redox cycle. Consistent with the product of duration and rate of synthesis for both types of ribosomes, mRNA transcripts of the cytoplasmic ribsosome accumulate as short duration sharp burst peaks exclusively during the oxidative phase, whereas all mRNA transcripts for the mitochondrial ribosome accumulate as broad plateau peaks exclusively during the reductive phase.

To further investigate the differences in CAI values between ORFs transcribed in the oxidative *vs.* reductive phases, we examined the base identity at the third positions within synonymous codons for all ORFs. The identity of the third bases in codons of all periodically expressed ORFs oscillates with the redox cycle, and it is biased against guanine (G) and adenine (A), i.e. purine bases, and for thymine (T) and cytosine (C), i.e. pyrimidine bases, by ∼1% with all *p*-values<10^−3^ (**fig. S8**). A higher depurination in the oxidative phase is consistent with an oxidative damage to the DNA [42]. Moreover, repair of oxidative DNA damage *in vivo* results in transversions, which change the chemical nature of the base, thus enabling maximum mutagenic potential and the most rapid genetic variation possible. Interestingly, despite adenine being most efficiently inserted when abasic sites are bypassed by DNA polymerases, it is clear that the fidelity of translation is selected over the enzymatic specificity for purines.

Additionally, we examined CAI values in *Saccharomyces sensu stricto* clade (*S. paradoxus, S. mikatae, S. kudriavzevii, S. bayanus, S. castellii, S. kluyvery*) genes and in human protein-coding genes that are similar in their amino acid sequence coding potential to *S. cerevisiae* ORFs (Blast *e*-value<10^−50^) (Supporting online material, **fig. S1, S2, Table S1**). The results show a periodic oscillation of CAI values for orthologous ORFs in other species of yeast as well as in human ORFs.

The frequency of codon bias is enumerated between two classes of transcribed regions defined non-randomly by temporal segregation between incompatible functions of genes in the redox cycle within an individual organism, as well as, consistently, in different species due to the universal nature of the redox cycle in living cells. Thus, the CAI bias is an intrinsic consequence of iteration between heredity and the environment for all cells, and it is consistent with the observation that the redox cycle is independent of either population dynamics such as synchronization among cells or external limitation of growth by the carbon source [43].

Also, while the tRNA pool contains differential amounts of isoacceptors, i.e. tRNAs with different anticodons but incorporating the same amino acid in protein synthesis, each tRNA isoacceptor amount matches its condon bias, as was shown for yeast and *E. coli* [44], [45]. Therefore, the oscillating CAI bias among all ORFs is a result of the relative timing of expression of mRNAs in the redox cycle, rather than biased by different amounts of the tRNA isoacceptors, which accumulate proportionally to the CAI bias.

### Resonance of the yeast phylogenetic conservation with the redox cycle

A pair wise sequence similarity comparison between *S. cerevisiae* ORFs and all ORFs in the *Saccharomyces sensu stricto* clade matching the period of the redox cycle revealed that, remarkably, the sequence similarity also oscillates with the redox cycle. This result indicates that genes of other yeast species are likely similarly organized in their expression relative to the *S. cerevisiae* redox phases because variation of homologous sequences is maximal for genes expressed in the oxidative phase, when ROS reaches maximum value [21]. The cycle is slightly shifted; presumably due to variation in the time of expression for orthologous ORFs in *S. cerevisiae* relative to the other yeast species (**Fig. 1B** and **Table S2**). The sequence similarity on average is ∼1.0% higher in the oxidative phase compared to the reductive phase (*p* -value<2.2×10^−16^, one-sided Wilcoxon rank sum test), and is ∼3.0% higher in essential genes *vs.* non-essential genes. The conserved sequences are an indication of functional importance, and are expected to be under a stronger purifying and/or fixing selection in non-synonymous sites during the oxidative *vs.* the reductive phase. A similar trend holds for a pair wise sequence similarity of human genes that are similar in amino acid sequence to *S. cerevisiae* non-essential genes (∼1% higher in oxidative phase, *p*-value<10^−4^, one sided Wilcoxon rank sum test), however, for human genes that are similar in amino acid sequence to *S. cerevisiae* essential ORFs, there is no significant difference in variation of sequence conservation between oxidative and reductive phases (**fig. S2** and **Table S2**).

These results may seem counterintuitive because a higher rate of sequence variation would imply a lower potential for sequence conservation, but this would only be true in the absence of selection. In reality, amino acid sequence conservation and CAI bias both result from a high rate of sequence variation that is restrained by selection of orthologous biological function.

Should chance alone be the mutation frequency determinant, there would be no basis for periodic oscillation of the phylogenetic sequence similarities, or oscillation and bias of CAI values in phase with the *S. cerevisiae* redox cycle. These results indicate that the fidelity of translation and biological functions are both optimized by genetic variation induced through RNA transcription in addition to DNA replication rather than through variation of the genetic code by the imperfect fidelity of DNA replication alone. Interestingly, the CAI bias is also present in non-essential ORFs, clearly indicating that their functions are non-trivial and actively optimized by selection, as previously detected in a phenotypic assay that revealed a selection pressure on non-essential ORFs [46].

### Non-random, RNA transcription-induced frequency of sequence variation in the yeast genome

We examined the difference in the experimentally determined frequency of sequence variation between genes transcribed in the oxidative and reductive phases of the cycle and estimated the frequency of sequence variation induced by RNA transcription in yeast directly (**Table 1**). This was done by comparing the frequency of sequence variation among ORFs, introns, and un-translated regions (UTRs), i.e. in both 5′UTRs and 3′UTRs [47] transcribed in the oxidative and reductive phases of the redox cycle. In addition, we compared the frequency of sequence variation in transcribed DNA (ORFs, introns, 5′ and 3′UTRs) with the estimated frequency of sequence variation in un-transcribed regions [48]. We used previously mapped whole-genome single nucleotide polymorphisms (SNPs) in non-repetitive regions that were determined using tiling microarrays for 63 ecologically and geographically diverse *S. cerevisiae* strains [49]. The most comprehensively validated SNP dataset that contains 101,343 distinct segregating sites mapped in wild type yeast genotypes was used because the yeast metabolic cycle is an intrinsic property of yeast metabolism and does not depend on either synchronization or external limitation of growth by the carbon source [43].

**Table 1:** Table of the mean frequencies of sequence variation (in ORFs, introns, 5′UTRs and 3′UTRs) per nucleotide for the oxidative and reductive phases.

		ORF			Intron			5′UTR			3′UTR	
**Non-essential genes**	Ox		Red	Ox		Red	Ox		Red	Ox		Red
	(1–4)		(5–12)	(1–4)		(5–12)	(1–4)		(5–12)	(1–4)		(5–12)
	1.5×10^−3^±		1.6×10^−3^±	2.5×10^−3^±		1.8×10^−3^±	4.9×10^−3^±		4.2×10^−3^±	2.8×10^−3^±		3.0×10^−3^±
	1.0×10^−3^		1.0×10^−3^	2.3×10^−3^		2.1×10^−3^	6.6×10^−3^		5.6×10^−3^	3.2×10^−3^		3.5×10^−3^
	(SD, N = 4055)		(SD, N = 8799)	(SD, N = 212)		(SD, N = 233)	(SD, N = 1898)		(SD, N = 3988)	(SD, N = 2366)		(SD, N = 5285)
		*p-value* = **2.2×10^−6^**			*p-value* = **3.8×10^−6^**			*p-value* = **1.5×10^−3^**			*p-value* = 0.17	
		ratio = **0.95**			ratio = **1.43**			ratio = **1.15**			ratio = **0.94**	
		(C.I. 95%, 0.92–0.97)			(C.I. 95%, 1.18–1.73)			(C.I. 95%, 1.07–1.24)			(C.I. 95%, 0.89–1.00)	
**Essential genes**												
	1.4×10^−3^±		1.5×10^−3^±	3.2×10^−3^±		2.3×10^−3^±	5.0×10^−3^±		4.3×10^−3^±	3.3×10^−3^±		3.8×10^−3^±
	1.0×10^−3^		1.0×10^−3^	3.0×10^−3^		2.6×10^−3^	7.6×10^−3^		6.2×10^−3^	5.2×10^−3^		7.3×10^−3^
	(SD, N = 1452)		(SD, N = 1925)	(SD, N = 101)		(SD, N = 111)	(SD, N = 648)		(SD, N = 866)	(SD, N = 841)		(SD, N = 1127)
		*p-value* = **0.0036**			*p-value* = **0.0037**			*p-value* = 0.91			*p-value* = **0.011**	
		ratio = **0.94**			ratio = **1.40**			ratio = **1.15**			ratio = **0.86**	
		(C.I. 95%, 0.89–0.98)			(C.I. 95%, 1.06–1.86)			(C.I. 95%, 1.00–1.34)			(C.I. 95%, 0.74–1.01)	

Standard deviations (SD), *p*- values for the two-tailed Wilcoxon rank sum test, and frequency of sequence variation ratios between the oxidative and reductive phases, and their 95% confidence intervals (C.I. 95%) for *S. cerevisiae* over the time of divergence for 63 strains. Ox (1–4) designates the four time points of the oxidative phase, and Red (5–12) designates the eight time points of the reductive phase in the redox cycle.

**Table 1** is a summary for the frequency of sequence variation per nucleotide of genes expressed periodically at the oxidative or the reductive phases of the *S. cerevisiae* redox cycle over the unknown period of time required for divergence of the 63 yeast strains. The observed frequencies of sequence variation within ORFs, introns, 5′UTRs and 3′UTRs persistently oscillate in phase with the redox cycle and are the result of appropriate interplay between sequence variation and selection, where the effect of selection is believed to be the strongest in ORFs, and nearly neutral in introns, 5′UTRs and 3′UTRs. Consistently, in the protein-coding portions of genes where selection is expected to play the biggest role for adaptation, the frequency of sequence variation has the lowest value, indicating a limited potential for productive mutation (**Fig. 3A** and **Table S3**). While synonymous codons (i.e. CAI values) can be positively biased, the negative bias against non-synonymous codons reveals a fundamental restriction in the potential for variation of amino acid identity. This result indicates a non-trivial limitation for gradualism as a mechanism of adaptation to the dynamic environment by speciation.

The frequency of sequence variation is anti-correlated with CAI values in ORFs and 3′UTRs, and positively correlated in introns and 5′UTRs, consistent with sequence variation being mostly deleterious to the function of coding sequences [50]–[52] (**Fig. 3** and **fig. S4**). The frequency of sequence variation in 3′UTRs, which encode structured RNA-recognition sites for protein binding, *e.g.* mitochondrial targeting sequences [53], is anti-correlated with the oxidative phase, indicating a negative selection against variation, while the 5′UTRs do not show this trend. Consistent with this difference, 3′UTRs overlap adjacent ORF-coding regions, which are anti-correlated with the sequence variation frequency, while the 5′UTRs do not show this trend. In addition, a diminishing gradient in the frequency of sequence variation along the gene lengths is consistent with a reduction in processivity of RNA transcription elongation. Unfailingly, the occupancy of RNA Polymerase II along gene lengths has been mapped on a genome-wide scale in yeast and shown to decrease from the transcription start sites at the 5′ end toward the 3′ end of genes. An apparent increase in occupancy of RNA Polymerase II at the polyadenylation sites near the 3′ end of genes was also consistently mapped due to the overlap of 3′UTRs with adjacent ORF-coding regions [41]. Thus, the failure of the transcription complex to translocate successfully to the 3′ end results in a higher frequency of sequence variation in the 5′ UTRs than in the 3′ UTRs, as detected (**Table 1** **, Table S3**, and **fig. S4**).

The analysis of the difference in the frequency of sequence variation between oxidative and reductive states among 158 out of 308 known *S. cerevisiae* intron-containing genes [47], which are presumed to be neutral to selection in their intron-coding sequences [54], revealed that intron sequences transcribed during the oxidative phase contained 39.0% higher mean frequency of sequence variation per nucleotide over the 63 strain divergence time than introns transcribed during the reductive phase of the cycle. The known occurrence of yeast intron sequences that can be affected by selection is limited to a few bases in comparison with the total length of yeast introns, i.e. 66,180 bases in ORF-containing introns, and therefore does not affect the general assumption that intron sequences are neutral to the effect of selection. For example, the intron encoded polypyrimidine tract and a single adenosine in the branch point of introns, in addition to a single small RNA (snR191) within the intron of an ORF called YNR053C, contribute less than a few percent of selection-affected bases to the total number of considered bases. In comparison to introns, the difference between the frequency of sequence variation in 5′UTRs of genes transcribed in the oxidative *vs.* the reductive phase was 15.2%. The lower frequency of sequence variation in 5′UTRs *vs.* introns is consistent with a higher content of selectable sequence encoded within 5′UTRs by upstream ORFs, i.e. uORFs [55], [56]. Because uORFs encode amino acid identity, the effect of selection is against their variation. Thus, the frequency of sequence variation is higher in the oxidative *vs.* the reductive phase in nearly neutral sites, confirming RNA transcription as a templating mechanism that increases the mutation potential among genes transcribed in the oxidative *vs.* reductive phase of the redox cycle.

Additionally, both the number and lengths of the selection-neutral genetic elements and ORFs oscillate during the redox cycle, matching the period of the fluctuations in the oxidation state (**fig. S5 and fig. S7**). This result is consistent with propagation of the length and number of genetic elements by the repair of their oxidized bases, which can lead to base extensions at the damaged sites.

Finally, we estimated the difference in the frequencies of sequence variation between transcribed DNA (ORFs, introns, 5′ and 3′UTRs) and un-transcribed DNA. The coordinates of the un-transcribed regions were identified by incorporating data from 36-mer oligonucleotides mapped to regions with nearly absent expression levels [48] and checking for overlap with non-coding regions [50], absence of coding elements [47], and absence of 5′ and 3′UTRs [56]. In total, out of nearly 400,000 unique 36-mers tiled across the genome, 584 specific 36-mers were identified as potential un-transcribed regions, and an upper bound frequency of sequence variation of 2.50×10^−3^±5.18×10^−3^(SD, N = 584) per nucleotide was derived for the un-transcribed DNA over the divergence time of the 63 strains. Based on this estimate, the ratio of the frequencies of sequence variation between transcribed introns, i.e. selection nearly neutral DNA, and un-transcribed DNA is at least 1.26(C.I. 95%, 0.98±1.62, N = 101) for essential genes transcribed during the oxidative phase *vs.* 0.90(C.I. 95%, 0.69±1.18, N = 111) for essential genes transcribed in the reductive phase. The same ratio for the frequency of sequence variation in introns of non-essential genes is at least 1.01(C.I. 95%, 0.82±1.25, N = 212) for genes transcribed in the oxidative phase *vs.* 0.71(C.I. 95%, 0.56±0.89, N = 233) for genes transcribed in the reductive phase. Thus, the estimated ratios indicate that RNA transcription-induced variation of sequence significantly increases the total rate of mutation.

## Discussion

In contrast to the common presumption that the passage of time alone serves as a template for random variation of heredity, we showed that the variation of genetic sequence is specified persistently and non-randomly by the temporal resonance of RNA transcription with the binary chemical state of the redox cycle. The rate of genetic variation by RNA transcription persistently increases the total rate of mutation and oscillates asymmetrically with the redox cycle among genes. Moreover, the measured RNA transcription-induced frequency of sequence variation (**Table 1**) is an underestimation because (1) determining the frequency of sequence variation in un-transcribed regions is challenging due to their scarcity in *S. cerevisiae* genome, and (2) grouping of individual time points in the two phases of the redox cycle introduces dilution of the relative values at the points of chemical extrema. Additionally, negative selection reduces the observable frequencies of sequence variation at the two phases of the redox cycle. Importantly, a random-based asymmetry in the rate of sequence variation would result in a constant variation among genome-wide transcribed regions relative to the period of the redox cycle, rather than oscillate in phase with the timing of the two phases of the redox cycle. Because time is an essential non-random variable of the information content and processing functions in cellular systems, the redox cycle clearly provides a temporal template for asymmetry in the genome-wide rate of sequence variation.

A biochemical mechanism that can account for the increased total frequency of transcription-induced sequence variation over the replication-based mutation is the asymmetric chemical reactivity between the transcribed and replicated DNA strands. Specifically, the confinement of a single round of replication to the reductive phase of the cycle is protective against variation of sequence by base oxidation [7], [24], while multiple rounds of genome-wide RNA transcription between cell divisions oscillate in the chemical environment of the reactive and unstable products of the oxidizing phase, which results in non-uniform and varied frequency of mutation across the genome. Additionally, it is important to recognize that the periodic oscillation in the frequency of sequence variation for all relevant genetic elements is appropriately in agreement with the central dogma of molecular biology. In contrast to the reality of our results, a random process as previously contemplated by others could only generate aperiodic or irregular values for the same variables of heredity. Moreover, the paradox of biological adaptation by means of such supposedly passive and random events is *a priory* inconsistent, because it is incorrect to assume that a random process can generate asymmetry without a template, especially for a periodic asymmetry of the redox cycle. Perhaps because the environment is dynamic, it can be non-obvious that the environment varies periodically on a number of different time-scales and its impact is matched by responsively dynamic changes in cellular functions according to the Le Chatelier's principle. The temporally ordered biochemical events and asymmetry of the redox cycle chemistry on DNA *vs.* RNA provide the non-random basis for a dynamic heredity in response to the environment, consistently with the Le Chatelier's principle.

The concept of adaptation through use and disuse of characteristics, whereby acquired variation outweighs inherited variation was admitted by Darwin [57]. However, Gregor Mendel's subsequent definition of static genetic inheritance [58] supplanted the dynamic notion of inheritance of acquired traits [59]. Our results may fundamentally change the common understanding about the underlying mechanism at work in adaptation and persistence of organisms. While the basis of the observed genetic and phenotypic discontinuity is broadly attributed to a continuum of evolution by passive accumulation of chance mutation, i.e. random alternations followed by selection and determination of a choice, we present evidence for acquired genetic variation that is actively induced by the environment between generations. The discrete and iterative feedback between the environment and heredity is mediated by the redox cycle, which may serve as a template mechanism for spontaneously acquired adaptation and speciation of organisms toward persistence in a dynamically changing environment.

## Materials and Methods

Computational protocols and statistical methods of analysis used for evaluating microarray data, calculations of periodicity, codon usage bias, amino acid sequence similarity, and the frequency of sequence variation are described in the Supporting materials index.

## Supporting Information

Figure S1 **The relationship between dissolved oxygen (blue curve), hydrogen sulfide (red curve) (from reference 1), codon adaptation index (CAI, black line) of** ***sensu stricto*** **clade genes (** ***S. paradoxus, S. mikatae, S. kudriavzevii, S. bayanus, S. castellii, S. kluyvery*** **) similar in amino acid sequences to** ***S. cerevisiae*** **ORFs expressed periodically during the redox cycle.** Yellow color (time points 1–4) is the oxidative phase, and the blue color (time points 5–12) is the reductive phase. Boxes show median values with statistical significance with notches (if two boxes' notches do not overlap this is a ‘strong evidence’ that their medians differ [2]), first quantile (25%) and third quantile (75%); whiskers indicate minimum and maximum values.
(DOC)

Figure S2 **The relationship between codon adaptation index (CAI, black line) for** ***H. sapiens*** **genes that are similar in amino acid sequence to** ***S. cerevisiae*** **genes expressed periodically in the redox cycle.** Yellow color (time points 3–6) is the suggested oxidative phase, and the blue color (time points 1–2 and 7–12) is the suggested reductive phase. Boxes show median values with statistical significance with notches (if two boxes' notches do not overlap this is a ‘strong evidence’ that their medians differ [2]), first quantile (25%) and third quantile (75%); whiskers indicate minimum and maximum values.
(DOC)

Figure S3 **The relationship between sequence similarity of** ***H. sapiens*** **genes to** ***S. cerevisiae*** **genes periodically expressed in the redox cycle.** Yellow color (time points 3–6) is the suggested oxidative phase, and the blue color (time points 1–2 and 7–12) is the suggested reductive phase. Boxes show median values with statistical significance with notches (if two boxes' notches do not overlap this is a ‘strong evidence’ that their medians differ [2]), first quantile (25%) and third quantile (75%); whiskers indicate minimum and maximum values.
(DOC)

Figure S4 **The relationship between dissolved oxygen (blue dots), hydrogen sulfide (red dots) (from reference 1), and the frequency of sequence variation during the redox cycle of yeast** ***S. cerevisiae*** **.** The opposite phased oscillations for the frequency of sequence variation values in 5′UTRs and 3′UTRs (black lines) match the period of the fluctuations in the redox states.
(DOC)

Figure S5 **A. The relationship between number and length of introns, 5′ and 3′UTRs in** ***S. cerevisiae*** **genes and the redox cycle.** Yellow color (time points 1–4) is the oxidative phase, and the blue color (time points 5–12) is the reductive phase. Boxes show median values with statistical significance with notches (if two boxes' notches do not overlap this is a ‘strong evidence’ that their medians differ [2]), first quantile (25%) and third quantile (75%); whiskers indicate minimum and maximum values. **A.** The oscillation of intron number and length matching the period of the fluctuations in the oxidation state. **B. The oscillation of 5′UTR number and length matching the period of the fluctuations in the oxidation state.** **C. The oscillation of 3′UTR number and length matching the period of the fluctuations in the oxidation state.**
(DOC)

Figure S6 **The relationship between mean RNA transcript levels, total RNA transcript levels of** ***S. cerevisiae*** **genes and the redox cycle.** Yellow color (time points 1–4) is the oxidative phase, and the blue color (time points 5–12) is the reductive phase. Boxes show median values with statistical significance with notches (if two boxes' notches do not overlap this is a ‘strong evidence’ that their medians differ [2]), first quantile (25%) and third quantile (75%); whiskers indicate minimum and maximum values.
(DOC)

Figure S7 **The relationship between ORF length and the redox cycle of** ***S. cerevisiae*** **. Yellow color (time points 1–4) is the oxidative phase, and the blue color (time points 5–12) is the reductive phase.** Boxes show median values with statistical significance with notches (if two boxes' notches do not overlap this is a ‘strong evidence’ that their medians differ [2]), first quantile (25%) and third quantile (75%); whiskers indicate minimum and maximum values.
(DOC)

Figure S8 **The relationship between mean occurrence of a specific nucleotide in the third base in synonymous codons of** ***S. cerevisiae*** **genes and the redox cycle of** ***S. cerevisiae*** **.** Yellow color (time points 1–4) is the oxidative phase, and the blue color (time points 5–12) is the reductive phase. Boxes show median values with statistical significance with notches (if two boxes' notches do not overlap this is a ‘strong evidence’ that their medians differ [2]), first quantile (25%) and third quantile (75%); whiskers indicate minimum and maximum values.
(DOC)

Figure S9 **The relationship between CAI values and total log-normalized RNA expression level in the oxidative phase (time points 1–4) for each gene in** ***S. cerevisiae*** **.** Red points represent the measured values, and the black points represent the best non-linear fit.
(DOC)

Table S1 Table of mean CAI values for *S. cerevisiae*, *Saccharomyces sensu stricto* and *H. sapiens*, mean log-normalized RNA expressions, standard deviations, sample gene number, and two-tailed Wilcoxon *p*-values for oxidative and reductive phases of *S. cerevisiae* cycle.
(DOC)

Table S2 Table of mean pair wise phylogenetic sequence similarity values between *S. cerevisiae* and *Saccharomyces sensu stricto*, and between *S. cerevisiae* and *H. sapiens*, standard deviations, sample gene number, and two-tailed Wilcoxon *p*-values for oxidative and reductive phases of *S. cerevisiae* cycle.
(DOC)

Table S3 Table of mean frequencies of sequence variation per nucleotide over the time of divergence that resulted in the 63 strains (in ORFs, introns, 5′ and 3′UTRs), standard deviations, sample gene number, and two-tailed Wilcoxon *p*-values for oxidative and reductive phases of *S. cerevisiae* cycle.
(DOC)

Data S1 Supplementary data file contains open reading frames (ORFs), agreement about periodicity of gene expression between TRAC and YMC data sets, the periodicity of RNA expression and its false discovery rate (fdr), and ORF viability designation.
(PDF)

Appendix S1 (DOC)
