# Supplementary material for: Adaptation of Organisms by Resonance of RNA Transcription with the Cellular Redox Cycle
Source: PLoS One. 2011 Sep 28;6(9):e25270. doi: 10.1371/journal.pone.0025270 (PMC3182209; doi:10.1371/journal.pone.0025270)
Supplement: Figure S1 — The relationship between dissolved oxygen (blue curve), hydrogen sulfide (red curve) (from reference 1), codon adaptation index (CAI, black line) of sensu stricto clade genes ( S. paradoxus, S. mikatae, S. kudriavzevii, S. bayanus, S. castellii, S. kluyvery ) similar in amino acid sequences to S. cerevisiae ORFs expressed periodically during the redox cycle. Yellow color (time points 1–4) is the oxidative phase, and the blue color (time points 5–12) is the reductive phase. Boxes show median values with statistical significance with notches (if two boxes' notches do not overlap this is a ‘strong evidence’ that their medians differ [2]), first quantile (25%) and third quantile (75%); whiskers indicate minimum and maximum values. (DOC) [file pone.0025270.s001.doc]

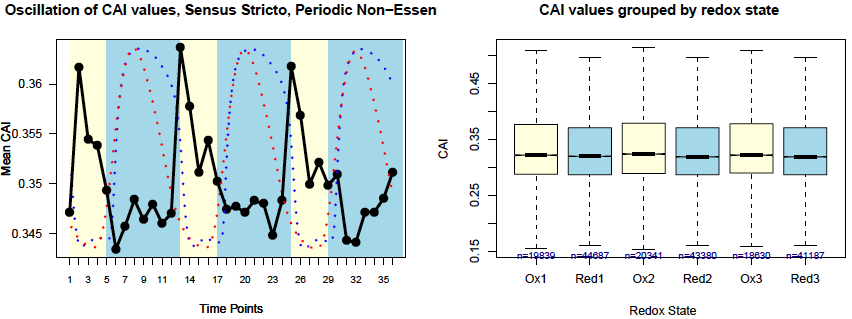


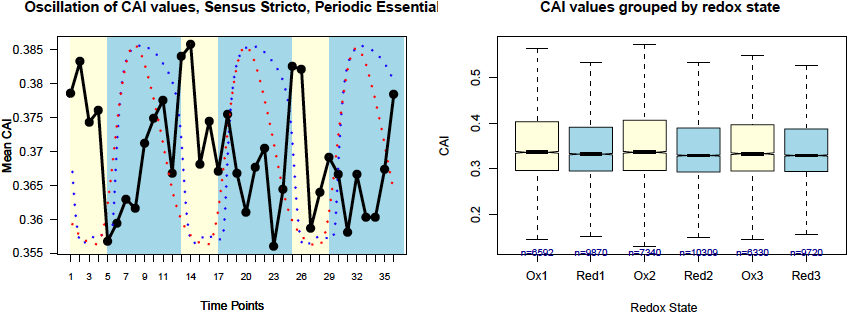


**Figure S1** The relationship between dissolved oxygen (blue curve), hydrogen sulfide (red curve) (from reference 1), codon adaptation index (CAI, black line) of *sensu stricto* clade genes (*S. paradoxus, S. mikatae, S. kudriavzevii, S. bayanus, S. castellii, S. kluyvery*) similar in amino acid sequences to *S. cerevisiae* ORFs expressed periodically during the redox cycle. Yellow color (time points 1-4) is the oxidative phase, and the blue color (time points 5-12) is the reductive phase. Boxes show median values with statistical significance with notches (if two boxes' notches do not overlap this is a ‘strong evidence’ that their medians differ (2)), first quantile (25%) and third quantile (75%); whiskers indicate minimum and maximum values.
