# Supplementary material for: Adaptation of Organisms by Resonance of RNA Transcription with the Cellular Redox Cycle
Source: PLoS One. 2011 Sep 28;6(9):e25270. doi: 10.1371/journal.pone.0025270 (PMC3182209; doi:10.1371/journal.pone.0025270)
Supplement: Figure S3 — The relationship between sequence similarity of H. sapiens genes to S. cerevisiae genes periodically expressed in the redox cycle. Yellow color (time points 3–6) is the suggested oxidative phase, and the blue color (time points 1–2 and 7–12) is the suggested reductive phase. Boxes show median values with statistical significance with notches (if two boxes' notches do not overlap this is a ‘strong evidence’ that their medians differ [2]), first quantile (25%) and third quantile (75%); whiskers indicate minimum and maximum values. (DOC) [file pone.0025270.s003.doc]

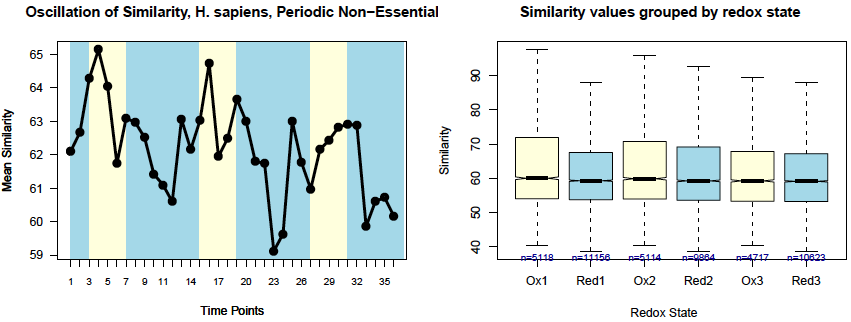


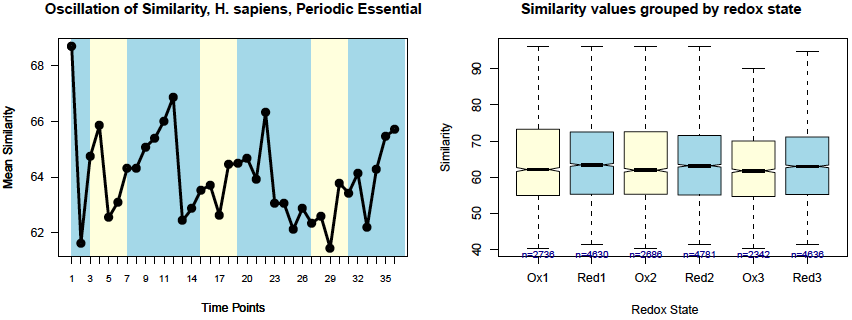


**Figure S3** The relationship between sequence similarity of *H. sapiens* genes to *S. cerevisiae* genes periodically expressed in the redox cycle. Yellow color (time points 3-6) is the suggested oxidative phase, and the blue color (time points 1-2 and 7-12) is the suggested reductive phase. Boxes show median values with statistical significance with notches (if two boxes' notches do not overlap this is a ‘strong evidence’ that their medians differ (2)), first quantile (25%) and third quantile (75%); whiskers indicate minimum and maximum values.
