# Supplementary material for: Adaptation of Organisms by Resonance of RNA Transcription with the Cellular Redox Cycle
Source: PLoS One. 2011 Sep 28;6(9):e25270. doi: 10.1371/journal.pone.0025270 (PMC3182209; doi:10.1371/journal.pone.0025270)
Supplement: Figure S4 — The relationship between dissolved oxygen (blue dots), hydrogen sulfide (red dots) (from reference 1), and the frequency of sequence variation during the redox cycle of yeast S. cerevisiae . The opposite phased oscillations for the frequency of sequence variation values in 5′UTRs and 3′UTRs (black lines) match the period of the fluctuations in the redox states. (DOC) [file pone.0025270.s004.doc]

**
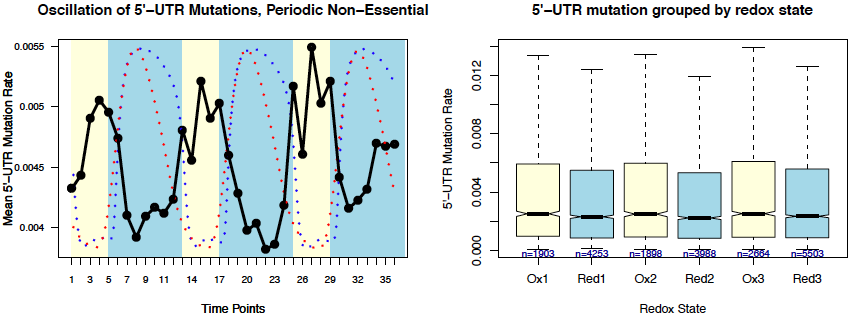
**

**
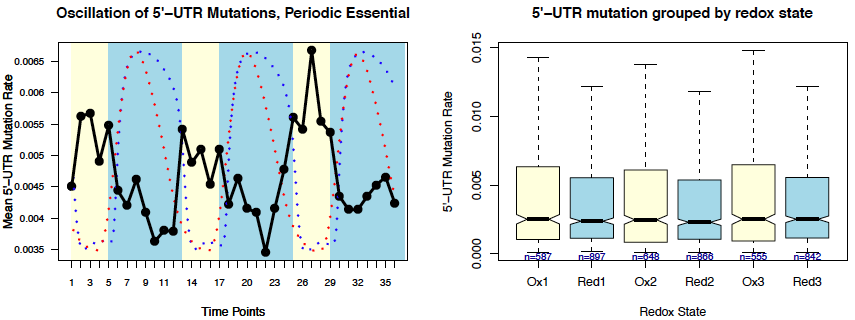
**

**
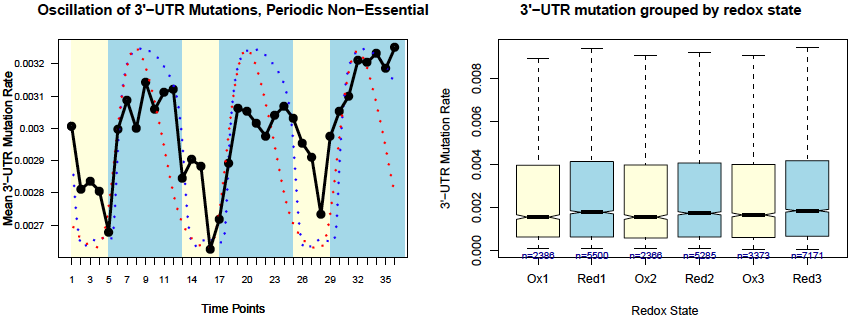
**

**
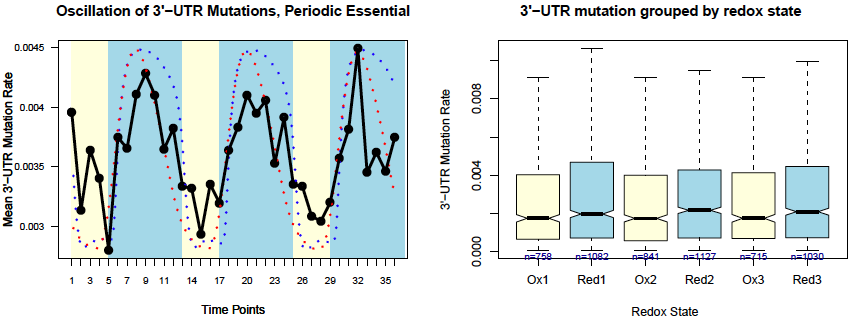
**

**Figure S4** The relationship between dissolved oxygen (blue dots), hydrogen sulfide (red dots) (from reference 1), and the frequency of sequence variation during the redox cycle of yeast *S. cerevisiae*. The opposite phased oscillations in the frequency of sequence variation values in 5’UTRs and 3’UTRs (black lines) match the period of the fluctuations in the redox states.
