# Supplementary material for: Adaptation of Organisms by Resonance of RNA Transcription with the Cellular Redox Cycle
Source: PLoS One. 2011 Sep 28;6(9):e25270. doi: 10.1371/journal.pone.0025270 (PMC3182209; doi:10.1371/journal.pone.0025270)
Supplement: Figure S5 — A. The relationship between number and length of introns, 5′ and 3′UTRs in S. cerevisiae genes and the redox cycle. Yellow color (time points 1–4) is the oxidative phase, and the blue color (time points 5–12) is the reductive phase. Boxes show median values with statistical significance with notches (if two boxes' notches do not overlap this is a ‘strong evidence’ that their medians differ [2]), first quantile (25%) and third quantile (75%); whiskers indicate minimum and maximum values. A. The oscillation of intron number and length matching the period of the fluctuations in the oxidation state. B. The oscillation of 5′UTR number and length matching the period of the fluctuations in the oxidation state. C. The oscillation of 3′UTR number and length matching the period of the fluctuations in the oxidation state. (DOC) [file pone.0025270.s005.doc]

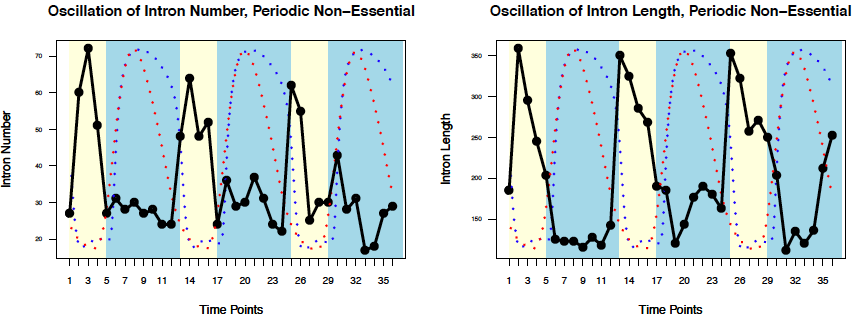


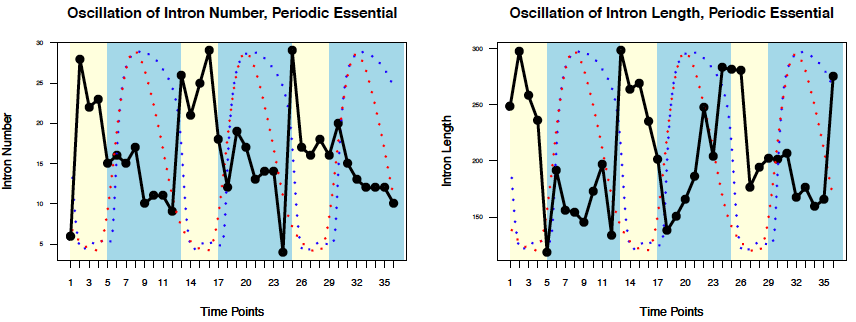


**Figure S5A**

**
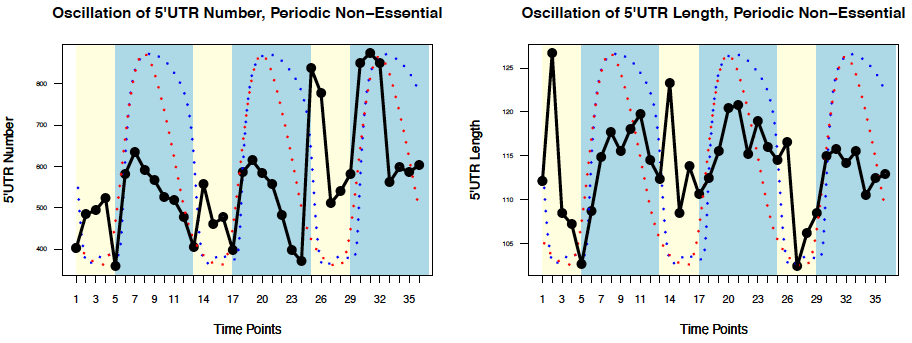
**

**
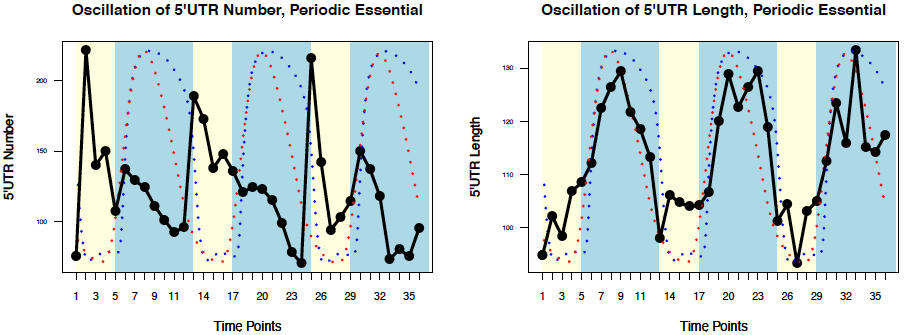
**

**Figure S5B**


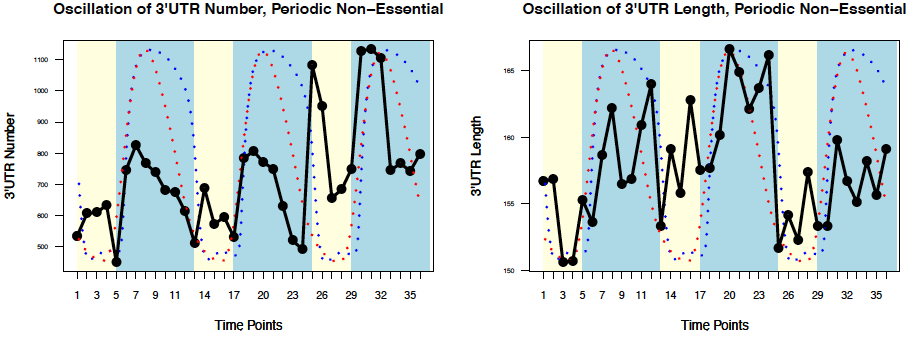


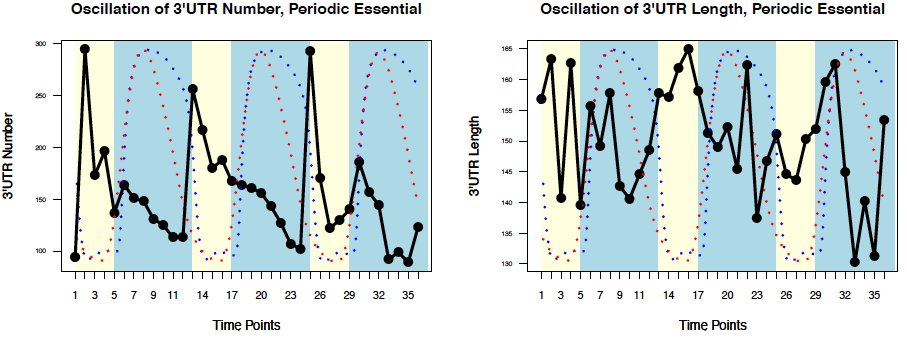


**Figure S5C**

**Figure S5** The relationship between number and length of introns, 5’ and 3’UTRs in *S. cerevisiae* genes and the redox cycle. Yellow color (time points 1-4) is the oxidative phase, and the blue color (time points 5-12) is the reductive phase. Boxes show median values with statistical significance with notches (if two boxes' notches do not overlap this is a ‘strong evidence’ that their medians differ (2)), first quantile (25%) and third quantile (75%); whiskers indicate minimum and maximum values. **A.** The oscillation of intron number and length matching the period of the fluctuations in the oxidation state. **B.** The oscillation of 5’UTR number and length matching the period of the fluctuations in the oxidation state. **C.** The oscillation of 3’UTR number and length matching the period of the fluctuations in the oxidation state.
