# Supplementary material for: Adaptation of Organisms by Resonance of RNA Transcription with the Cellular Redox Cycle
Source: PLoS One. 2011 Sep 28;6(9):e25270. doi: 10.1371/journal.pone.0025270 (PMC3182209; doi:10.1371/journal.pone.0025270)
Supplement: Figure S7 — The relationship between ORF length and the redox cycle of S. cerevisiae . Yellow color (time points 1–4) is the oxidative phase, and the blue color (time points 5–12) is the reductive phase. Boxes show median values with statistical significance with notches (if two boxes' notches do not overlap this is a ‘strong evidence’ that their medians differ [2]), first quantile (25%) and third quantile (75%); whiskers indicate minimum and maximum values. (DOC) [file pone.0025270.s007.doc]

**
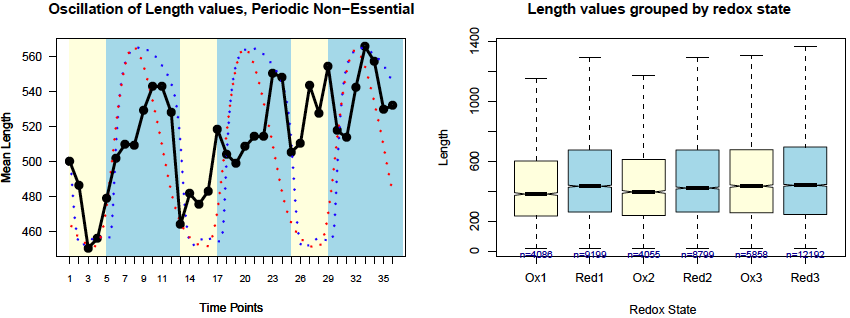
**

**
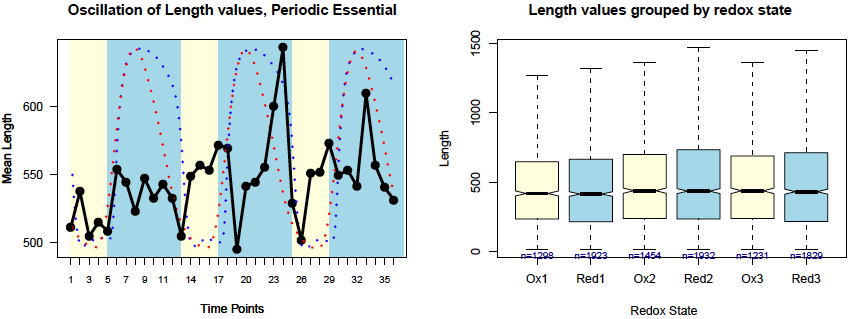
**

**Figure S7** The relationship between ORF length and the redox cycle of *S. cerevisiae*. Yellow color (time points 1-4) is the oxidative phase, and the blue color (time points 5-12) is the reductive phase. Boxes show median values with statistical significance with notches (if two boxes' notches do not overlap this is a ‘strong evidence’ that their medians differ (2)), first quantile (25%) and third quantile (75%); whiskers indicate minimum and maximum values.
