# Supplementary material for: Adaptation of Organisms by Resonance of RNA Transcription with the Cellular Redox Cycle
Source: PLoS One. 2011 Sep 28;6(9):e25270. doi: 10.1371/journal.pone.0025270 (PMC3182209; doi:10.1371/journal.pone.0025270)
Supplement: Figure S9 — The relationship between CAI values and total log-normalized RNA expression level in the oxidative phase (time points 1–4) for each gene in S. cerevisiae . Red points represent the measured values, and the black points represent the best non-linear fit. (DOC) [file pone.0025270.s009.doc]

**
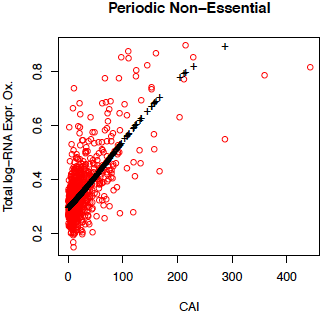

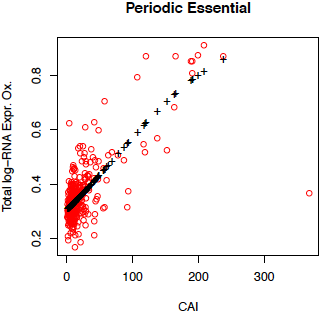
**

**Figure S9** The relationship between CAI values and total log-normalized RNA expression level in the oxidative phase (time points 1-4) for each gene in *S. cerevisiae*. Red points represent the measured values, and the black points represent the best non-linear fit.
