# Supplementary material for: Adaptation of Organisms by Resonance of RNA Transcription with the Cellular Redox Cycle
Source: PLoS One. 2011 Sep 28;6(9):e25270. doi: 10.1371/journal.pone.0025270 (PMC3182209; doi:10.1371/journal.pone.0025270)
Supplement: Table S1 — Table of mean CAI values for S. cerevisiae, Saccharomyces sensu stricto and H. sapiens, mean log-normalized RNA expressions, standard deviations, sample gene number, and two-tailed Wilcoxon p-values for oxidative and reductive phases of S. cerevisiae cycle. (DOC) [file pone.0025270.s010.doc]

|  | Oxidative (1-4) | Reductive (5-12) |
| --- | --- | --- |
| CAI Non-Ess | 0.362±0.135(SD, N=4086) | 0.336±0.093(SD, N=9199) |
|  | 0.369±0.140(SD, N=4055) | 0.337±0.099(SD, N=8799) |
|  | 0.363±0.134(SD, N=5858) | 0.339±0.103(SD, N=12192) |
|  | p-value = 8.993e-13, p-value < 2.2e-16, p-value < 2.2e-16 | |
| CAI Ess | 0.386± 0.151(SD, N=1298) | 0.360± 0.121(SD, N=1923) |
|  | 0.395± 0.150(SD, N=1454) | 0.364± 0.130(SD, N=1932) |
|  | 0.387± 0.149(SD, N=1231) | 0.359± 0.126(SD, N=1829) |
|  | p-value = 9.467e-05, p-value = 9.16e-11, p-value = 7.326e-08 | |
| CAI 6 Non-Ess | 0.355± 0.120(SD, N=19839) | 0.347± 0.108(SD, N=44687) |
|  | 0.357± 0.121(SD, N=20341) | 0.348± 0.110(SD, N=43380) |
|  | 0.356± 0.121(SD, N=18630) | 0.348± 0.111(SD, N=41187) |
|  | p-value = 1.581e-06, p-value = 8.553e-13, p-value = 4.601e-10 | |
| CAI 6 Ess | 0.379± 0.142(SD, N=6592) | 0.366± 0.125(SD, N=9870) |
|  | 0.379± 0.140(SD, N=7340) | 0.367± 0.133(SD, N=10309) |
|  | 0.375± 0.140(SD, N=6330) | 0.366± 0.132(SD, N=9720) |
|  | p-value = 2.517e-05, p-value = 1.596e-10, p-value = 4.748e-05 | |
| CAI Hum Non-Ess | 0.380± 0.144(SD, N=3960) | 0.370± 0.142(SD, N=7127) |
|  | 0.386± 0.145(SD, N=4056) | 0.370± 0.141(SD, N=6851) |
|  | 0.388± 0.146(SD, N=3668) | 0.372± 0.140(SD, N=6986) |
|  | p-value = 0.0001643, p-value = 3.888e-09, p-value = 2.857e-07 | |
| CAI Hum Ess | 0.403± 0.151(SD, N=2109) | 0.375± 0.153(SD, N=3570) |
|  | 0.407± 0.149(SD, N=2150) | 0.375± 0.148(SD, N=3894) |
|  | 0.409± 0.153(SD, N=1954) | 0.379± 0.153(SD, N=3801) |
|  | p-value = 6.357e-13, p-value < 2.2e-16, p-value = 8.238e-14 | |
| Expr Non-Ess | 7.081± 12.334(SD, N=4086) | 5.624± 8.777(SD, N=9199) |
|  | 6.880± 10.354(SD, N=4055) | 5.190± 8.004(SD, N=8799) |
|  | 6.559± 9.938(SD, N=5858) | 4.805± 7.312(SD, N=12192) |
|  | p-value = 0.04752, p-value = 3.553e-15, p-value < 2.2e-16 | |
| Expr Ess | 7.223± 12.153(SD, N=1298) | 7.313± 10.969(SD, N=1923) |
|  | 7.083± 9.213(SD, N=1454) | 6.355± 9.003(SD, N=1932) |
|  | 7.754± 9.876(SD, N=1231) | 5.846± 8.231(SD, N=1829) |
|  | p-value = 0.003922, p-value = 7.017e-06, p-value = 4.986e-12 | |

**Table S1.** Table of mean CAI values for *S. cerevisiae*, *Saccharomyces sensu stricto* and *H. sapiens*, mean log-normalized RNA expressions, standard deviations, sample gene number, and two-tailed Wilcoxon *p*-values for oxidative and reductive phases of *S. cerevisiae* cycle.
