# Supplementary material for: Adaptation of Organisms by Resonance of RNA Transcription with the Cellular Redox Cycle
Source: PLoS One. 2011 Sep 28;6(9):e25270. doi: 10.1371/journal.pone.0025270 (PMC3182209; doi:10.1371/journal.pone.0025270)
Supplement: Table S2 — Table of mean pair wise phylogenetic sequence similarity values between S. cerevisiae and Saccharomyces sensu stricto, and between S. cerevisiae and H. sapiens, standard deviations, sample gene number, and two-tailed Wilcoxon p-values for oxidative and reductive phases of S. cerevisiae cycle. (DOC) [file pone.0025270.s011.doc]

|  | Oxidative (1-4) | Reductive (5-12) |
| --- | --- | --- |
| Sim 6 Non-Ess | 79.317± 15.457(SD, N=30969) | 77.012± 15.717(SD, N=70862) |
|  | 78.780± 15.556(SD, N=32321) | 77.063± 15.835(SD, N=67239) |
|  | 78.435± 15.580(SD, N=28723) | 77.170± 15.711(SD, N=66147) |
|  | p-value < 2.2e-16, p-value < 2.2e-16, p-value < 2.2e-16 | |
| Sim 6 Ess | 80.222± 16.389(SD, N=8181) | 80.027± 16.189(SD, N=14965) |
|  | 81.222± 16.079(SD, N=8110) | 80.215± 16.177(SD, N=16051) |
|  | 80.577± 15.970(SD, N=6968) | 79.752± 16.389(SD, N=15088) |
|  | p-value = 0.4141, p-value = 2.078e-05, p-value = 0.01838 | |
| Sim Hum Non-Ess | 63.681± 13.257(SD, N=5118) | 62.125± 12.094(SD, N=11156) |
|  | 63.074± 12.907(SD, N=5114) | 62.017± 12.128(SD, N=9864) |
|  | 62.173± 12.961(SD, N=4717) | 61.633± 12.008(SD, N=10623) |
|  | p-value = 5.221e-08, p-value = 0.0002054, p-value = 0.4764 | |
| Sim Hum Ess | 64.210± 12.530(SD, N=2736) | 64.629± 12.161(SD, N=4630) |
|  | 63.651± 11.612(SD, N=2686) | 63.790± 11.484(SD, N=4781) |
|  | 62.726± 11.322(SD, N=2342) | 63.460± 10.933(SD, N=4636) |
|  | p-value = 0.05138, p-value = 0.3582, p-value = 0.001529 | |

**Table S2.** Table of mean pair wise phylogenetic sequence similarity values between *S. cerevisiae* and *Saccharomyces sensu stricto*, and between *S. cerevisiae* and *H. sapiens*, standard deviations, sample gene number, and two-tailed Wilcoxon *p*-values for oxidative and reductive phases of *S. cerevisiae* cycle.
