# Supplementary material for: Adaptation of Organisms by Resonance of RNA Transcription with the Cellular Redox Cycle
Source: PLoS One. 2011 Sep 28;6(9):e25270. doi: 10.1371/journal.pone.0025270 (PMC3182209; doi:10.1371/journal.pone.0025270)
Supplement: Table S3 — Table of mean frequencies of sequence variation per nucleotide over the time of divergence that resulted in the 63 strains (in ORFs, introns, 5′ and 3′UTRs), standard deviations, sample gene number, and two-tailed Wilcoxon p-values for oxidative and reductive phases of S. cerevisiae cycle. (DOC) [file pone.0025270.s012.doc]

| Frequency of sequence variation | Oxidative (1-4) | | Reductive (5-12) |
| --- | --- | --- | --- |
| ORFs Non-Ess | 0.00155± 0.00104(SD, N=4086) | | 0.00165± 0.00104(SD, N=9199) |
|  | 0.00153± 0.00102(SD, N=4055) | | 0.00162± 0.00102(SD, N=8799) |
|  | 0.00153± 0.00102(SD, N=5857) | | 0.00161± 0.00103(SD, N=12184) |
|  | p-value = 2.464e-09, p-value = 2.205e-06, p-value = 1.020e-06 | | |
| ORFs Ess | 0.00140± 0.00101(SD, N=1296) | | 0.00148± 0.00102(SD, N=1915) |
|  | 0.00140± 0.00100(SD, N=1452) | | 0.00150± 0.00103(SD, N=1925) |
|  | 0.00140± 0.00100(SD, N=1230) | | 0.00146± 0.00101(SD, N=1821) |
|  | p-value = 0.009672, p-value = 0.003585, p-value = 0.07197 | | |
| Introns Non-Ess | 0.00251± 0.00234(SD, N=210) | | 0.00157± 0.00202(SD, N=219) |
|  | 0.00252± 0.00227(SD, N=212) | | 0.00177± 0.00212(SD, N=233) |
|  | 0.00284± 0.00283(SD, N=274) | | 0.00212± 0.00261(SD, N=377) |
|  | p-value = 4.869e-07, p-value = 3.776e-06, p-value = 4.305e-06 | | |
| Introns Ess | 0.00331± 0.00335(SD, N=79) | 0.00270± 0.00343(SD, N=104) | |
|  | 0.00315± 0.00302(SD, N=101) | 0.00226± 0.00260(SD, N=111) | |
|  | 0.00355± 0.00362(SD, N=80) | 0.00288± 0.00322(SD, N=110) | |
|  | p-value = 0.02609, p-value = 0.003695, p-value = 0.07791 | | |
| 5’-UTRs Non-Ess | 0.00470± 0.00619(SD, N=1903) | 0.00426± 0.00545(SD, N=4253) | |
|  | 0.00486± 0.00657(SD, N=1898) | 0.00422± 0.00559(SD, N=3988) | |
|  | 0.00504± 0.00785(SD, N=2664) | 0.00451± 0.00685(SD, N=5503) | |
|  | p-value = 0.02955, p-value = 0.001531, p-value = 0.03248 | | |
| 5’-UTRs Ess | 0.00531± 0.00867(SD, N=587) | 0.00428± 0.00486(SD, N=897) | |
|  | 0.00500± 0.00756(SD, N=648) | 0.00434± 0.00615(SD, N=900) | |
|  | 0.00572± 0.01013(SD, N=555) | 0.00445± 0.00509(SD, N=842) | |
|  | p-value = 0.543, p-value = 0.911, p-value = 0.9117 | | |
| 3’-UTRs Non-Ess | 0.00286± 0.00339(SD, N=2386) | 0.00304± 0.00359(SD, N=5500) | |
|  | 0.00282± 0.00317(SD, N=2366) | 0.00298± 0.00354(SD, N=5285) | |
|  | 0.00275± 0.00303(SD, N=2067) | 0.00307± 0.00357(SD, N=5016) | |
|  | p-value = 0.09204, p-value = 0.1748, p-value = 0.009627 | | |
| 3’-UTRs Ess | 0.00342± 0.00635(SD, N=758) | 0.00376± 0.00743(SD, N=1082) | |
|  | 0.00325± 0.00523(SD, N=841) | 0.00376± 0.00734(SD, N=1127) | |
|  | 0.00324± 0.00410(SD, N=715) | 0.00369± 0.00643(SD, N=1030) | |
|  | p-value = 0.05439, p-value = 0.01068, p-value = 0.07224 | | |

**Table S3.** Table of mean frequencies of sequence variation per nucleotide over the time of divergence that resulted in the 63 strains (in ORFs, introns, 5’ and 3’UTRs), standard deviations, sample gene number, and two-tailed Wilcoxon *p*-values for the oxidative and reductive phases of *S. cerevisiae* redox cycle.
