# Supplementary material for: Adaptation of Organisms by Resonance of RNA Transcription with the Cellular Redox Cycle
Source: PLoS One. 2011 Sep 28;6(9):e25270. doi: 10.1371/journal.pone.0025270 (PMC3182209; doi:10.1371/journal.pone.0025270)
Supplement: Data S1 — Supplementary data file contains open reading frames (ORFs), agreement about periodicity of gene expression between TRAC and YMC data sets, the periodicity of RNA expression and its false discovery rate (fdr), and ORF viability designation. (PDF) [file pone.0025270.s013.pdf]

Supplementary data contains open reading frames (ORFs), agreement about periodicity of gene expression between TRAC and YMC data sets, the periodicity of RNA expression and its false discovery rate (fdr), and ORF viability designation.

	ORF	Agreement	Periodic	Viability	fdrPeriodicity
1	YDL039C	No agreement	TRUE	1	0.208
2	YBR105C	No agreement	TRUE	1	0.062
3	YDR481C	No agreement	FALSE	1	0.216
4	YLL053C	No agreement	FALSE	1	0
5	YBR043C	No agreement	FALSE	1	0
6	YOL119C	No agreement	FALSE	1	0
7	YKL207W	No agreement	FALSE	1	0.275
8	YLL012W	No agreement	FALSE	1	0
9	YJR013W	Agreement	FALSE	0	0.116
10	YLL052C	No agreement	FALSE	1	0
11	YDR492W	No agreement	FALSE	1	0.093
12	YPR058W	No agreement	FALSE	1	0.007
13	YGL067W	No agreement	FALSE	1	0
14	YEL046C	No agreement	FALSE	1	0
15	YBR183W	Agreement	TRUE	1	0
16	YLR414C	Agreement	FALSE	1	0.014
17	YJR152W	No agreement	FALSE	1	0.091
18	YKL084W	No agreement	FALSE	1	0.104
19	YPR128C	No agreement	FALSE	1	0
20	YGR260W	No agreement	FALSE	1	0.086
21	YNR043W	Agreement	FALSE	0	0
22	YOL002C	No agreement	FALSE	1	0
23	YNR041C	Agreement	FALSE	1	0
24	YJR019C	Agreement	FALSE	1	0
25	YNL046W	No agreement	FALSE	1	0
26	YNL145W	No agreement	FALSE	1	0.125
27	YLR050C	Agreement	FALSE	1	0.012
28	YHR050W	Agreement	FALSE	1	0
29	YML075C	Agreement	FALSE	1	0
30	YMR015C	No agreement	FALSE	1	0.022
31	YBR145W	Agreement	FALSE	1	0.007
32	YBL102W	No agreement	FALSE	1	0
33	YNL066W	No agreement	FALSE	1	0
34	YLR220W	No agreement	FALSE	1	0.271
35	YBL098W	No agreement	FALSE	1	0.281
36	YHR007C	No agreement	FALSE	0	0
37	YPL028W	Agreement	FALSE	0	0.008
38	YMR221C	No agreement	FALSE	1	0.172
39	YMR303C	No agreement	FALSE	1	0
40	YLR231C	Agreement	FALSE	1	0.227
41	YMR202W	No agreement	FALSE	1	0.009
42	YDR384C	No agreement	FALSE	1	0
43	YKL008C	No agreement	FALSE	1	0.071
44	YMR272C	No agreement	FALSE	1	0.064

45	YDR284C	Agreement	FALSE	1	0.185
46	YPR011C	No agreement	TRUE	0	0
47	YLR332W	No agreement	FALSE	1	0
48	YGR149W	Agreement	FALSE	1	0
49	YLR099C	No agreement	TRUE	1	0.001
50	YAL053W	No agreement	FALSE	1	0.032
51	YMR155W	Agreement	FALSE	1	0.022
52	YNL156C	No agreement	TRUE	1	0.055
53	YHR181W	No agreement	FALSE	1	0.068
54	YJR016C	No agreement	FALSE	0	0
55	YGL001C	No agreement	FALSE	0	0
56	YNR019W	No agreement	TRUE	1	0
57	YOL147C	Agreement	FALSE	1	0
58	YGR101W	No agreement	FALSE	1	0.001
59	YBL089W	No agreement	FALSE	1	0.12
60	YER158C	No agreement	FALSE	1	0
61	YHR143W	No agreement	FALSE	1	0
62	YGL202W	No agreement	FALSE	1	0
63	YML126C	Agreement	FALSE	0	0
64	YKR013W	Agreement	TRUE	1	0
65	YGL198W	Agreement	FALSE	1	0.161
66	YPR176C	No agreement	FALSE	0	0.002
67	YIL146C	No agreement	FALSE	1	0
68	YLL051C	No agreement	FALSE	1	0.06
69	YPL061W	No agreement	TRUE	1	0
70	YLR375W	No agreement	FALSE	1	0.099
71	YDR487C	No agreement	FALSE	0	0.035
72	YNL111C	No agreement	TRUE	0	0.001
73	YJL004C	Agreement	TRUE	1	0
74	YAL054C	No agreement	TRUE	1	0
75	YNL280C	No agreement	FALSE	1	0
76	YBR243C	No agreement	TRUE	0	0
77	YGR289C	Agreement	FALSE	0	0
78	YIL006W	Agreement	FALSE	1	0.004
79	YLR034C	Agreement	FALSE	1	0.197
80	YKL218C	Agreement	FALSE	1	0.002
81	YLL028W	No agreement	FALSE	1	0
82	YDR044W	No agreement	TRUE	0	0
83	YGR110W	No agreement	TRUE	1	0
84	YMR096W	Agreement	TRUE	1	0
85	YGL028C	No agreement	FALSE	1	0
86	YNL329C	Agreement	TRUE	1	0
87	YJR116W	Agreement	FALSE	1	0.283
88	YLR237W	No agreement	TRUE	1	0
89	YHR190W	Agreement	TRUE	0	0
90	YNL237W	Agreement	FALSE	1	0
91	YDR297W	No agreement	TRUE	1	0
92	YLR120C	No agreement	FALSE	1	0.042
93	YDR072C	Agreement	FALSE	1	0.004

94	YDR142C	Agreement	TRUE	1	0.001
95	YOR328W	No agreement	FALSE	1	0
96	YDL248W	No agreement	FALSE	0	0
97	YDR084C	Agreement	FALSE	1	0.004
98	YFL062W	Agreement	FALSE	0	0
99	YGR125W	No agreement	FALSE	1	0.001
100	YBR110W	Agreement	FALSE	0	0.072
101	YNL104C	No agreement	FALSE	1	0
102	YDR100W	Agreement	FALSE	1	0
103	YBR230C	No agreement	FALSE	1	0
104	YPL179W	No agreement	FALSE	1	0.001
105	YDL198C	Agreement	FALSE	1	0
106	YLR027C	Agreement	TRUE	1	0.001
107	YDR105C	Agreement	FALSE	1	0.202
108	YDR107C	Agreement	FALSE	1	0.021
109	YPR198W	No agreement	FALSE	1	0.003
110	YJL001W	Agreement	TRUE	0	0
111	YGL255W	No agreement	FALSE	1	0.231
112	YKL093W	Agreement	FALSE	1	0
113	YMR266W	No agreement	TRUE	1	0
114	YHR072W	No agreement	TRUE	0	0.001
115	YDR438W	Agreement	FALSE	1	0
116	YKR088C	Agreement	FALSE	1	0.057
117	YDR349C	No agreement	FALSE	1	0.264
118	YOR002W	Agreement	FALSE	1	0
119	YGR175C	No agreement	TRUE	0	0.016
120	YCR044C	No agreement	FALSE	1	0.003
121	YLR113W	No agreement	FALSE	1	0.001
122	YDR055W	No agreement	FALSE	1	0
123	YER145C	No agreement	FALSE	1	0
124	YLR241W	Agreement	FALSE	1	0
125	YDR411C	Agreement	FALSE	1	0
126	YOR011W	No agreement	FALSE	1	0
127	YDR011W	No agreement	FALSE	1	0
128	YPR196W	Agreement	FALSE	1	0
129	YDR387C	No agreement	FALSE	1	0.177
130	YBR008C	No agreement	TRUE	1	0
131	YGL002W	Agreement	TRUE	1	0.001
132	YNL305C	Agreement	FALSE	1	0
133	YNR050C	Agreement	TRUE	1	0
134	YKR030W	No agreement	FALSE	1	0.08
135	YHR096C	No agreement	FALSE	1	0
136	YJL185C	Agreement	FALSE	1	0
137	YER124C	No agreement	FALSE	1	0
138	YLR019W	Agreement	FALSE	1	0.173
139	YCR075C	Agreement	FALSE	1	0.013
140	YLR153C	Agreement	FALSE	0	0.003
141	YBR036C	Agreement	FALSE	1	0
142	YOR099W	Agreement	FALSE	1	0.006

143	YDL142C	Agreement	TRUE	1	0
144	YHR132C	Agreement	FALSE	1	0.207
145	YLR142W	No agreement	TRUE	1	0.049
146	YKL176C	No agreement	TRUE	1	0.031
147	YCR069W	Agreement	FALSE	1	0
148	YAL061W	Agreement	FALSE	1	0
149	YDR247W	Agreement	FALSE	1	0
150	YHR074W	Agreement	FALSE	0	0.001
151	YGR268C	No agreement	TRUE	1	0.12
152	YPR106W	Agreement	FALSE	1	0
153	YDR476C	Agreement	FALSE	1	0.017
154	YDL054C	No agreement	FALSE	1	0
155	YEL017C-A	No agreement	FALSE	1	0
156	YBR212W	Agreement	FALSE	1	0
157	YOR228C	Agreement	FALSE	1	0.002
158	YPR147C	No agreement	TRUE	1	0.001
159	YPR149W	Agreement	FALSE	1	0
160	YEL017W	Agreement	TRUE	1	0
161	YNL058C	Agreement	FALSE	1	0
162	YDR059C	Agreement	TRUE	1	0
163	YOR348C	Agreement	FALSE	1	0
164	YCL038C	Agreement	FALSE	1	0
165	YKR055W	Agreement	TRUE	1	0
166	YJL048C	Agreement	TRUE	1	0
167	YGL161C	Agreement	TRUE	1	0.003
168	YGL026C	No agreement	FALSE	1	0.003
169	YGL101W	Agreement	TRUE	1	0
170	YOL122C	Agreement	FALSE	1	0.017
171	YLL019C	Agreement	FALSE	1	0
172	YNL176C	No agreement	FALSE	1	0.014
173	YPR113W	No agreement	FALSE	0	0
174	YOR222W	No agreement	TRUE	1	0
175	YDL048C	No agreement	TRUE	1	0.001
176	YDL123W	Agreement	FALSE	1	0
177	YER152C	Agreement	FALSE	1	0.025
178	YIR034C	Agreement	TRUE	1	0
179	YIL011W	Agreement	TRUE	1	0.001
180	YNL307C	Agreement	FALSE	1	0
181	YPR154W	Agreement	FALSE	1	0
182	YOR044W	No agreement	TRUE	1	0
183	YDR435C	Agreement	TRUE	1	0
184	YLL023C	Agreement	FALSE	1	0
185	YDR264C	Agreement	TRUE	1	0.032
186	YKL019W	No agreement	FALSE	0	0.214
187	YCR061W	Agreement	FALSE	1	0
188	YDR456W	Agreement	FALSE	1	0.035
189	YPL229W	No agreement	FALSE	1	0.011
190	YKR046C	Agreement	FALSE	1	0
191	YDL024C	Agreement	FALSE	1	0.003

192	YMR181C	Agreement	FALSE	1	0
193	YIL013C	Agreement	FALSE	1	0.095
194	YMR166C	No agreement	TRUE	1	0
195	YDL237W	No agreement	FALSE	1	0.137
196	YLR286C	Agreement	TRUE	1	0
197	YGR062C	No agreement	FALSE	1	0
198	YML116W	No agreement	TRUE	1	0
199	YKL062W	Agreement	TRUE	1	0.002
200	YFL041W	No agreement	FALSE	1	0
201	YBR222C	No agreement	FALSE	1	0
202	YBR115C	No agreement	TRUE	1	0
203	YLR134W	No agreement	FALSE	1	0.151
204	YOL001W	Agreement	FALSE	1	0.003
205	YMR003W	No agreement	TRUE	1	0
206	YDL165W	No agreement	FALSE	0	0.247
207	YOR003W	Agreement	TRUE	1	0
208	YIL153W	Agreement	TRUE	1	0
209	YPL087W	No agreement	FALSE	1	0
210	YNL183C	Agreement	FALSE	1	0.283
211	YGR106C	No agreement	FALSE	1	0.099
212	YOR202W	No agreement	TRUE	1	0
213	YML051W	No agreement	TRUE	1	0.002
214	YCL018W	Agreement	FALSE	0	0
215	YIL094C	No agreement	TRUE	0	0
216	YDL171C	No agreement	FALSE	1	0.07
217	YBL095W	Agreement	TRUE	1	0.001
218	YLR209C	No agreement	FALSE	1	0
219	YHR140W	Agreement	FALSE	1	0
220	YMR013C	No agreement	TRUE	0	0.005
221	YJL151C	Agreement	TRUE	1	0.001
222	YER044C	Agreement	TRUE	1	0
223	YDR262W	No agreement	TRUE	1	0
224	YJR103W	Agreement	FALSE	1	0
225	YPL224C	Agreement	TRUE	1	0.012
226	YKL164C	No agreement	FALSE	1	0
227	YKR065C	No agreement	FALSE	1	0
228	YBR139W	Agreement	FALSE	1	0
229	YDR451C	Agreement	TRUE	1	0
230	YJL059W	Agreement	FALSE	1	0
231	YEL052W	No agreement	FALSE	1	0
232	YLR403W	No agreement	TRUE	1	0.089
233	YDR313C	Agreement	TRUE	1	0
234	YGR077C	Agreement	TRUE	1	0
235	YIL034C	Agreement	TRUE	1	0.114
236	YEL038W	No agreement	FALSE	1	0.036
237	YOL003C	No agreement	FALSE	1	0.127
238	YGL040C	No agreement	FALSE	0	0
239	YGL012W	No agreement	TRUE	1	0
240	YOL136C	No agreement	FALSE	1	0.018

241	YDL206W	Agreement	FALSE	1	0
242	YDL072C	Agreement	FALSE	1	0
243	YLR194C	No agreement	TRUE	1	0
244	YDR205W	Agreement	FALSE	1	0.253
245	YHR188C	No agreement	FALSE	0	0.008
246	YMR208W	Agreement	TRUE	0	0.009
247	YDL086W	Agreement	FALSE	1	0
248	YLR297W	No agreement	TRUE	1	0
249	YDR234W	Agreement	TRUE	1	0
250	YPR074C	Agreement	FALSE	1	0
251	YIL101C	Agreement	FALSE	1	0
252	YBR210W	Agreement	TRUE	1	0.231
253	YBR280C	Agreement	TRUE	1	0
254	YHR202W	Agreement	TRUE	1	0
255	YBR295W	Agreement	FALSE	1	0
256	YMR191W	Agreement	FALSE	1	0
257	YEL004W	No agreement	FALSE	1	0.283
258	YBR046C	No agreement	TRUE	1	0
259	YPR013C	No agreement	TRUE	1	0
260	YOR385W	No agreement	FALSE	1	0.033
261	YDR406W	Agreement	FALSE	1	0.02
262	YFL020C	Agreement	FALSE	1	0.039
263	YJL093C	Agreement	TRUE	1	0.015
264	YJR065C	Agreement	FALSE	0	0.001
265	YFR050C	Agreement	TRUE	0	0.001
266	YLR201C	Agreement	FALSE	1	0
267	YNL275W	Agreement	FALSE	1	0
268	YFL052W	No agreement	TRUE	1	0
269	YHR047C	No agreement	TRUE	1	0
270	YLR100W	No agreement	TRUE	0	0
271	YDR256C	Agreement	TRUE	1	0
272	YGL146C	No agreement	FALSE	1	0.234
273	YDR415C	No agreement	TRUE	1	0
274	YMR006C	Agreement	TRUE	1	0
275	YNL270C	No agreement	TRUE	1	0
276	YHR028C	Agreement	FALSE	1	0.234
277	YBR132C	Agreement	FALSE	1	0
278	YPL067C	No agreement	FALSE	1	0
279	YMR119W	Agreement	TRUE	1	0
280	YNL195C	Agreement	FALSE	1	0
281	YDR367W	No agreement	FALSE	0	0.288
282	YBR241C	Agreement	FALSE	1	0
283	YOR034C	Agreement	FALSE	1	0
284	YKL192C	No agreement	FALSE	0	0
285	YMR041C	Agreement	TRUE	1	0
286	YGR143W	Agreement	FALSE	1	0
287	YPR028W	Agreement	FALSE	1	0
288	YOR071C	No agreement	FALSE	1	0.052
289	YGL160W	Agreement	TRUE	1	0

290	YER119C	Agreement	TRUE	1	0.037
291	YHR198C	No agreement	TRUE	1	0
292	YLR356W	Agreement	TRUE	1	0
293	YLR299W	Agreement	FALSE	1	0.151
294	YGR108W	Agreement	TRUE	1	0
295	YOR292C	Agreement	FALSE	1	0.008
296	YDR504C	Agreement	TRUE	1	0
297	YGL020C	No agreement	TRUE	1	0.006
298	YAR019C	Agreement	TRUE	0	0.014
299	YPL147W	Agreement	TRUE	1	0
300	YML042W	No agreement	TRUE	1	0
301	YNR055C	Agreement	FALSE	1	0.229
302	YDR539W	Agreement	FALSE	1	0.098
303	YDR505C	Agreement	TRUE	1	0
304	YAR035W	No agreement	TRUE	1	0
305	YDR309C	Agreement	FALSE	1	0
306	YKR087C	Agreement	FALSE	1	0.189
307	YBR147W	No agreement	TRUE	1	0
308	YPL183W-A	No agreement	FALSE	1	0
309	YGL053W	Agreement	TRUE	1	0
310	YAL016W	Agreement	TRUE	1	0.006
311	YDR373W	No agreement	TRUE	0	0.054
312	YLR377C	No agreement	FALSE	1	0
313	YBL049W	Agreement	FALSE	1	0
314	YAL049C	Agreement	TRUE	1	0
315	YLR438W	Agreement	FALSE	1	0.001
316	YBR256C	No agreement	TRUE	0	0
317	YOR140W	No agreement	FALSE	1	0.059
318	YOR262W	No agreement	TRUE	0	0.023
319	YMR246W	No agreement	TRUE	1	0
320	YDL199C	Agreement	FALSE	1	0
321	YER141W	Agreement	FALSE	1	0
322	YNL220W	Agreement	TRUE	0	0
323	YOR152C	Agreement	TRUE	1	0
324	YMR318C	No agreement	FALSE	1	0.119
325	YML092C	Agreement	TRUE	0	0.016
326	YKR005C	Agreement	FALSE	1	0.004
327	YOR286W	No agreement	TRUE	1	0
328	YJR127C	Agreement	TRUE	1	0.091
329	YDL022W	Agreement	TRUE	1	0
330	YNR032W	Agreement	TRUE	1	0.015
331	YNL011C	Agreement	TRUE	1	0
332	YKR097W	Agreement	FALSE	1	0
333	YFR018C	Agreement	FALSE	1	0.256
334	YFR024C-A	Agreement	TRUE	1	0.005
335	YGR124W	Agreement	FALSE	1	0
336	YCR011C	Agreement	FALSE	1	0.001
337	YJL137C	Agreement	TRUE	1	0
338	YBR291C	Agreement	TRUE	1	0

339	YJL132W	Agreement	FALSE	1	0
340	YIL042C	Agreement	TRUE	1	0.001
341	YBR214W	Agreement	FALSE	1	0
342	YMR297W	Agreement	FALSE	1	0
343	YLR337C	Agreement	TRUE	1	0.003
344	YIR019C	Agreement	TRUE	1	0.109
345	YKR052C	Agreement	FALSE	1	0.111
346	YPL184C	Agreement	TRUE	1	0.192
347	YHR122W	Agreement	TRUE	0	0.004
348	YKL086W	No agreement	FALSE	1	0.29
349	YIL119C	Agreement	FALSE	1	0.076
350	YJR133W	No agreement	TRUE	1	0
351	YDR443C	Agreement	TRUE	1	0
352	YGR250C	No agreement	TRUE	1	0
353	YJL158C	No agreement	FALSE	1	0
354	YOR273C	Agreement	TRUE	1	0
355	YMR126C	Agreement	FALSE	1	0.133
356	YLR460C	Agreement	TRUE	1	0.021
357	YMR206W	Agreement	TRUE	1	0
358	YLR370C	Agreement	TRUE	1	0
359	YGL205W	Agreement	TRUE	1	0
360	YKL043W	Agreement	TRUE	1	0.016
361	YGR203W	No agreement	TRUE	1	0
362	YDR255C	Agreement	TRUE	1	0
363	YGR049W	No agreement	TRUE	1	0.004
364	YDR516C	No agreement	FALSE	1	0
365	YDL085W	No agreement	TRUE	1	0
366	YMR062C	No agreement	TRUE	1	0
367	YDR350C	Agreement	TRUE	1	0.002
368	YFL048C	Agreement	FALSE	1	0.037
369	YBR185C	No agreement	TRUE	1	0
370	YOL060C	Agreement	TRUE	1	0
371	YBL032W	No agreement	FALSE	1	0
372	YMR022W	Agreement	FALSE	1	0.111
373	YDR056C	No agreement	FALSE	1	0.219
374	YPL026C	No agreement	TRUE	1	0.002
375	YPL006W	Agreement	FALSE	1	0
376	YDR536W	Agreement	FALSE	1	0
377	YJL083W	Agreement	TRUE	1	0
378	YER093C-A	Agreement	TRUE	1	0
379	YOR230W	Agreement	FALSE	1	0
380	YHL040C	No agreement	FALSE	1	0
381	YMR189W	Agreement	FALSE	1	0.001
382	YDL194W	Agreement	TRUE	1	0
383	YMR168C	No agreement	FALSE	0	0.263
384	YFR025C	Agreement	TRUE	1	0
385	YOR250C	No agreement	FALSE	0	0.055
386	YGR232W	No agreement	FALSE	1	0.029
387	YCL040W	Agreement	FALSE	1	0

388	YGR177C	Agreement	FALSE	1	0
389	YGR201C	Agreement	FALSE	1	0.013
390	YKL217W	No agreement	FALSE	1	0
391	YJL082W	Agreement	TRUE	1	0.118
392	YNR034W	No agreement	TRUE	1	0
393	YPR065W	No agreement	TRUE	1	0.045
394	YOR100C	No agreement	TRUE	1	0
395	YGL009C	Agreement	TRUE	1	0
396	YMR298W	No agreement	TRUE	0	0
397	YHR109W	No agreement	TRUE	1	0
398	YDR341C	Agreement	TRUE	0	0
399	YEL039C	Agreement	TRUE	1	0
400	YGR121C	Agreement	FALSE	1	0.016
401	YGL096W	Agreement	TRUE	1	0
402	YPL031C	Agreement	TRUE	1	0.175
403	YKL091C	Agreement	TRUE	1	0
404	YJL089W	No agreement	TRUE	1	0
405	YCL057W	Agreement	TRUE	1	0.032
406	YDL079C	Agreement	TRUE	1	0.001
407	YMR217W	Agreement	FALSE	1	0.018
408	YDR354W	No agreement	TRUE	1	0.01
409	YPL264C	No agreement	FALSE	1	0
410	YIL087C	Agreement	TRUE	1	0
411	YPL111W	No agreement	FALSE	1	0.119
412	YHR183W	No agreement	FALSE	1	0
413	YDR119W	Agreement	FALSE	1	0.287
414	YNL142W	Agreement	FALSE	1	0.01
415	YLR258W	Agreement	FALSE	1	0
416	YDR003W	Agreement	TRUE	1	0
417	YHR208W	Agreement	FALSE	0	0
418	YBR092C	No agreement	FALSE	1	0.015
419	YCR010C	No agreement	FALSE	1	0
420	YDR436W	Agreement	TRUE	1	0
421	YNR002C	No agreement	FALSE	1	0
422	YCL049C	No agreement	FALSE	1	0
423	YOR303W	No agreement	FALSE	1	0
424	YFL054C	Agreement	TRUE	1	0.15
425	YAR023C	Agreement	TRUE	1	0.032
426	YLR058C	Agreement	FALSE	1	0
427	YNL160W	Agreement	FALSE	1	0
428	YPL106C	No agreement	TRUE	1	0
429	YHR017W	Agreement	TRUE	1	0.033
430	YKR050W	Agreement	TRUE	1	0
431	YBR269C	Agreement	TRUE	1	0
432	YIL033C	Agreement	FALSE	1	0
433	YEL011W	Agreement	TRUE	1	0
434	YGL148W	No agreement	TRUE	1	0
435	YPR084W	No agreement	FALSE	1	0.066
436	YHR053C	Agreement	TRUE	0	0.046

437	YNR036C	No agreement	FALSE	1	0
438	YER010C	Agreement	TRUE	1	0
439	YNL294C	No agreement	FALSE	1	0.204
440	YMR011W	Agreement	TRUE	1	0
441	YHR049W	No agreement	TRUE	1	0.03
442	YLR432W	Agreement	FALSE	1	0
443	YHL028W	No agreement	TRUE	1	0
444	YPL098C	No agreement	TRUE	1	0
445	YIL116W	Agreement	TRUE	1	0.077
446	YJL103C	Agreement	FALSE	0	0.277
447	YIR036C	Agreement	FALSE	1	0
448	YLR149C	Agreement	TRUE	1	0
449	YDR347W	No agreement	TRUE	1	0
450	YKL209C	Agreement	TRUE	1	0.008
451	YMR238W	No agreement	TRUE	1	0.025
452	YDR513W	Agreement	TRUE	1	0
453	YMR291W	Agreement	FALSE	1	0
454	YBR223C	Agreement	TRUE	1	0.007
455	YIR022W	Agreement	FALSE	0	0.069
456	YFL010C	Agreement	FALSE	0	0
457	YPR199C	Agreement	TRUE	1	0
458	YFL044C	Agreement	TRUE	1	0.031
459	YOR150W	No agreement	TRUE	1	0
460	YIL083C	Agreement	TRUE	0	0.014
461	YER134C	No agreement	TRUE	1	0.001
462	YPL004C	Agreement	TRUE	1	0
463	YMR157C	No agreement	TRUE	1	0
464	YBR126C	Agreement	TRUE	1	0
465	YOR363C	Agreement	TRUE	1	0
466	YOR136W	Agreement	FALSE	1	0
467	YIL160C	Agreement	TRUE	1	0
468	YML087C	No agreement	TRUE	1	0
469	YJR109C	No agreement	TRUE	1	0
470	YDL029W	Agreement	TRUE	0	0
471	YPR098C	Agreement	FALSE	1	0
472	YHL021C	Agreement	TRUE	1	0
473	YPR109W	Agreement	TRUE	1	0
474	YOL008W	No agreement	FALSE	1	0.256
475	YDR418W	Agreement	FALSE	1	0
476	YPL059W	No agreement	TRUE	1	0.005
477	YDR304C	No agreement	TRUE	1	0.251
478	YBR299W	Agreement	TRUE	0	0
479	YJL026W	Agreement	FALSE	0	0
480	YPL159C	No agreement	TRUE	1	0.002
481	YEL020C	No agreement	FALSE	1	0
482	YDL049C	Agreement	TRUE	1	0
483	YDL238C	Agreement	TRUE	1	0
484	YGL115W	No agreement	TRUE	1	0
485	YJL217W	No agreement	FALSE	1	0

486	YHR171W	Agreement	TRUE	1	0
487	YLR180W	Agreement	FALSE	1	0
488	YPL170W	No agreement	TRUE	1	0
489	YER069W	No agreement	TRUE	1	0
490	YPR184W	Agreement	FALSE	1	0
491	YIL009C-A	No agreement	TRUE	1	0.014
492	YER051W	Agreement	TRUE	1	0.037
493	YPR172W	Agreement	TRUE	1	0
494	YDR033W	No agreement	FALSE	1	0
495	YDL217C	No agreement	TRUE	0	0.002
496	YGR204W	Agreement	TRUE	1	0
497	YLR303W	Agreement	FALSE	1	0.055
498	YGL234W	Agreement	FALSE	1	0
499	YDL053C	Agreement	TRUE	1	0.03
500	YHR018C	No agreement	TRUE	1	0.004
501	YIL165C	Agreement	TRUE	1	0.027
502	YOR192C	No agreement	TRUE	1	0.012
503	YBR006W	Agreement	TRUE	0	0
504	YHR179W	No agreement	TRUE	1	0
505	YMR315W	Agreement	TRUE	1	0
506	YDR368W	Agreement	TRUE	1	0
507	YOL159C	No agreement	TRUE	1	0.21
508	YPL214C	No agreement	TRUE	1	0
509	YNL241C	No agreement	FALSE	1	0.246
510	YJR058C	No agreement	TRUE	1	0.197
511	YNL200C	Agreement	FALSE	1	0
512	YCL030C	Agreement	TRUE	1	0
513	YOR380W	Agreement	FALSE	1	0
514	YJL116C	No agreement	FALSE	1	0.071
515	YKL140W	Agreement	TRUE	1	0
516	YKL065C	Agreement	TRUE	1	0
517	YLR290C	Agreement	TRUE	1	0
518	YBR293W	Agreement	FALSE	1	0.012
519	YNL116W	Agreement	TRUE	1	0
520	YIL162W	No agreement	TRUE	1	0
521	YPL095C	Agreement	TRUE	1	0.001
522	YNL185C	No agreement	TRUE	1	0
523	YGL006W	Agreement	TRUE	1	0
524	YLR390W	No agreement	FALSE	1	0
525	YDR479C	Agreement	TRUE	1	0
526	YJR033C	Agreement	TRUE	1	0
527	YML001W	Agreement	TRUE	1	0.048
528	YER065C	Agreement	TRUE	1	0
529	YNL026W	No agreement	TRUE	0	0
530	YDR294C	Agreement	TRUE	1	0
531	YNL202W	Agreement	TRUE	1	0
532	YOR108W	Agreement	TRUE	1	0.014
533	YML054C	Agreement	TRUE	1	0
534	YGL047W	Agreement	TRUE	0	0

535	YDR160W	No agreement	TRUE	0	0.161
536	YLR059C	No agreement	FALSE	1	0.089
537	YPL203W	Agreement	TRUE	1	0
538	YCL039W	Agreement	TRUE	1	0
539	YBR050C	No agreement	FALSE	1	0.275
540	YPL135W	No agreement	FALSE	1	0
541	YDL222C	Agreement	TRUE	1	0
542	YMR081C	Agreement	TRUE	1	0.001
543	YDR362C	No agreement	TRUE	0	0.008
544	YDL170W	No agreement	TRUE	1	0.001
545	YKL094W	Agreement	TRUE	1	0.002
546	YIL062C	Agreement	TRUE	0	0.064
547	YNL117W	No agreement	TRUE	1	0.014
548	YJR118C	No agreement	TRUE	1	0.017
549	YIL157C	No agreement	TRUE	1	0
550	YGR234W	No agreement	TRUE	1	0
551	YER174C	No agreement	FALSE	1	0
552	YMR042W	No agreement	FALSE	1	0.035
553	YGL019W	No agreement	TRUE	1	0.059
554	YML131W	Agreement	TRUE	1	0
555	YAL060W	No agreement	TRUE	1	0.001
556	YEL041W	Agreement	TRUE	1	0
557	YDL174C	Agreement	TRUE	1	0
558	YGR184C	No agreement	FALSE	1	0.272
559	YJL100W	Agreement	FALSE	1	0.048
560	YHR008C	Agreement	TRUE	1	0
561	YLL024C	No agreement	TRUE	1	0.006
562	YKR039W	No agreement	FALSE	1	0.001
563	YAL067C	No agreement	FALSE	1	0.287
564	YJR111C	No agreement	TRUE	1	0.026
565	YIL041W	Agreement	TRUE	1	0.011
566	YDL033C	No agreement	TRUE	1	0
567	YDL045C	Agreement	TRUE	0	0
568	YHR033W	No agreement	FALSE	1	0.043
569	YHL024W	Agreement	TRUE	1	0
570	YGL068W	No agreement	TRUE	0	0
571	YMR279C	Agreement	TRUE	1	0
572	YGR253C	No agreement	FALSE	0	0.204
573	YNL330C	No agreement	TRUE	1	0
574	YIL027C	No agreement	FALSE	1	0.212
575	YIL164C	No agreement	TRUE	1	0
576	YOL058W	No agreement	TRUE	1	0
577	YDR265W	Agreement	TRUE	1	0.006
578	YPL226W	Agreement	FALSE	1	0.022
579	YPL109C	Agreement	TRUE	1	0
580	YFL004W	No agreement	TRUE	0	0.002
581	YLR199C	No agreement	TRUE	1	0.14
582	YIL007C	Agreement	TRUE	1	0.191
583	YLR250W	Agreement	TRUE	1	0.004

584	YJR085C	Agreement	FALSE	0	0.001
585	YCR021C	Agreement	TRUE	1	0
586	YOL126C	No agreement	TRUE	1	0
587	YLR174W	No agreement	TRUE	1	0
588	YOL065C	Agreement	TRUE	1	0
589	YER021W	Agreement	TRUE	0	0.002
590	YIL166C	No agreement	FALSE	1	0
591	YDL230W	Agreement	TRUE	1	0.001
592	YMR220W	Agreement	FALSE	0	0.198
593	YGR010W	Agreement	FALSE	1	0.001
594	YLL056C	No agreement	TRUE	1	0
595	YOR035C	Agreement	TRUE	1	0
596	YLR219W	Agreement	TRUE	1	0
597	YGR209C	Agreement	TRUE	1	0.049
598	YER015W	Agreement	TRUE	0	0
599	YDL182W	Agreement	TRUE	1	0
600	YIL124W	Agreement	FALSE	1	0
601	YDL181W	Agreement	TRUE	1	0
602	YDR394W	Agreement	TRUE	0	0.007
603	YER094C	Agreement	TRUE	0	0
604	YDR074W	Agreement	TRUE	1	0
605	YKL064W	Agreement	TRUE	1	0.009
606	YPL201C	Agreement	TRUE	1	0
607	YOR323C	No agreement	TRUE	1	0
608	YDR533C	Agreement	TRUE	1	0
609	YJR101W	No agreement	FALSE	0	0
610	YKL013C	Agreement	TRUE	0	0.037
611	YKL162C	Agreement	TRUE	1	0
612	YMR237W	Agreement	TRUE	1	0
613	YJL210W	No agreement	FALSE	1	0
614	YLR386W	No agreement	FALSE	1	0.162
615	YNR018W	No agreement	TRUE	1	0.001
616	YGL180W	No agreement	TRUE	1	0
617	YOR031W	Agreement	TRUE	1	0
618	YKL098W	No agreement	FALSE	1	0.143
619	YDR132C	Agreement	FALSE	1	0.178
620	YDR261C	No agreement	TRUE	1	0
621	YLR346C	No agreement	TRUE	1	0
622	YPL163C	No agreement	FALSE	1	0
623	YMR139W	Agreement	TRUE	1	0
624	YPL016W	Agreement	TRUE	0	0
625	YGR185C	Agreement	FALSE	0	0.198
626	YKR075C	Agreement	TRUE	1	0
627	YDR034W-B	Agreement	TRUE	0	0
628	YBL047C	Agreement	TRUE	1	0
629	YBR021W	Agreement	FALSE	1	0.004
630	YPR021C	No agreement	TRUE	0	0
631	YOR231W	Agreement	FALSE	1	0.034
632	YOR377W	Agreement	FALSE	1	0.016

633	YKL171W	Agreement	TRUE	1	0
634	YIL005W	No agreement	FALSE	1	0.061
635	YPR030W	Agreement	FALSE	1	0.205
636	YOR227W	Agreement	TRUE	1	0
637	YHR083W	Agreement	TRUE	0	0.027
638	YHR027C	Agreement	FALSE	0	0.217
639	YKL026C	Agreement	TRUE	1	0
640	YGR194C	Agreement	TRUE	1	0
641	YKR089C	Agreement	TRUE	1	0
642	YNL131W	Agreement	TRUE	0	0
643	YBL029W	No agreement	TRUE	1	0.002
644	YER045C	No agreement	TRUE	1	0.091
645	YPL171C	Agreement	TRUE	1	0
646	YJR025C	No agreement	FALSE	1	0
647	YKL126W	Agreement	TRUE	1	0.003
648	YJL190C	Agreement	FALSE	1	0
649	YFR015C	Agreement	TRUE	1	0
650	YFR052W	Agreement	TRUE	0	0.05
651	YBR037C	No agreement	TRUE	1	0
652	YMR059W	No agreement	FALSE	0	0.003
653	YBR053C	Agreement	TRUE	1	0
654	YML050W	No agreement	TRUE	1	0
655	YKL106W	No agreement	TRUE	1	0.017
656	YGR141W	Agreement	FALSE	1	0.137
657	YDL120W	Agreement	TRUE	0	0
658	YPR005C	Agreement	TRUE	1	0.009
659	YER186C	No agreement	TRUE	1	0.039
660	YDR144C	Agreement	FALSE	1	0
661	YDR511W	No agreement	TRUE	1	0
662	YBL067C	Agreement	TRUE	1	0
663	YCL016C	No agreement	FALSE	1	0.001
664	YER024W	No agreement	TRUE	1	0
665	YBL050W	Agreement	FALSE	0	0.063
666	YDL078C	Agreement	TRUE	1	0
667	YIL097W	Agreement	TRUE	1	0.001
668	YBR117C	Agreement	TRUE	1	0
669	YPR001W	No agreement	TRUE	1	0
670	YPL215W	No agreement	TRUE	1	0
671	YDR259C	No agreement	FALSE	1	0.138
672	YIL055C	Agreement	TRUE	1	0
673	YPR178W	No agreement	FALSE	0	0.275
674	YGL011C	Agreement	FALSE	0	0.106
675	YBR035C	No agreement	FALSE	1	0.002
676	YKL087C	No agreement	TRUE	1	0
677	YMR158W	No agreement	TRUE	1	0
678	YMR105C	Agreement	TRUE	1	0
679	YKL211C	No agreement	FALSE	1	0.252
680	YKL121W	Agreement	TRUE	1	0.001
681	YGR254W	Agreement	FALSE	0	0.078

682	YEL057C	Agreement	TRUE	1	0
683	YHR209W	Agreement	TRUE	1	0
684	YLL015W	Agreement	TRUE	1	0
685	YMR264W	Agreement	TRUE	1	0
686	YOR023C	No agreement	FALSE	1	0.147
687	YBR068C	No agreement	TRUE	1	0
688	YGL166W	No agreement	FALSE	1	0.191
689	YDR286C	Agreement	TRUE	1	0
690	YOR291W	Agreement	FALSE	1	0.018
691	YLR087C	Agreement	TRUE	1	0.065
692	YDR214W	No agreement	TRUE	1	0
693	YKL067W	No agreement	TRUE	1	0
694	YML103C	No agreement	FALSE	1	0.201
695	YPR151C	Agreement	FALSE	0	0
696	YLR371W	Agreement	FALSE	1	0
697	YLL042C	No agreement	FALSE	1	0.068
698	YLR207W	Agreement	FALSE	1	0.052
699	YIR021W	No agreement	TRUE	1	0
700	YBR151W	Agreement	TRUE	1	0.019
701	YKL150W	Agreement	TRUE	1	0
702	YFR045W	No agreement	TRUE	1	0
703	YPL161C	Agreement	FALSE	1	0.008
704	YLR312C	Agreement	TRUE	1	0
705	YDR221W	Agreement	TRUE	1	0.05
706	YDR336W	Agreement	TRUE	1	0.078
707	YKL016C	Agreement	TRUE	1	0
708	YJR095W	No agreement	TRUE	0	0
709	YDL014W	Agreement	FALSE	0	0.005
710	YCR020C	No agreement	TRUE	1	0
711	YOR317W	No agreement	FALSE	1	0
712	YBL007C	Agreement	FALSE	1	0
713	YJR099W	Agreement	TRUE	1	0
714	YOR258W	No agreement	TRUE	1	0.041
715	YGL091C	No agreement	FALSE	0	0.255
716	YBR294W	Agreement	TRUE	1	0
717	YLR163C	No agreement	FALSE	0	0
718	YHR147C	No agreement	TRUE	1	0
719	YDR078C	Agreement	FALSE	1	0.202
720	YHR200W	Agreement	FALSE	1	0.238
721	YOR052C	No agreement	FALSE	1	0.001
722	YPR155C	Agreement	FALSE	1	0
723	YDL027C	Agreement	TRUE	1	0
724	YIR038C	Agreement	TRUE	1	0
725	YBR137W	Agreement	FALSE	1	0
726	YKR018C	Agreement	TRUE	1	0.001
727	YGL124C	No agreement	FALSE	1	0.231
728	YGR019W	Agreement	TRUE	1	0.001
729	YER067W	Agreement	TRUE	1	0
730	YGR028W	No agreement	FALSE	1	0

731	YLR060W	Agreement	TRUE	0	0.007
732	YOR220W	Agreement	TRUE	1	0
733	YLR351C	No agreement	FALSE	1	0
734	YPL117C	No agreement	FALSE	0	0
735	YHL016C	Agreement	FALSE	1	0
736	YJR137C	Agreement	TRUE	1	0.003
737	YLL039C	Agreement	TRUE	1	0
738	YGL013C	No agreement	FALSE	1	0.007
739	YPR010C	Agreement	TRUE	0	0.019
740	YBL106C	Agreement	TRUE	1	0
741	YLR206W	Agreement	FALSE	1	0.002
742	YPR023C	Agreement	FALSE	1	0.265
743	YIL075C	Agreement	TRUE	0	0.103
744	YER046W	No agreement	FALSE	1	0.062
745	YOR025W	Agreement	TRUE	1	0
746	YLR098C	No agreement	TRUE	1	0.248
747	YML056C	Agreement	TRUE	1	0.048
748	YDR204W	Agreement	TRUE	1	0
749	YPL133C	No agreement	FALSE	1	0.004
750	YMR180C	Agreement	FALSE	1	0
751	YNL070W	No agreement	TRUE	1	0
752	YOR130C	Agreement	TRUE	1	0
753	YER062C	No agreement	TRUE	1	0.097
754	YER012W	Agreement	FALSE	0	0.015
755	YNL100W	Agreement	FALSE	1	0
756	YCL025C	No agreement	TRUE	1	0.004
757	YGL224C	Agreement	TRUE	1	0
758	YJR039W	Agreement	TRUE	0	0
759	YDR237W	No agreement	TRUE	1	0
760	YER064C	No agreement	TRUE	1	0
761	YMR163C	Agreement	FALSE	1	0.019
762	YML125C	No agreement	FALSE	0	0.003
763	YNL278W	Agreement	TRUE	1	0
764	YDR322W	No agreement	TRUE	1	0
765	YLR216C	No agreement	FALSE	1	0
766	YER101C	Agreement	TRUE	1	0
767	YKR067W	Agreement	FALSE	1	0
768	YNL115C	Agreement	TRUE	1	0
769	YKR091W	Agreement	TRUE	1	0
770	YDL204W	Agreement	TRUE	1	0
771	YBL058W	Agreement	FALSE	1	0.001
772	YPL156C	No agreement	TRUE	1	0.011
773	YGL117W	No agreement	TRUE	1	0.019
774	YBR173C	Agreement	TRUE	1	0.05
775	YFR017C	Agreement	TRUE	1	0
776	YOL140W	No agreement	TRUE	0	0
777	YOL043C	No agreement	FALSE	1	0.082
778	YKL029C	Agreement	TRUE	1	0.086
779	YJR052W	No agreement	FALSE	1	0

780	YMR016C	No agreement	FALSE	1	0
781	YGR067C	No agreement	TRUE	1	0.001
782	YGR088W	Agreement	TRUE	1	0
783	YLL007C	Agreement	FALSE	1	0
784	YCR071C	No agreement	FALSE	1	0
785	YNL217W	No agreement	TRUE	1	0.001
786	YMR175W	No agreement	FALSE	1	0
787	YKL125W	Agreement	FALSE	0	0.23
788	YFL014W	Agreement	FALSE	1	0
789	YKR081C	Agreement	FALSE	0	0.07
790	YKR077W	Agreement	FALSE	1	0.007
791	YLR103C	Agreement	TRUE	0	0
792	YDR046C	Agreement	FALSE	1	0.006
793	YJL181W	Agreement	FALSE	1	0
794	YJL148W	Agreement	FALSE	1	0.019
795	YLR274W	Agreement	TRUE	0	0.001
796	YKL172W	Agreement	TRUE	0	0.063
797	YMR194W	Agreement	FALSE	1	0
798	YPR169W	Agreement	FALSE	0	0.122
799	YKR049C	Agreement	FALSE	1	0
800	YJL122W	Agreement	FALSE	1	0.039
801	YMR128W	Agreement	FALSE	0	0.037
802	YLR196W	Agreement	FALSE	0	0.066
803	YGR280C	Agreement	FALSE	0	0.125
804	YNL175C	Agreement	FALSE	1	0.035
805	YDL223C	Agreement	TRUE	1	0
806	YLR129W	Agreement	FALSE	0	0.09
807	YNR053C	Agreement	FALSE	0	0.034
808	YBR247C	Agreement	FALSE	0	0.121
809	YLR197W	Agreement	FALSE	0	0.007
810	YHR066W	Agreement	FALSE	1	0.053
811	YOR091W	Agreement	TRUE	1	0.025
812	YAL062W	No agreement	FALSE	1	0.053
813	YIL046W	Agreement	FALSE	0	0.246
814	YLL060C	No agreement	FALSE	1	0
815	YNL103W	No agreement	FALSE	0	0.001
816	YOR144C	Agreement	TRUE	1	0
817	YDL060W	Agreement	FALSE	0	0.047
818	YDR528W	Agreement	FALSE	1	0
819	YJR097W	Agreement	FALSE	1	0.024
820	YML082W	Agreement	FALSE	1	0.015
821	YLR222C	Agreement	FALSE	0	0.039
822	YMR093W	Agreement	FALSE	0	0.03
823	YDL164C	Agreement	TRUE	0	0
824	YOR033C	Agreement	TRUE	1	0
825	YIR017C	Agreement	TRUE	1	0.04
826	YBR079C	Agreement	FALSE	0	0.007
827	YAL059W	Agreement	FALSE	1	0.068
828	YBL043W	No agreement	TRUE	1	0

829	YNL277W	Agreement	TRUE	1	0
830	YLR409C	Agreement	TRUE	0	0.044
831	YLL034C	Agreement	FALSE	0	0.06
832	YGL120C	Agreement	TRUE	0	0.091
833	YGR145W	Agreement	FALSE	0	0.067
834	YMR195W	Agreement	TRUE	1	0.001
835	YJL025W	Agreement	TRUE	0	0.113
836	YBL035C	Agreement	TRUE	0	0
837	YJL110C	Agreement	TRUE	1	0.006
838	YNL062C	Agreement	FALSE	0	0.038
839	YBR213W	Agreement	TRUE	1	0
840	YKL009W	Agreement	FALSE	1	0.049
841	YGR211W	Agreement	TRUE	0	0
842	YPR167C	Agreement	TRUE	1	0
843	YOR243C	Agreement	FALSE	1	0.014
844	YNL248C	Agreement	TRUE	1	0.067
845	YPL266W	Agreement	TRUE	0	0.056
846	YMR131C	Agreement	FALSE	0	0.058
847	YPR035W	No agreement	TRUE	0	0.034
848	YML030W	No agreement	FALSE	1	0
849	YKR079C	Agreement	FALSE	0	0.027
850	YOL123W	Agreement	FALSE	0	0.003
851	YOR272W	Agreement	TRUE	0	0.042
852	YPL212C	Agreement	FALSE	1	0.032
853	YKL045W	Agreement	TRUE	0	0
854	YPL254W	Agreement	FALSE	1	0.152
855	YGR258C	Agreement	TRUE	0	0.015
856	YER030W	Agreement	TRUE	1	0
857	YMR174C	Agreement	FALSE	1	0
858	YMR219W	Agreement	TRUE	1	0.031
859	YPL245W	Agreement	TRUE	1	0.002
860	YKR036C	No agreement	TRUE	1	0.001
861	YHR169W	Agreement	FALSE	0	0.098
862	YBR104W	Agreement	FALSE	1	0.02
863	YKR092C	Agreement	TRUE	1	0.169
864	YKL001C	Agreement	TRUE	1	0
865	YGR021W	No agreement	TRUE	1	0
866	YPR158W	No agreement	TRUE	1	0
867	YJR155W	Agreement	FALSE	0	0
868	YKR025W	No agreement	TRUE	0	0.132
869	YCL033C	Agreement	FALSE	1	0
870	YOR054C	Agreement	TRUE	1	0
871	YIL136W	Agreement	TRUE	0	0
872	YNR071C	Agreement	TRUE	1	0.159
873	YKL151C	Agreement	FALSE	1	0
874	YBL039C	Agreement	TRUE	1	0.096
875	YHR176W	Agreement	FALSE	1	0
876	YBR208C	No agreement	TRUE	1	0.019
877	YKL137W	No agreement	TRUE	1	0

878	YLL009C	No agreement	TRUE	1	0
879	YER058W	No agreement	TRUE	1	0
880	YOL010W	Agreement	FALSE	0	0.022
881	YML128C	Agreement	FALSE	1	0
882	YOR358W	No agreement	TRUE	1	0.036
883	YMR049C	Agreement	TRUE	0	0.032
884	YNL077W	No agreement	FALSE	1	0
885	YNL312W	Agreement	TRUE	0	0.013
886	YMR089C	No agreement	TRUE	1	0
887	YNL274C	Agreement	FALSE	1	0
888	YNL173C	Agreement	TRUE	1	0
889	YER133W	Agreement	TRUE	0	0.055
890	YGL227W	Agreement	TRUE	1	0
891	YGR005C	No agreement	TRUE	0	0
892	YKL195W	No agreement	TRUE	0	0
893	YJL066C	Agreement	FALSE	1	0
894	YKR035W-A	No agreement	TRUE	0	0
895	YJL164C	Agreement	FALSE	1	0
896	YKR016W	No agreement	TRUE	1	0
897	YER159C	Agreement	TRUE	0	0
898	YLR330W	Agreement	TRUE	1	0
899	YNL015W	Agreement	TRUE	1	0
900	YER182W	No agreement	FALSE	1	0
901	YIR003W	Agreement	TRUE	1	0.001
902	YPL013C	No agreement	TRUE	1	0
903	YNL252C	No agreement	TRUE	1	0
904	YOR215C	Agreement	FALSE	1	0
905	YER020W	Agreement	FALSE	1	0.168
906	YKL054C	No agreement	TRUE	1	0.022
907	YOR158W	No agreement	TRUE	1	0
908	YPR148C	Agreement	TRUE	1	0
909	YEL066W	No agreement	FALSE	1	0.181
910	YDL173W	No agreement	TRUE	1	0.024
911	YDR168W	Agreement	TRUE	0	0.002
912	YLR053C	Agreement	TRUE	1	0.002
913	YNL250W	No agreement	TRUE	1	0.001
914	YDL239C	Agreement	TRUE	1	0
915	YDR357C	Agreement	FALSE	1	0
916	YBL022C	No agreement	TRUE	1	0.003
917	YHR197W	Agreement	TRUE	0	0.074
918	YDR316W	No agreement	TRUE	1	0
919	YJL049W	Agreement	FALSE	1	0.181
920	YPL173W	No agreement	TRUE	1	0
921	YLR257W	No agreement	TRUE	1	0.054
922	YOR289W	Agreement	FALSE	1	0
923	YDR405W	Agreement	TRUE	1	0
924	YPR047W	No agreement	TRUE	1	0
925	YOR290C	Agreement	FALSE	1	0.016
926	YGR130C	Agreement	TRUE	1	0

927	YOR075W	No agreement	FALSE	0	0.092
928	YBR248C	Agreement	FALSE	1	0.018
929	YFR003C	Agreement	TRUE	0	0
930	YOR341W	Agreement	TRUE	0	0.013
931	YBL064C	Agreement	TRUE	1	0
932	YJL165C	Agreement	FALSE	1	0.276
933	YLR092W	Agreement	TRUE	1	0
934	YOR042W	Agreement	TRUE	1	0.002
935	YJL112W	No agreement	TRUE	1	0
936	YKL142W	Agreement	FALSE	1	0
937	YPL140C	No agreement	FALSE	1	0.102
938	YGR161C	No agreement	TRUE	1	0
939	YLR254C	Agreement	TRUE	1	0
940	YOR097C	Agreement	TRUE	1	0.012
941	YDR068W	No agreement	TRUE	1	0.001
942	YJL088W	No agreement	TRUE	1	0
943	YKL216W	Agreement	TRUE	1	0.015
944	YOR128C	Agreement	TRUE	1	0.001
945	YLR247C	Agreement	FALSE	1	0.156
946	YJL080C	Agreement	FALSE	1	0
947	YKR076W	Agreement	FALSE	1	0
948	YMR301C	Agreement	TRUE	0	0
949	YGR239C	Agreement	TRUE	1	0
950	YML018C	Agreement	TRUE	1	0.017
951	YNL064C	No agreement	FALSE	1	0
952	YNR049C	Agreement	FALSE	1	0.215
953	YHL036W	Agreement	TRUE	1	0.019
954	YLR069C	No agreement	TRUE	1	0
955	YMR322C	Agreement	FALSE	1	0
956	YMR173W	Agreement	FALSE	1	0
957	YNL289W	Agreement	TRUE	1	0
958	YHL023C	Agreement	TRUE	1	0.218
959	YDL007W	Agreement	TRUE	0	0.007
960	YJL140W	Agreement	TRUE	1	0.076
961	YJL136C	Agreement	TRUE	1	0.003
962	YGL184C	Agreement	TRUE	0	0
963	YFR031C	No agreement	TRUE	0	0
964	YOR125C	Agreement	FALSE	1	0.001
965	YBL021C	No agreement	TRUE	1	0
966	YKR099W	No agreement	FALSE	1	0.027
967	YDR282C	No agreement	FALSE	1	0
968	YGR008C	Agreement	FALSE	1	0
969	YHL019C	Agreement	FALSE	1	0.146
970	YER087W	No agreement	FALSE	1	0
971	YKL002W	Agreement	FALSE	1	0.185
972	YGR055W	Agreement	TRUE	1	0
973	YOR257W	Agreement	TRUE	0	0
974	YBR251W	No agreement	TRUE	1	0
975	YGR231C	No agreement	FALSE	1	0.001

976	YER115C	Agreement	FALSE	1	0
977	YBR268W	No agreement	FALSE	1	0
978	YER068W	Agreement	TRUE	1	0.033
979	YER050C	No agreement	TRUE	1	0
980	YLR165C	Agreement	TRUE	1	0.034
981	YJR063W	Agreement	TRUE	1	0.053
982	YJL063C	No agreement	TRUE	1	0
983	YBR163W	Agreement	FALSE	1	0.192
984	YML079W	Agreement	FALSE	1	0.009
985	YKL105C	Agreement	TRUE	1	0.003
986	YBR282W	No agreement	TRUE	1	0
987	YOL082W	Agreement	FALSE	1	0
988	YOR027W	No agreement	TRUE	1	0
989	YOL023W	No agreement	TRUE	1	0
990	YDR358W	Agreement	FALSE	1	0
991	YPL008W	No agreement	FALSE	1	0.27
992	YMR302C	Agreement	TRUE	1	0
993	YPL020C	No agreement	FALSE	0	0.142
994	YML117W	Agreement	TRUE	1	0.018
995	YBR033W	No agreement	TRUE	1	0
996	YJL055W	Agreement	FALSE	1	0.064
997	YJR080C	No agreement	TRUE	1	0
998	YOR207C	Agreement	TRUE	0	0.019
999	YER176W	Agreement	TRUE	1	0.065
1000	YMR207C	No agreement	TRUE	1	0.011
1001	YJR129C	No agreement	TRUE	1	0.002
1002	YDR070C	Agreement	TRUE	1	0
1003	YJR070C	Agreement	TRUE	1	0.02
1004	YMR040W	No agreement	FALSE	1	0.268
1005	YOL064C	Agreement	TRUE	1	0
1006	YDR337W	No agreement	FALSE	1	0
1007	YBR120C	No agreement	TRUE	1	0
1008	YLR107W	No agreement	TRUE	1	0.106
1009	YHR057C	Agreement	TRUE	1	0.002
1010	YDR022C	Agreement	FALSE	1	0
1011	YCR096C	No agreement	FALSE	0	0.284
1012	YIL093C	No agreement	TRUE	1	0
1013	YOL071W	Agreement	FALSE	1	0
1014	YIL068C	Agreement	TRUE	0	0
1015	YPL186C	Agreement	FALSE	1	0
1016	YLR021W	No agreement	TRUE	1	0.008
1017	YAL055W	Agreement	TRUE	1	0.047
1018	YCL035C	Agreement	TRUE	1	0
1019	YDR248C	No agreement	FALSE	1	0.068
1020	YER163C	No agreement	FALSE	1	0
1021	YJL070C	Agreement	FALSE	1	0.002
1022	YKL129C	Agreement	TRUE	1	0
1023	YDR226W	No agreement	TRUE	1	0
1024	YJL180C	No agreement	TRUE	1	0

1025	YCR083W	Agreement	FALSE	1	0
1026	YKR007W	No agreement	FALSE	1	0.219
1027	YBR024W	Agreement	FALSE	1	0.001
1028	YJL005W	Agreement	FALSE	0	0
1029	YCR003W	No agreement	TRUE	1	0
1030	YGR144W	No agreement	TRUE	1	0.284
1031	YGR155W	Agreement	TRUE	1	0
1032	YBR111C	Agreement	FALSE	1	0.072
1033	YDR330W	Agreement	TRUE	1	0
1034	YLR364W	Agreement	TRUE	1	0
1035	YDR181C	Agreement	TRUE	1	0
1036	YJR010W	Agreement	TRUE	1	0
1037	YNR037C	No agreement	FALSE	1	0
1038	YHR112C	Agreement	TRUE	1	0.167
1039	YKL103C	Agreement	FALSE	1	0
1040	YIR025W	No agreement	TRUE	1	0.046
1041	YDR423C	Agreement	FALSE	1	0.29
1042	YML076C	No agreement	FALSE	1	0.063
1043	YLR006C	Agreement	FALSE	1	0.001
1044	YER014W	No agreement	TRUE	1	0
1045	YLR049C	Agreement	TRUE	1	0.066
1046	YBR179C	Agreement	FALSE	1	0.118
1047	YGL156W	Agreement	FALSE	1	0
1048	YER162C	No agreement	FALSE	1	0.001
1049	YNL084C	Agreement	TRUE	1	0
1050	YDR416W	No agreement	FALSE	0	0.052
1051	YMR267W	No agreement	FALSE	1	0
1052	YKL168C	Agreement	FALSE	1	0
1053	YDR031W	No agreement	TRUE	1	0
1054	YDR277C	Agreement	FALSE	1	0.108
1055	YGR193C	Agreement	TRUE	1	0
1056	YMR300C	Agreement	TRUE	1	0.001
1057	YER169W	No agreement	FALSE	1	0.033
1058	YAL056W	Agreement	TRUE	1	0
1059	YPL222W	Agreement	FALSE	1	0
1060	YDR296W	No agreement	TRUE	1	0
1061	YPL167C	Agreement	FALSE	1	0
1062	YOL077C	Agreement	TRUE	0	0.026
1063	YLR454W	Agreement	FALSE	1	0
1064	YOL116W	Agreement	TRUE	1	0.174
1065	YLL027W	Agreement	FALSE	1	0.006
1066	YOR261C	Agreement	FALSE	0	0
1067	YOR185C	Agreement	TRUE	1	0
1068	YOL112W	No agreement	FALSE	1	0.009
1069	YHR146W	Agreement	TRUE	1	0
1070	YNL315C	No agreement	TRUE	1	0
1071	YPL118W	No agreement	TRUE	1	0
1072	YOL038W	Agreement	FALSE	0	0
1073	YFR053C	No agreement	TRUE	1	0

1074	YDR051C	No agreement	FALSE	1	0.154
1075	YMR107W	Agreement	TRUE	1	0
1076	YOR386W	Agreement	FALSE	1	0.024
1077	YIL098C	No agreement	FALSE	1	0
1078	YOR037W	No agreement	FALSE	1	0
1079	YKL167C	No agreement	FALSE	1	0
1080	YCR046C	No agreement	TRUE	1	0
1081	YLR270W	Agreement	FALSE	1	0
1082	YMR115W	Agreement	TRUE	1	0
1083	YLR312W-A	No agreement	TRUE	1	0
1084	YLR425W	Agreement	FALSE	1	0
1085	YDR466W	Agreement	FALSE	1	0
1086	YBR150C	Agreement	FALSE	1	0.191
1087	YOR173W	Agreement	TRUE	1	0
1088	YPL132W	No agreement	FALSE	1	0
1089	YNL295W	Agreement	TRUE	1	0
1090	YJL060W	Agreement	FALSE	1	0.278
1091	YPL240C	No agreement	FALSE	1	0
1092	YDR444W	No agreement	FALSE	0	0
1093	YLL001W	No agreement	TRUE	1	0.001
1094	YOL016C	Agreement	TRUE	1	0.002
1095	YGL121C	Agreement	TRUE	1	0
1096	YCR073C	Agreement	FALSE	1	0.012
1097	YHR192W	No agreement	FALSE	0	0.152
1098	YER071C	No agreement	FALSE	1	0.075
1099	YGL082W	Agreement	TRUE	1	0.007
1100	YIL074C	Agreement	TRUE	1	0
1101	YFR014C	Agreement	FALSE	1	0.04
1102	YIR024C	No agreement	TRUE	1	0.001
1103	YKL138C	No agreement	FALSE	1	0
1104	YDL110C	Agreement	TRUE	1	0
1105	YPR115W	Agreement	FALSE	1	0.004
1106	YBL033C	No agreement	TRUE	1	0.003
1107	YDR239C	Agreement	FALSE	1	0.001
1108	YOL096C	Agreement	FALSE	1	0
1109	YGR179C	No agreement	FALSE	0	0
1110	YOL097C	Agreement	TRUE	0	0.005
1111	YDL020C	No agreement	TRUE	1	0
1112	YIL107C	Agreement	FALSE	1	0
1113	YOR028C	Agreement	FALSE	1	0
1114	YDR305C	Agreement	TRUE	1	0
1115	YPL127C	No agreement	FALSE	1	0
1116	YOR196C	No agreement	TRUE	1	0.002
1117	YNR032C-A	No agreement	FALSE	1	0.001
1118	YAL039C	No agreement	FALSE	1	0.036
1119	YJL102W	No agreement	FALSE	1	0
1120	YER079W	Agreement	FALSE	1	0
1121	YKL053C-A	No agreement	FALSE	1	0
1122	YBL078C	Agreement	FALSE	1	0

1123	YLR448W	Agreement	TRUE	1	0
1124	YKR002W	Agreement	TRUE	0	0.001
1125	YOR036W	Agreement	FALSE	1	0
1126	YLR127C	Agreement	FALSE	0	0.157
1127	YDR452W	Agreement	FALSE	1	0
1128	YHR016C	Agreement	TRUE	1	0
1129	YIR032C	Agreement	FALSE	1	0.001
1130	YGL153W	Agreement	TRUE	1	0.001
1131	YLR327C	Agreement	FALSE	1	0
1132	YJR149W	Agreement	FALSE	1	0.001
1133	YBL041W	Agreement	FALSE	0	0.001
1134	YCR097W	Agreement	FALSE	0	0.001
1135	YOL083W	Agreement	TRUE	1	0
1136	YOR357C	Agreement	FALSE	1	0.145
1137	YKR085C	No agreement	FALSE	1	0
1138	YBL037W	No agreement	FALSE	1	0
1139	YAL012W	Agreement	TRUE	1	0
1140	YBL091C-A	Agreement	FALSE	1	0.009
1141	YPR160W	Agreement	TRUE	1	0
1142	YGL087C	Agreement	TRUE	1	0.024
1143	YGL233W	Agreement	FALSE	0	0.001
1144	YLL061W	No agreement	FALSE	1	0.141
1145	YHR152W	Agreement	FALSE	1	0
1146	YDL169C	Agreement	FALSE	1	0
1147	YGR047C	Agreement	FALSE	0	0.001
1148	YJL200C	Agreement	TRUE	1	0
1149	YGL134W	Agreement	TRUE	0	0.011
1150	YER073W	Agreement	TRUE	1	0.001
1151	YOR163W	Agreement	FALSE	1	0.012
1152	YMR097C	Agreement	FALSE	1	0.004
1153	YAR018C	Agreement	FALSE	1	0
1154	YJL003W	Agreement	FALSE	1	0.201
1155	YML007W	Agreement	FALSE	1	0
1156	YNL306W	No agreement	TRUE	0	0
1157	YDR397C	Agreement	FALSE	0	0.014
1158	YDR494W	No agreement	FALSE	1	0
1159	YER081W	Agreement	TRUE	1	0.009
1160	YKL196C	Agreement	FALSE	0	0.003
1161	YDR082W	No agreement	FALSE	0	0.092
1162	YER042W	No agreement	TRUE	1	0.009
1163	YHR012W	No agreement	FALSE	1	0.222
1164	YGL037C	Agreement	TRUE	1	0
1165	YIL017C	Agreement	FALSE	1	0
1166	YDR493W	No agreement	TRUE	0	0
1167	YEL012W	Agreement	FALSE	1	0
1168	YKL145W	Agreement	FALSE	0	0.001
1169	YDL121C	No agreement	FALSE	1	0.248
1170	YLR179C	Agreement	TRUE	1	0.001
1171	YBR062C	Agreement	FALSE	1	0

1172	YMR226C	Agreement	FALSE	1	0.054
1173	YFR011C	No agreement	FALSE	1	0
1174	YPL075W	No agreement	TRUE	0	0
1175	YPR166C	No agreement	FALSE	1	0
1176	YKL193C	Agreement	FALSE	0	0
1177	YDR061W	No agreement	FALSE	1	0
1178	YLR178C	Agreement	TRUE	1	0
1179	YDR308C	No agreement	FALSE	0	0.093
1180	YAL002W	Agreement	FALSE	1	0.1
1181	YDR122W	Agreement	FALSE	1	0
1182	YOR271C	Agreement	TRUE	1	0.034
1183	YDL021W	Agreement	TRUE	1	0.138
1184	YJL155C	Agreement	FALSE	1	0
1185	YJL035C	Agreement	TRUE	0	0.001
1186	YBR101C	No agreement	TRUE	1	0
1187	YPR086W	No agreement	FALSE	0	0.093
1188	YPL250C	No agreement	TRUE	1	0
1189	YER063W	Agreement	FALSE	1	0.001
1190	YJR100C	No agreement	FALSE	1	0
1191	YDR079W	No agreement	FALSE	1	0
1192	YNR035C	Agreement	FALSE	0	0
1193	YAL017W	Agreement	FALSE	1	0.001
1194	YJR142W	Agreement	FALSE	1	0
1195	YPR002W	No agreement	TRUE	1	0
1196	YGL228W	No agreement	FALSE	1	0.003
1197	YDR468C	No agreement	FALSE	0	0.071
1198	YLR061W	Agreement	TRUE	1	0
1199	YJL191W	Agreement	FALSE	1	0.007
1200	YMR060C	No agreement	TRUE	1	0
1201	YNL180C	Agreement	FALSE	1	0
1202	YKL170W	No agreement	FALSE	1	0
1203	YOR059C	Agreement	FALSE	1	0.167
1204	YCR094W	Agreement	FALSE	1	0.132
1205	YPR037C	Agreement	TRUE	1	0
1206	YDR268W	No agreement	FALSE	1	0
1207	YFL042C	Agreement	FALSE	1	0
1208	YDR150W	Agreement	FALSE	1	0
1209	YOR026W	Agreement	TRUE	1	0.003
1210	YBR094W	Agreement	TRUE	1	0
1211	YLR090W	No agreement	FALSE	1	0
1212	YMR314W	Agreement	FALSE	0	0.005
1213	YNL137C	No agreement	FALSE	0	0
1214	YDR175C	No agreement	TRUE	1	0
1215	YOR174W	No agreement	FALSE	0	0.003
1216	YFL033C	Agreement	TRUE	0	0.013
1217	YKR098C	Agreement	FALSE	1	0.08
1218	YMR027W	Agreement	FALSE	1	0.015
1219	YMR286W	No agreement	FALSE	1	0
1220	YGR244C	Agreement	FALSE	1	0

1221	YOL143C	Agreement	TRUE	1	0.002
1222	YNL108C	Agreement	FALSE	1	0.01
1223	YPR006C	No agreement	TRUE	1	0
1224	YGL095C	No agreement	FALSE	1	0.116
1225	YPR140W	No agreement	FALSE	1	0
1226	YDL147W	Agreement	FALSE	0	0
1227	YDL097C	Agreement	FALSE	0	0.002
1228	YOR095C	Agreement	TRUE	0	0.022
1229	YLR267W	No agreement	TRUE	1	0
1230	YKR001C	Agreement	FALSE	1	0.009
1231	YGL059W	No agreement	FALSE	1	0
1232	YMR199W	Agreement	TRUE	1	0
1233	YOR275C	No agreement	FALSE	1	0.012
1234	YDR486C	No agreement	FALSE	1	0.01
1235	YMR023C	No agreement	FALSE	1	0
1236	YMR100W	No agreement	FALSE	1	0
1237	YGR178C	No agreement	FALSE	1	0.214
1238	YKL159C	No agreement	TRUE	1	0
1239	YLR253W	No agreement	FALSE	1	0
1240	YMR090W	Agreement	TRUE	1	0
1241	YPR081C	Agreement	FALSE	0	0.174
1242	YER164W	No agreement	FALSE	1	0.077
1243	YOR018W	Agreement	FALSE	1	0
1244	YLL055W	Agreement	TRUE	1	0
1245	YFR030W	Agreement	TRUE	1	0
1246	YNL144C	Agreement	FALSE	1	0.103
1247	YEL073C	No agreement	FALSE	0	0
1248	YOR165W	No agreement	FALSE	1	0
1249	YNL242W	Agreement	FALSE	1	0.246
1250	YGL162W	Agreement	TRUE	1	0.044
1251	YFR004W	Agreement	FALSE	0	0.001
1252	YOL040C	Agreement	FALSE	0	0
1253	YOL055C	No agreement	FALSE	1	0
1254	YJR046W	No agreement	TRUE	0	0
1255	YPR008W	Agreement	FALSE	1	0
1256	YNL157W	No agreement	FALSE	1	0.025
1257	YJL042W	Agreement	FALSE	1	0.001
1258	YJR141W	No agreement	FALSE	0	0.106
1259	YGR132C	No agreement	TRUE	1	0
1260	YDR462W	No agreement	FALSE	1	0
1261	YLR204W	No agreement	FALSE	1	0
1262	YER055C	Agreement	TRUE	1	0
1263	YDR001C	Agreement	TRUE	1	0
1264	YIL057C	Agreement	FALSE	1	0
1265	YIL070C	No agreement	FALSE	1	0
1266	YDR391C	No agreement	FALSE	1	0.026
1267	YJL129C	No agreement	FALSE	1	0.212
1268	YDR219C	Agreement	FALSE	1	0.001
1269	YNR033W	Agreement	FALSE	1	0

1270	YML041C	No agreement	FALSE	1	0.287
1271	YPL265W	Agreement	TRUE	1	0.012
1272	YHR121W	No agreement	FALSE	1	0
1273	YDL070W	Agreement	FALSE	1	0
1274	YPL072W	No agreement	TRUE	1	0
1275	YKR074W	Agreement	FALSE	1	0.054
1276	YBR056W	Agreement	FALSE	1	0
1277	YPR103W	Agreement	FALSE	0	0
1278	YOR362C	Agreement	FALSE	0	0.001
1279	YBL059W	No agreement	FALSE	1	0
1280	YDL080C	No agreement	FALSE	1	0
1281	YHR071W	No agreement	TRUE	0	0
1282	YMR053C	Agreement	TRUE	1	0
1283	YGL048C	Agreement	FALSE	0	0.182
1284	YBR001C	Agreement	FALSE	1	0
1285	YOR329C	Agreement	FALSE	0	0
1286	YIL002C	No agreement	FALSE	1	0.049
1287	YGR171C	No agreement	FALSE	1	0
1288	YGL186C	Agreement	TRUE	0	0
1289	YGL219C	Agreement	FALSE	1	0.002
1290	YLR136C	No agreement	TRUE	1	0.044
1291	YHR087W	Agreement	TRUE	1	0
1292	YFR007W	No agreement	TRUE	1	0
1293	YBR204C	Agreement	FALSE	1	0.005
1294	YCR098C	No agreement	TRUE	1	0
1295	YNL281W	No agreement	FALSE	1	0
1296	YDR381W	No agreement	FALSE	0	0
1297	YMR021C	No agreement	FALSE	1	0.082
1298	YIR016W	Agreement	TRUE	1	0
1299	YGL027C	No agreement	FALSE	1	0
1300	YNR045W	Agreement	FALSE	1	0.001
1301	YIL077C	Agreement	FALSE	1	0
1302	YJL218W	No agreement	TRUE	1	0
1303	YJR008W	Agreement	FALSE	1	0
1304	YHR186C	No agreement	FALSE	0	0.004
1305	YLR348C	No agreement	FALSE	1	0
1306	YPL188W	No agreement	TRUE	1	0
1307	YDR063W	Agreement	TRUE	1	0
1308	YER143W	Agreement	FALSE	1	0
1309	YDR231C	Agreement	FALSE	1	0
1310	YKL003C	No agreement	FALSE	1	0
1311	YBR052C	Agreement	TRUE	1	0.001
1312	YLR205C	Agreement	FALSE	1	0
1313	YBL006C	No agreement	FALSE	1	0.004
1314	YMR271C	Agreement	TRUE	1	0
1315	YPL256C	Agreement	TRUE	1	0
1316	YDR196C	Agreement	FALSE	0	0
1317	YHL032C	Agreement	TRUE	1	0
1318	YBR122C	No agreement	FALSE	1	0

1319	YLR007W	No agreement	FALSE	0	0.009
1320	YGL127C	Agreement	FALSE	1	0.085
1321	YHR037W	No agreement	FALSE	1	0
1322	YOL113W	Agreement	FALSE	1	0
1323	YIL065C	Agreement	FALSE	0	0.001
1324	YKL120W	Agreement	TRUE	1	0.001
1325	YER054C	Agreement	FALSE	1	0
1326	YML068W	Agreement	FALSE	1	0.055
1327	YDL234C	Agreement	FALSE	1	0
1328	YLR345W	Agreement	FALSE	1	0
1329	YKR062W	No agreement	FALSE	0	0.088
1330	YLR225C	No agreement	FALSE	1	0.228
1331	YGR220C	No agreement	FALSE	1	0
1332	YJL016W	Agreement	TRUE	1	0
1333	YHR138C	Agreement	TRUE	1	0
1334	YLR394W	No agreement	FALSE	1	0
1335	YJR122W	Agreement	FALSE	1	0
1336	YOR157C	Agreement	FALSE	0	0
1337	YDR166C	Agreement	FALSE	0	0
1338	YDR292C	No agreement	FALSE	0	0
1339	YPR040W	No agreement	FALSE	1	0
1340	YMR186W	No agreement	FALSE	1	0
1341	YPL138C	No agreement	FALSE	1	0
1342	YDL091C	Agreement	FALSE	1	0
1343	YML129C	No agreement	TRUE	1	0
1344	YJR130C	No agreement	FALSE	1	0.026
1345	YGL050W	No agreement	FALSE	1	0.079
1346	YKR006C	No agreement	FALSE	1	0
1347	YML004C	Agreement	TRUE	1	0
1348	YNL073W	No agreement	FALSE	1	0
1349	YGL004C	Agreement	FALSE	1	0.263
1350	YGR017W	No agreement	FALSE	1	0
1351	YMR225C	No agreement	TRUE	1	0
1352	YJR102C	Agreement	FALSE	1	0.078
1353	YPL196W	Agreement	TRUE	1	0.004
1354	YOL007C	No agreement	TRUE	1	0
1355	YER052C	Agreement	TRUE	1	0
1356	YDR287W	No agreement	FALSE	1	0.073
1357	YCL051W	No agreement	FALSE	1	0.207
1358	YOR184W	Agreement	TRUE	1	0
1359	YMR228W	No agreement	FALSE	1	0
1360	YDR057W	Agreement	FALSE	1	0.04
1361	YGR048W	Agreement	FALSE	0	0.139
1362	YLL058W	No agreement	TRUE	1	0
1363	YOL072W	Agreement	FALSE	1	0.025
1364	YNR040W	No agreement	FALSE	1	0
1365	YNL311C	Agreement	TRUE	1	0.111
1366	YJR034W	No agreement	TRUE	0	0
1367	YHL030W	Agreement	TRUE	1	0.003

1368	YOR337W	No agreement	TRUE	1	0.018
1369	YDR115W	No agreement	FALSE	1	0
1370	YCR036W	Agreement	FALSE	1	0.246
1371	YCR091W	Agreement	TRUE	1	0
1372	YJL071W	No agreement	TRUE	1	0.017
1373	YKL194C	No agreement	FALSE	1	0
1374	YKR093W	No agreement	FALSE	1	0
1375	YKL208W	No agreement	FALSE	1	0.031
1376	YDR320C	Agreement	FALSE	1	0
1377	YNL134C	Agreement	TRUE	1	0
1378	YOR197W	Agreement	FALSE	1	0.051
1379	YPL056C	Agreement	TRUE	1	0.032
1380	YLR362W	Agreement	FALSE	1	0
1381	YOL109W	No agreement	FALSE	1	0.016
1382	YJR051W	No agreement	FALSE	1	0.196
1383	YGR235C	Agreement	FALSE	1	0.108
1384	YMR031C	Agreement	FALSE	1	0
1385	YPL123C	Agreement	TRUE	1	0
1386	YDR116C	No agreement	FALSE	1	0
1387	YOL087C	Agreement	TRUE	1	0
1388	YMR034C	No agreement	FALSE	1	0
1389	YMR197C	Agreement	TRUE	0	0
1390	YGR246C	Agreement	TRUE	0	0.029
1391	YPL152W	Agreement	FALSE	1	0
1392	YNL286W	Agreement	TRUE	1	0.017
1393	YJL011C	Agreement	TRUE	0	0.044
1394	YAL008W	Agreement	TRUE	1	0
1395	YNL169C	Agreement	FALSE	1	0
1396	YLL011W	Agreement	FALSE	0	0.039
1397	YHL004W	No agreement	FALSE	0	0
1398	YAR031W	Agreement	TRUE	1	0
1399	YDR005C	Agreement	FALSE	1	0
1400	YOR352W	Agreement	FALSE	1	0.006
1401	YHR104W	Agreement	TRUE	1	0
1402	YHR182W	No agreement	FALSE	1	0
1403	YJR004C	Agreement	FALSE	1	0.001
1404	YLR016C	No agreement	FALSE	1	0.136
1405	YPL052W	No agreement	FALSE	1	0
1406	YPL219W	Agreement	TRUE	1	0.008
1407	YMR229C	Agreement	FALSE	0	0.033
1408	YDL150W	Agreement	TRUE	0	0.033
1409	YNL075W	Agreement	FALSE	0	0.029
1410	YDR338C	Agreement	FALSE	1	0.009
1411	YJL094C	Agreement	FALSE	1	0
1412	YPR100W	No agreement	TRUE	1	0
1413	YPR067W	Agreement	TRUE	1	0.003
1414	YER049W	Agreement	TRUE	1	0.06
1415	YDR164C	Agreement	FALSE	0	0.153
1416	YBR096W	Agreement	TRUE	1	0

1417	YPL228W	Agreement	FALSE	0	0.093
1418	YPL079W	Agreement	FALSE	1	0.002
1419	YGL125W	Agreement	FALSE	1	0
1420	YNR047W	Agreement	TRUE	1	0.192
1421	YBR097W	Agreement	FALSE	1	0.008
1422	YPR179C	No agreement	FALSE	1	0.194
1423	YGR070W	Agreement	FALSE	1	0.005
1424	YKR043C	Agreement	TRUE	1	0.004
1425	YDL040C	No agreement	FALSE	1	0
1426	YLR399C	Agreement	TRUE	1	0
1427	YOR104W	No agreement	TRUE	1	0.013
1428	YOR162C	Agreement	TRUE	1	0
1429	YNL054W	No agreement	FALSE	1	0.079
1430	YMR290C	Agreement	TRUE	0	0.114
1431	YAL001C	Agreement	FALSE	0	0
1432	YCR090C	No agreement	FALSE	1	0.089
1433	YIR006C	Agreement	FALSE	0	0.25
1434	YMR001C	Agreement	FALSE	0	0
1435	YML008C	Agreement	TRUE	1	0
1436	YEL001C	No agreement	TRUE	1	0
1437	YOL110W	Agreement	FALSE	1	0
1438	YGR172C	No agreement	TRUE	0	0
1439	YNL331C	No agreement	TRUE	1	0.001
1440	YMR176W	Agreement	FALSE	1	0.001
1441	YLR024C	No agreement	TRUE	1	0.046
1442	YER139C	No agreement	FALSE	1	0.003
1443	YML029W	No agreement	FALSE	1	0.032
1444	YJR154W	Agreement	FALSE	1	0
1445	YGR032W	No agreement	FALSE	1	0
1446	YGR086C	Agreement	TRUE	1	0
1447	YDR108W	Agreement	FALSE	1	0.24
1448	YLR017W	Agreement	TRUE	1	0.036
1449	YOR284W	No agreement	FALSE	1	0.086
1450	YBR234C	Agreement	FALSE	0	0.034
1451	YJL092W	No agreement	TRUE	1	0
1452	YJR074W	Agreement	FALSE	1	0.008
1453	YBL017C	Agreement	FALSE	1	0.26
1454	YKL041W	No agreement	FALSE	1	0.16
1455	YDR147W	Agreement	TRUE	1	0.004
1456	YHR006W	No agreement	TRUE	1	0.002
1457	YJL198W	Agreement	FALSE	1	0.021
1458	YDR420W	Agreement	FALSE	1	0.027
1459	YMR108W	Agreement	TRUE	1	0
1460	YMR038C	No agreement	FALSE	1	0.013
1461	YOR064C	No agreement	FALSE	1	0.134
1462	YJL096W	No agreement	TRUE	1	0
1463	YMR136W	Agreement	TRUE	1	0
1464	YBR235W	Agreement	TRUE	1	0.001
1465	YNL053W	No agreement	FALSE	0	0.275

1466	YDL215C	Agreement	TRUE	1	0
1467	YLR439W	No agreement	TRUE	1	0
1468	YNL298W	Agreement	TRUE	1	0
1469	YLR450W	Agreement	FALSE	1	0
1470	YNL086W	No agreement	TRUE	0	0
1471	YMR288W	No agreement	FALSE	0	0.007
1472	YMR169C	Agreement	FALSE	1	0
1473	YCL044C	No agreement	FALSE	1	0
1474	YJL146W	Agreement	FALSE	1	0.22
1475	YBR025C	Agreement	FALSE	1	0
1476	YBL042C	Agreement	FALSE	1	0.034
1477	YKL068W	Agreement	FALSE	1	0.167
1478	YBR287W	Agreement	FALSE	1	0.107
1479	YEL063C	Agreement	FALSE	1	0.006
1480	YBR267W	Agreement	FALSE	1	0.083
1481	YNL219C	Agreement	FALSE	1	0.254
1482	YGL192W	Agreement	FALSE	0	0
1483	YBL046W	No agreement	FALSE	1	0.066
1484	YCR087C-A	Agreement	FALSE	1	0.045
1485	YGR281W	Agreement	FALSE	1	0
1486	YJR088C	Agreement	TRUE	1	0.118
1487	YGR138C	No agreement	TRUE	1	0.188
1488	YOR355W	No agreement	FALSE	1	0
1489	YDR270W	Agreement	FALSE	1	0.145
1490	YNL207W	Agreement	FALSE	0	0.044
1491	YOR310C	Agreement	FALSE	0	0.003
1492	YCR030C	Agreement	FALSE	1	0
1493	YGL173C	Agreement	FALSE	1	0.132
1494	YKR048C	No agreement	TRUE	1	0.004
1495	YMR088C	Agreement	FALSE	1	0.003
1496	YMR112C	Agreement	TRUE	0	0
1497	YIR011C	No agreement	FALSE	0	0.213
1498	YOL025W	No agreement	TRUE	1	0
1499	YGL221C	No agreement	TRUE	1	0.247
1500	YNR067C	No agreement	FALSE	1	0
1501	YMR311C	Agreement	TRUE	1	0
1502	YJR096W	No agreement	FALSE	1	0
1503	YNL284C	No agreement	FALSE	0	0
1504	YMR026C	No agreement	FALSE	1	0.206
1505	YLR441C	Agreement	FALSE	1	0
1506	YOR016C	No agreement	FALSE	1	0.003
1507	YML052W	Agreement	TRUE	1	0
1508	YDL130W-A	Agreement	FALSE	1	0
1509	YLR361C	Agreement	FALSE	1	0.279
1510	YJL050W	Agreement	TRUE	0	0.047
1511	YLR283W	No agreement	TRUE	1	0.211
1512	YGR191W	Agreement	FALSE	0	0.031
1513	YHR009C	No agreement	TRUE	1	0
1514	YKL058W	No agreement	TRUE	0	0.009

1515	YJL098W	Agreement	TRUE	1	0.017
1516	YDL082W	Agreement	TRUE	1	0
1517	YGR127W	Agreement	FALSE	1	0
1518	YBR229C	Agreement	FALSE	1	0
1519	YLR175W	Agreement	FALSE	0	0.002
1520	YHR194W	Agreement	FALSE	1	0.172
1521	YDL139C	No agreement	FALSE	0	0.249
1522	YLR314C	No agreement	TRUE	0	0
1523	YHR103W	No agreement	FALSE	1	0
1524	YKL035W	Agreement	TRUE	0	0.002
1525	YGL154C	Agreement	FALSE	1	0
1526	YGL030W	No agreement	TRUE	0	0.113
1527	YGL147C	Agreement	FALSE	1	0.02
1528	YKL182W	Agreement	FALSE	0	0.007
1529	YPL105C	No agreement	FALSE	1	0.114
1530	YEL065W	No agreement	FALSE	1	0.009
1531	YFR010W	Agreement	TRUE	1	0.047
1532	YPR097W	Agreement	TRUE	1	0
1533	YOR298C-A	No agreement	FALSE	1	0
1534	YPL119C	Agreement	TRUE	1	0
1535	YKL128C	Agreement	TRUE	1	0
1536	YJL068C	Agreement	FALSE	1	0.002
1537	YER053C	Agreement	TRUE	1	0.001
1538	YOR086C	Agreement	FALSE	1	0
1539	YDR171W	Agreement	TRUE	1	0
1540	YJL041W	No agreement	FALSE	0	0.088
1541	YML014W	No agreement	TRUE	1	0.018
1542	YDR182W	Agreement	TRUE	0	0.034
1543	YHR148W	Agreement	FALSE	0	0.03
1544	YLR295C	No agreement	TRUE	1	0
1545	YNL049C	No agreement	FALSE	1	0.018
1546	YNL152W	Agreement	TRUE	0	0.108
1547	YPL093W	Agreement	FALSE	0	0.039
1548	YJL019W	Agreement	FALSE	0	0
1549	YOR223W	Agreement	FALSE	1	0
1550	YGL010W	Agreement	FALSE	1	0
1551	YKL160W	No agreement	TRUE	1	0.068
1552	YBL038W	No agreement	TRUE	1	0
1553	YKL023W	No agreement	TRUE	1	0.006
1554	YCR004C	Agreement	FALSE	1	0
1555	YOL084W	No agreement	FALSE	1	0
1556	YOR022C	Agreement	FALSE	1	0.246
1557	YHR131C	Agreement	FALSE	1	0.097
1558	YJL052W	Agreement	FALSE	1	0.001
1559	YGR116W	Agreement	TRUE	0	0.185
1560	YFL028C	No agreement	TRUE	1	0.072
1561	YBL105C	Agreement	FALSE	0	0.265
1562	YNR013C	Agreement	FALSE	1	0.005
1563	YLR009W	Agreement	FALSE	0	0.046

1564	YDL193W	Agreement	TRUE	0	0
1565	YDR434W	No agreement	TRUE	0	0.062
1566	YEL071W	Agreement	TRUE	1	0
1567	YIR031C	No agreement	TRUE	1	0.004
1568	YDL179W	No agreement	FALSE	1	0
1569	YLR056W	No agreement	TRUE	1	0
1570	YBR262C	No agreement	FALSE	1	0
1571	YKL039W	No agreement	FALSE	1	0.099
1572	YOR117W	Agreement	FALSE	0	0.003
1573	YBR003W	No agreement	TRUE	1	0
1574	YML100W	Agreement	FALSE	1	0
1575	YGR183C	Agreement	TRUE	1	0
1576	YDL002C	Agreement	FALSE	1	0.223
1577	YKL175W	No agreement	FALSE	1	0.032
1578	YHR059W	No agreement	FALSE	1	0
1579	YMR280C	Agreement	TRUE	1	0.001
1580	YDR083W	Agreement	FALSE	1	0.046
1581	YGR159C	Agreement	FALSE	1	0.041
1582	YPR093C	No agreement	TRUE	1	0
1583	YML034W	Agreement	TRUE	1	0
1584	YGR038W	No agreement	TRUE	1	0.001
1585	YHR089C	Agreement	FALSE	0	0.005
1586	YNL074C	No agreement	FALSE	1	0.087
1587	YOR164C	Agreement	TRUE	1	0
1588	YDR098C	Agreement	FALSE	1	0
1589	YMR231W	Agreement	FALSE	1	0.215
1590	YHR154W	Agreement	TRUE	1	0
1591	YMR160W	Agreement	FALSE	1	0.001
1592	YNL161W	No agreement	FALSE	0	0.243
1593	YMR058W	No agreement	FALSE	1	0
1594	YAL036C	Agreement	FALSE	1	0.001
1595	YGL248W	Agreement	TRUE	1	0.011
1596	YPL100W	Agreement	TRUE	1	0
1597	YBR059C	Agreement	TRUE	1	0
1598	YLR057W	Agreement	TRUE	1	0.217
1599	YGL122C	No agreement	TRUE	0	0.004
1600	YHR002W	Agreement	TRUE	0	0.001
1601	YDR137W	Agreement	FALSE	1	0.177
1602	YKL038W	Agreement	FALSE	1	0.019
1603	YGR001C	No agreement	FALSE	1	0.001
1604	YER091C	Agreement	FALSE	1	0
1605	YDL202W	No agreement	TRUE	1	0
1606	YLR044C	Agreement	FALSE	1	0.004
1607	YBL028C	Agreement	TRUE	1	0.039
1608	YJL097W	No agreement	FALSE	0	0.183
1609	YLR037C	Agreement	FALSE	1	0
1610	YDR140W	No agreement	TRUE	1	0.083
1611	YOR132W	Agreement	TRUE	1	0.23
1612	YIL108W	Agreement	FALSE	1	0.069

1613	YPL260W	Agreement	TRUE	1	0.005
1614	YDL159W	Agreement	FALSE	1	0.27
1615	YDR156W	Agreement	FALSE	1	0.028
1616	YGR186W	No agreement	TRUE	0	0.035
1617	YMR316W	No agreement	FALSE	1	0.275
1618	YNL177C	No agreement	TRUE	1	0
1619	YLR025W	No agreement	TRUE	1	0.076
1620	YIL140W	Agreement	FALSE	1	0
1621	YKL034W	Agreement	FALSE	1	0.022
1622	YDL146W	Agreement	FALSE	1	0.118
1623	YLR023C	Agreement	FALSE	1	0
1624	YGL141W	Agreement	FALSE	1	0
1625	YIL040W	No agreement	FALSE	1	0.127
1626	YPR119W	Agreement	FALSE	1	0
1627	YHL002W	Agreement	FALSE	1	0.081
1628	YPL084W	Agreement	TRUE	1	0.004
1629	YFL034C-A	Agreement	TRUE	1	0
1630	YMR079W	Agreement	FALSE	0	0.002
1631	YLR177W	Agreement	TRUE	1	0
1632	YGL231C	No agreement	FALSE	1	0.065
1633	YBR205W	No agreement	TRUE	1	0
1634	YCR009C	Agreement	FALSE	1	0.132
1635	YMR193W	No agreement	TRUE	1	0
1636	YPL091W	No agreement	FALSE	1	0.004
1637	YOR161C	Agreement	FALSE	1	0
1638	YGL065C	Agreement	FALSE	0	0
1639	YIL010W	Agreement	TRUE	1	0.117
1640	YCR002C	No agreement	TRUE	1	0
1641	YKL141W	No agreement	FALSE	0	0
1642	YCR019W	No agreement	TRUE	1	0.007
1643	YPR073C	No agreement	TRUE	1	0.041
1644	YER175C	No agreement	TRUE	1	0.003
1645	YOR359W	Agreement	FALSE	1	0.05
1646	YER006W	Agreement	FALSE	0	0.03
1647	YGR233C	No agreement	FALSE	1	0
1648	YCL052C	Agreement	TRUE	0	0.007
1649	YPL149W	No agreement	FALSE	1	0.15
1650	YBR149W	Agreement	TRUE	1	0
1651	YMR276W	Agreement	TRUE	1	0.001
1652	YDR251W	No agreement	FALSE	1	0.073
1653	YHL020C	No agreement	TRUE	1	0.033
1654	YLR176C	No agreement	TRUE	1	0
1655	YBR236C	No agreement	FALSE	0	0.036
1656	YMR091C	No agreement	TRUE	1	0.065
1657	YGR078C	No agreement	TRUE	1	0.035
1658	YDL126C	Agreement	TRUE	0	0.013
1659	YAR014C	No agreement	TRUE	1	0.008
1660	YIL158W	Agreement	TRUE	0	0
1661	YBR077C	Agreement	TRUE	1	0

1662	YOR014W	Agreement	TRUE	1	0.002
1663	YNL063W	Agreement	FALSE	1	0.156
1664	YGR120C	No agreement	FALSE	0	0
1665	YPL113C	No agreement	FALSE	1	0
1666	YBR158W	No agreement	FALSE	1	0
1667	YNL061W	Agreement	FALSE	0	0.012
1668	YMR227C	No agreement	TRUE	0	0.23
1669	YOL076W	Agreement	FALSE	1	0.266
1670	YJL149W	Agreement	TRUE	1	0.004
1671	YGL159W	No agreement	FALSE	1	0.054
1672	YOR143C	No agreement	TRUE	0	0
1673	YGR105W	No agreement	FALSE	1	0.049
1674	YOR141C	No agreement	TRUE	1	0
1675	YOL054W	No agreement	TRUE	1	0
1676	YML027W	Agreement	FALSE	1	0
1677	YDR328C	Agreement	TRUE	0	0
1678	YLR166C	No agreement	FALSE	0	0.078
1679	YIR037W	Agreement	FALSE	1	0
1680	YGR142W	No agreement	FALSE	1	0
1681	YIL092W	No agreement	FALSE	1	0.002
1682	YNL055C	Agreement	TRUE	0	0
1683	YPL207W	No agreement	FALSE	1	0
1684	YOR293W	Agreement	FALSE	1	0
1685	YPL024W	Agreement	FALSE	1	0.282
1686	YEL026W	Agreement	FALSE	0	0.002
1687	YKL100C	Agreement	TRUE	1	0
1688	YJR151C	Agreement	FALSE	0	0.003
1689	YPR108W	Agreement	TRUE	0	0.003
1690	YPL247C	Agreement	FALSE	1	0
1691	YCL028W	Agreement	FALSE	1	0.027
1692	YPR133W-A	No agreement	TRUE	0	0
1693	YNL251C	No agreement	FALSE	0	0.059
1694	YNL141W	Agreement	FALSE	1	0.093
1695	YDR195W	No agreement	FALSE	1	0.154
1696	YPL063W	No agreement	TRUE	0	0
1697	YLR001C	Agreement	TRUE	1	0.001
1698	YPL104W	No agreement	FALSE	1	0
1699	YDR091C	Agreement	FALSE	0	0.003
1700	YDR165W	Agreement	TRUE	1	0.049
1701	YMR135C	Agreement	TRUE	1	0
1702	YGR205W	Agreement	FALSE	1	0
1703	YCR060W	No agreement	FALSE	1	0.005
1704	YDL208W	Agreement	FALSE	0	0.004
1705	YDL010W	Agreement	TRUE	1	0
1706	YGR180C	Agreement	TRUE	1	0.003
1707	YJL201W	No agreement	FALSE	1	0.001
1708	YJR059W	Agreement	TRUE	1	0.008
1709	YNL122C	No agreement	FALSE	1	0
1710	YHR178W	Agreement	FALSE	1	0

1711	YDR189W	Agreement	TRUE	0	0.034
1712	YLR387C	No agreement	TRUE	1	0.144
1713	YLR457C	No agreement	FALSE	0	0.163
1714	YDR306C	Agreement	FALSE	1	0
1715	YDR172W	Agreement	TRUE	0	0.256
1716	YBL090W	No agreement	TRUE	1	0
1717	YLL029W	Agreement	FALSE	1	0.139
1718	YOR191W	Agreement	FALSE	1	0.057
1719	YCR026C	No agreement	FALSE	1	0.13
1720	YBR142W	Agreement	TRUE	0	0.145
1721	YKL119C	Agreement	TRUE	1	0.024
1722	YIL121W	No agreement	FALSE	1	0.009
1723	YGL045W	No agreement	TRUE	1	0
1724	YOL042W	No agreement	FALSE	1	0.257
1725	YMR211W	No agreement	TRUE	0	0.032
1726	YGL254W	No agreement	FALSE	1	0.185
1727	YKL090W	No agreement	TRUE	1	0.036
1728	YKL099C	Agreement	FALSE	0	0.078
1729	YDR375C	No agreement	TRUE	1	0
1730	YNL052W	No agreement	FALSE	1	0
1731	YGR044C	No agreement	FALSE	1	0
1732	YPL089C	No agreement	TRUE	1	0.002
1733	YER008C	No agreement	FALSE	0	0.209
1734	YNL297C	Agreement	TRUE	1	0.001
1735	YML080W	Agreement	FALSE	1	0.092
1736	YGR263C	No agreement	FALSE	1	0.214
1737	YKR102W	Agreement	FALSE	1	0
1738	YNL110C	Agreement	TRUE	0	0.034
1739	YIL142W	Agreement	FALSE	0	0.102
1740	YKR058W	Agreement	FALSE	1	0
1741	YEL044W	Agreement	FALSE	1	0
1742	YDL134C	No agreement	TRUE	1	0.001
1743	YAL035W	Agreement	TRUE	1	0.095
1744	YLR186W	Agreement	FALSE	0	0.027
1745	YLR218C	Agreement	TRUE	1	0
1746	YMR109W	Agreement	FALSE	1	0
1747	YER140W	No agreement	FALSE	1	0.164
1748	YGL043W	No agreement	TRUE	1	0.001
1749	YKL112W	Agreement	TRUE	0	0.041
1750	YJL111W	No agreement	FALSE	0	0.267
1751	YKR056W	Agreement	TRUE	1	0.041
1752	YBL081W	Agreement	FALSE	1	0.022
1753	YDR484W	No agreement	FALSE	1	0
1754	YKL191W	No agreement	FALSE	1	0.003
1755	YGL262W	Agreement	FALSE	1	0.013
1756	YHR116W	Agreement	TRUE	1	0
1757	YOL080C	Agreement	FALSE	1	0.164
1758	YLR442C	No agreement	FALSE	1	0.053
1759	YBR195C	No agreement	FALSE	1	0.052

1760	YLR084C	Agreement	FALSE	1	0
1761	YPR061C	Agreement	FALSE	1	0
1762	YER112W	Agreement	TRUE	0	0.09
1763	YOL013C	No agreement	FALSE	1	0
1764	YDR318W	Agreement	FALSE	1	0.005
1765	YNR009W	Agreement	FALSE	1	0
1766	YAL034C	Agreement	TRUE	1	0
1767	YER018C	No agreement	TRUE	0	0
1768	YCR079W	Agreement	FALSE	1	0
1769	YLR085C	No agreement	FALSE	1	0.221
1770	YGL079W	No agreement	FALSE	1	0.012
1771	YDR319C	Agreement	FALSE	1	0
1772	YML067C	No agreement	FALSE	1	0
1773	YOR070C	Agreement	TRUE	1	0.005
1774	YER001W	No agreement	FALSE	1	0
1775	YMR116C	Agreement	TRUE	1	0
1776	YMR188C	No agreement	TRUE	1	0
1777	YNL025C	No agreement	TRUE	1	0.023
1778	YFL046W	Agreement	TRUE	1	0.023
1779	YMR162C	Agreement	FALSE	1	0.003
1780	YKL028W	No agreement	TRUE	0	0.009
1781	YGL054C	Agreement	FALSE	1	0.289
1782	YLR028C	Agreement	TRUE	1	0
1783	YGL257C	No agreement	FALSE	1	0.136
1784	YGR163W	No agreement	TRUE	1	0
1785	YOR354C	No agreement	FALSE	1	0
1786	YMR055C	No agreement	FALSE	1	0
1787	YMR035W	No agreement	FALSE	1	0.25
1788	YNL190W	No agreement	FALSE	1	0
1789	YNL007C	No agreement	TRUE	0	0
1790	YKL040C	No agreement	TRUE	1	0.127
1791	YCR093W	Agreement	FALSE	0	0
1792	YPL234C	Agreement	FALSE	1	0.252
1793	YDR047W	No agreement	FALSE	0	0.283
1794	YDL189W	No agreement	TRUE	1	0.046
1795	YDL153C	Agreement	TRUE	0	0.022
1796	YGR146C	No agreement	TRUE	1	0
1797	YOR313C	No agreement	TRUE	1	0.055
1798	YOR009W	Agreement	FALSE	1	0
1799	YGR042W	Agreement	FALSE	1	0.122
1800	YPL206C	Agreement	FALSE	1	0.139
1801	YLR036C	Agreement	TRUE	1	0
1802	YDR453C	Agreement	TRUE	1	0
1803	YNL101W	No agreement	FALSE	1	0.001
1804	YMR005W	No agreement	TRUE	0	0.007
1805	YDR404C	No agreement	FALSE	0	0.012
1806	YML107C	Agreement	FALSE	1	0.145
1807	YMR028W	No agreement	FALSE	0	0.277
1808	YDR464W	No agreement	FALSE	0	0.252

1809	YAR029W	Agreement	TRUE	1	0.054
1810	YIL115C	Agreement	FALSE	0	0.108
1811	YDR352W	No agreement	FALSE	1	0.003
1812	YBR225W	Agreement	TRUE	1	0
1813	YLL004W	No agreement	FALSE	0	0.185
1814	YJL109C	Agreement	TRUE	0	0.038
1815	YBR289W	Agreement	TRUE	1	0.002
1816	YFL027C	No agreement	TRUE	1	0.005
1817	YHR025W	No agreement	TRUE	1	0
1818	YIL030C	No agreement	FALSE	1	0.131
1819	YLR289W	No agreement	TRUE	1	0
1820	YML038C	Agreement	FALSE	1	0.135
1821	YGL031C	Agreement	FALSE	1	0
1822	YPR138C	No agreement	FALSE	1	0.177
1823	YNL002C	Agreement	TRUE	0	0.036
1824	YBL020W	No agreement	FALSE	0	0
1825	YDR129C	Agreement	TRUE	1	0
1826	YDL203C	No agreement	FALSE	1	0.284
1827	YNL300W	Agreement	FALSE	1	0
1828	YER136W	Agreement	TRUE	0	0.006
1829	YPL241C	Agreement	TRUE	1	0
1830	YDR502C	Agreement	FALSE	0	0
1831	YBR221C	No agreement	FALSE	1	0
1832	YOR302W	No agreement	FALSE	1	0
1833	YBR172C	No agreement	TRUE	1	0.003
1834	YPR022C	No agreement	TRUE	1	0.004
1835	YDL066W	Agreement	TRUE	1	0
1836	YMR183C	Agreement	FALSE	1	0.009
1837	YDL047W	No agreement	FALSE	1	0.181
1838	YOR245C	Agreement	TRUE	1	0.011
1839	YNL005C	No agreement	FALSE	1	0
1840	YEL047C	Agreement	FALSE	1	0.101
1841	YOR229W	No agreement	TRUE	1	0.01
1842	YDL045W-A	No agreement	FALSE	1	0
1843	YJL143W	No agreement	FALSE	0	0
1844	YDR232W	No agreement	FALSE	0	0.09
1845	YFR005C	Agreement	FALSE	0	0.059
1846	YER126C	Agreement	FALSE	0	0.038
1847	YDL141W	No agreement	FALSE	0	0.004
1848	YLR183C	Agreement	TRUE	1	0
1849	YOR153W	Agreement	FALSE	1	0.001
1850	YLR018C	No agreement	TRUE	1	0.128
1851	YNR031C	Agreement	TRUE	1	0.026
1852	YHR054C	No agreement	TRUE	0	0.099
1853	YEL050C	No agreement	TRUE	1	0
1854	YPL110C	Agreement	TRUE	1	0.001
1855	YMR092C	Agreement	FALSE	1	0
1856	YER099C	No agreement	FALSE	0	0.135
1857	YLR256W	Agreement	TRUE	0	0

1858	YCR017C	Agreement	FALSE	1	0.003
1859	YHR001W	Agreement	FALSE	0	0.001
1860	YJL072C	Agreement	FALSE	0	0
1861	YJL081C	Agreement	TRUE	0	0.056
1862	YFL021W	Agreement	FALSE	1	0.03
1863	YCR024C	No agreement	TRUE	1	0
1864	YHR196W	Agreement	FALSE	0	0.035
1865	YNL051W	Agreement	TRUE	1	0
1866	YGR111W	Agreement	FALSE	1	0.053
1867	YIR042C	No agreement	FALSE	1	0
1868	YNL149C	Agreement	FALSE	0	0.008
1869	YER059W	No agreement	FALSE	1	0.002
1870	YDL133W	Agreement	FALSE	1	0
1871	YPL002C	No agreement	FALSE	1	0.132
1872	YJR082C	No agreement	FALSE	1	0
1873	YNL258C	Agreement	FALSE	0	0.126
1874	YJL053W	Agreement	TRUE	1	0.022
1875	YLR170C	Agreement	TRUE	1	0
1876	YPR102C	Agreement	FALSE	0	0
1877	YGR135W	No agreement	FALSE	1	0.139
1878	YPL040C	No agreement	TRUE	1	0
1879	YNL253W	Agreement	FALSE	1	0.124
1880	YDL058W	Agreement	TRUE	0	0.2
1881	YNL178W	Agreement	FALSE	0	0.001
1882	YDR246W	No agreement	TRUE	0	0
1883	YDL104C	No agreement	FALSE	1	0.006
1884	YDR141C	Agreement	TRUE	0	0.001
1885	YJR078W	No agreement	FALSE	1	0
1886	YDR162C	No agreement	TRUE	1	0
1887	YOR372C	Agreement	FALSE	0	0.206
1888	YJR001W	Agreement	FALSE	1	0.266
1889	YDL077C	Agreement	FALSE	1	0
1890	YBL040C	No agreement	TRUE	0	0.012
1891	YOR251C	Agreement	FALSE	1	0
1892	YGR013W	No agreement	TRUE	0	0.082
1893	YIR027C	No agreement	FALSE	1	0.026
1894	YHR004C	Agreement	TRUE	1	0.003
1895	YGR240C	No agreement	TRUE	1	0.025
1896	YPR145W	Agreement	FALSE	1	0
1897	YOL135C	No agreement	TRUE	0	0.009
1898	YPL058C	Agreement	FALSE	1	0.025
1899	YLR168C	Agreement	TRUE	1	0
1900	YMR295C	No agreement	TRUE	1	0
1901	YNL081C	No agreement	FALSE	1	0
1902	YPR120C	No agreement	TRUE	1	0
1903	YOR065W	Agreement	TRUE	1	0
1904	YBL088C	Agreement	TRUE	1	0.043
1905	YOR259C	Agreement	TRUE	0	0.048
1906	YKR080W	Agreement	TRUE	1	0.013

1907	YIL096C	Agreement	FALSE	1	0.077
1908	YJR031C	No agreement	FALSE	1	0
1909	YDL008W	No agreement	FALSE	0	0.217
1910	YLR246W	No agreement	FALSE	1	0.142
1911	YOL088C	Agreement	FALSE	1	0
1912	YPL076W	No agreement	TRUE	0	0
1913	YNL167C	Agreement	TRUE	1	0.001
1914	YMR072W	Agreement	FALSE	1	0
1915	YLR192C	Agreement	TRUE	1	0.071
1916	YHL015W	Agreement	FALSE	0	0.001
1917	YLL040C	Agreement	TRUE	1	0
1918	YOL138C	Agreement	FALSE	1	0.069
1919	YJR113C	No agreement	FALSE	1	0
1920	YGL114W	No agreement	FALSE	1	0.054
1921	YDL235C	No agreement	FALSE	0	0.015
1922	YML112W	No agreement	FALSE	1	0.02
1923	YBL093C	No agreement	TRUE	1	0.086
1924	YOR074C	Agreement	TRUE	0	0
1925	YGL208W	Agreement	FALSE	1	0.017
1926	YGR284C	No agreement	FALSE	1	0.001
1927	YMR008C	Agreement	FALSE	1	0.157
1928	YPL181W	No agreement	TRUE	1	0.012
1929	YKR104W	Agreement	FALSE	1	0.002
1930	YHR106W	Agreement	FALSE	1	0
1931	YOL036W	No agreement	TRUE	1	0.076
1932	YGL179C	No agreement	FALSE	1	0.001
1933	YLR074C	Agreement	TRUE	1	0.083
1934	YIL004C	Agreement	TRUE	0	0.02
1935	YLR073C	Agreement	FALSE	1	0.056
1936	YHR091C	No agreement	TRUE	1	0
1937	YER039C	No agreement	TRUE	1	0
1938	YOR260W	Agreement	FALSE	0	0.005
1939	YDR409W	Agreement	FALSE	1	0
1940	YFR041C	No agreement	FALSE	1	0.015
1941	YPL259C	Agreement	TRUE	1	0.031
1942	YKL174C	No agreement	FALSE	1	0.287
1943	YLR004C	No agreement	FALSE	1	0
1944	YAL042W	No agreement	FALSE	1	0.285
1945	YDR376W	No agreement	TRUE	0	0
1946	YNL129W	Agreement	TRUE	1	0.044
1947	YIL038C	No agreement	FALSE	1	0.267
1948	YMR308C	Agreement	FALSE	0	0.006
1949	YNL127W	Agreement	FALSE	1	0.166
1950	YCL056C	Agreement	TRUE	1	0.001
1951	YHR051W	No agreement	TRUE	1	0
1952	YOR321W	No agreement	FALSE	1	0.195
1953	YMR150C	Agreement	FALSE	1	0
1954	YGR029W	Agreement	TRUE	0	0
1955	YMR258C	Agreement	FALSE	1	0

1956	YNL192W	No agreement	FALSE	1	0.001
1957	YOL019W	Agreement	FALSE	1	0
1958	YOR244W	Agreement	TRUE	0	0.008
1959	YPR162C	No agreement	TRUE	0	0.075
1960	YNR015W	Agreement	TRUE	1	0.002
1961	YFR036W	No agreement	TRUE	1	0.028
1962	YBR202W	Agreement	FALSE	0	0.007
1963	YMR285C	No agreement	TRUE	1	0.004
1964	YOL012C	No agreement	FALSE	0	0
1965	YML036W	Agreement	FALSE	1	0.07
1966	YPL175W	No agreement	FALSE	0	0.119
1967	YDL055C	Agreement	FALSE	0	0.005
1968	YBR026C	Agreement	FALSE	1	0
1969	YMR110C	Agreement	TRUE	1	0
1970	YJR140C	No agreement	TRUE	1	0.004
1971	YOR204W	No agreement	FALSE	0	0.005
1972	YJL006C	No agreement	FALSE	1	0.128
1973	YOR181W	Agreement	TRUE	0	0.228
1974	YKR051W	Agreement	TRUE	1	0
1975	YJR062C	No agreement	FALSE	1	0.081
1976	YGL084C	Agreement	FALSE	1	0.032
1977	YJR066W	Agreement	FALSE	1	0.002
1978	YER027C	No agreement	FALSE	1	0.113
1979	YOR371C	No agreement	TRUE	1	0
1980	YDR465C	Agreement	FALSE	1	0.017
1981	YGL250W	Agreement	TRUE	1	0
1982	YPR127W	Agreement	TRUE	1	0
1983	YEL020W-A	No agreement	FALSE	0	0
1984	YMR210W	Agreement	TRUE	1	0
1985	YBR028C	Agreement	TRUE	1	0
1986	YMR024W	No agreement	TRUE	1	0
1987	YHR045W	Agreement	FALSE	1	0
1988	YPL046C	No agreement	TRUE	1	0
1989	YDR174W	No agreement	FALSE	1	0.001
1990	YGL252C	Agreement	FALSE	1	0.009
1991	YCL008C	No agreement	TRUE	1	0.003
1992	YMR205C	Agreement	FALSE	1	0.01
1993	YDR272W	Agreement	TRUE	1	0
1994	YGR100W	No agreement	FALSE	1	0.24
1995	YKR021W	No agreement	FALSE	1	0.004
1996	YPR189W	No agreement	TRUE	1	0.115
1997	YKL204W	Agreement	TRUE	1	0
1998	YPL066W	Agreement	FALSE	1	0.026
1999	YKR014C	Agreement	TRUE	1	0
2000	YOR004W	Agreement	TRUE	0	0.037
2001	YFR019W	Agreement	TRUE	1	0.02
2002	YER016W	No agreement	TRUE	1	0
2003	YHL003C	No agreement	FALSE	1	0.025
2004	YHR195W	No agreement	TRUE	1	0

2005	YAR007C	Agreement	FALSE	0	0.283
2006	YDL161W	Agreement	TRUE	1	0.008
2007	YBR016W	Agreement	TRUE	1	0.045
2008	YFL030W	No agreement	TRUE	1	0
2009	YBL051C	Agreement	FALSE	1	0.165
2010	YNL216W	Agreement	FALSE	0	0.008
2011	YNL076W	Agreement	TRUE	1	0.045
2012	YPL162C	Agreement	FALSE	1	0
2013	YOR083W	Agreement	FALSE	1	0.031
2014	YMR257C	No agreement	FALSE	1	0
2015	YGL104C	Agreement	FALSE	1	0.001
2016	YHR011W	No agreement	TRUE	1	0
2017	YDR058C	No agreement	FALSE	1	0.247
2018	YHR019C	Agreement	FALSE	0	0
2019	YLR239C	No agreement	TRUE	1	0
2020	YJL193W	Agreement	TRUE	1	0.002
2021	YGL105W	No agreement	FALSE	1	0.171
2022	YHL033C	Agreement	FALSE	1	0
2023	YPL204W	No agreement	TRUE	0	0.014
2024	YNL151C	Agreement	TRUE	0	0.026
2025	YML114C	Agreement	FALSE	0	0.02
2026	YDR148C	No agreement	FALSE	1	0.001
2027	YIL050W	No agreement	TRUE	1	0.035
2028	YNL102W	Agreement	TRUE	0	0
2029	YNL159C	Agreement	FALSE	1	0.001
2030	YOR320C	No agreement	TRUE	1	0.004
2031	YJR136C	Agreement	TRUE	0	0
2032	YMR222C	Agreement	FALSE	1	0.01
2033	YOR175C	Agreement	FALSE	1	0.004
2034	YLR195C	No agreement	TRUE	0	0
2035	YMR056C	Agreement	FALSE	1	0
2036	YHR113W	No agreement	FALSE	1	0.015
2037	YOR308C	Agreement	TRUE	1	0
2038	YBL099W	Agreement	FALSE	1	0
2039	YLR266C	Agreement	FALSE	1	0.01
2040	YCL037C	Agreement	FALSE	1	0.026
2041	YML016C	No agreement	FALSE	1	0.051
2042	YOL062C	No agreement	TRUE	1	0
2043	YNL308C	Agreement	TRUE	0	0.09
2044	YGR266W	Agreement	TRUE	1	0
2045	YGR181W	No agreement	TRUE	1	0
2046	YER172C	No agreement	FALSE	0	0.205
2047	YOR093C	No agreement	TRUE	1	0.009
2048	YGL056C	Agreement	TRUE	1	0.001
2049	YML120C	Agreement	FALSE	1	0
2050	YBR058C	Agreement	FALSE	1	0.187
2051	YHR158C	No agreement	TRUE	1	0.02
2052	YGL181W	Agreement	FALSE	1	0.272
2053	YOL075C	Agreement	FALSE	1	0.217

2054	YNL036W	No agreement	FALSE	0	0
2055	YHR204W	Agreement	FALSE	1	0
2056	YGL129C	No agreement	TRUE	1	0
2057	YMR230W	Agreement	FALSE	1	0.001
2058	YEL051W	No agreement	FALSE	1	0.06
2059	YEL056W	No agreement	TRUE	1	0.004
2060	YMR113W	Agreement	TRUE	0	0.092
2061	YGR197C	Agreement	FALSE	1	0.007
2062	YBR161W	Agreement	FALSE	1	0
2063	YPL065W	Agreement	TRUE	1	0
2064	YHR199C	Agreement	FALSE	1	0.287
2065	YOR319W	No agreement	TRUE	0	0.101
2066	YAR020C	Agreement	TRUE	1	0.004
2067	YHR067W	Agreement	FALSE	1	0.001
2068	YIR023W	Agreement	TRUE	1	0.001
2069	YPL145C	No agreement	FALSE	1	0
2070	YGR199W	No agreement	FALSE	1	0
2071	YFR048W	No agreement	FALSE	1	0.04
2072	YCR033W	Agreement	FALSE	1	0.082
2073	YBL004W	Agreement	FALSE	0	0.034
2074	YLR240W	Agreement	TRUE	1	0.006
2075	YPL043W	Agreement	TRUE	0	0.089
2076	YDR400W	No agreement	FALSE	1	0
2077	YBR070C	Agreement	TRUE	0	0.004
2078	YDR499W	Agreement	TRUE	0	0.003
2079	YDR077W	Agreement	FALSE	1	0
2080	YPL218W	Agreement	FALSE	0	0.014
2081	YBL079W	Agreement	FALSE	1	0.245
2082	YHR174W	Agreement	FALSE	0	0.228
2083	YNL264C	Agreement	FALSE	1	0.21
2084	YDR430C	No agreement	FALSE	1	0
2085	YLL054C	No agreement	FALSE	1	0
2086	YJL145W	Agreement	TRUE	1	0.045
2087	YGL241W	No agreement	FALSE	1	0.125
2088	YBR131W	No agreement	TRUE	1	0
2089	YKL014C	Agreement	FALSE	0	0.063
2090	YPL088W	No agreement	TRUE	1	0
2091	YKL069W	No agreement	FALSE	1	0.014
2092	YPL269W	Agreement	FALSE	1	0.255
2093	YNR068C	No agreement	FALSE	1	0
2094	YKL007W	Agreement	FALSE	1	0.248
2095	YOR301W	Agreement	FALSE	1	0.004
2096	YJR015W	No agreement	FALSE	1	0.008
2097	YGL253W	Agreement	TRUE	1	0
2098	YPL012W	Agreement	FALSE	0	0.066
2099	YDR334W	Agreement	TRUE	1	0.019
2100	YOR116C	Agreement	FALSE	0	0.024
2101	YPR052C	No agreement	FALSE	1	0
2102	YDR538W	No agreement	FALSE	1	0.003

2103	YBR084W	Agreement	FALSE	1	0.043
2104	YNL320W	Agreement	FALSE	1	0.16
2105	YLR139C	No agreement	FALSE	1	0
2106	YDL168W	No agreement	TRUE	1	0
2107	YBR140C	Agreement	FALSE	0	0
2108	YBR121C	Agreement	FALSE	0	0
2109	YDL017W	No agreement	TRUE	0	0.003
2110	YNL096C	Agreement	FALSE	0	0
2111	YNL273W	Agreement	TRUE	1	0
2112	YDR427W	Agreement	TRUE	0	0.007
2113	YDR519W	No agreement	FALSE	1	0.046
2114	YAL014C	Agreement	TRUE	1	0.046
2115	YDR155C	Agreement	FALSE	1	0
2116	YPR171W	Agreement	FALSE	1	0.241
2117	YOL108C	Agreement	TRUE	1	0.019
2118	YPL178W	No agreement	TRUE	1	0.034
2119	YPL107W	Agreement	TRUE	1	0
2120	YDR096W	Agreement	TRUE	1	0
2121	YJL101C	Agreement	FALSE	1	0
2122	YLR262C	Agreement	FALSE	1	0.262
2123	YDR525W-A	Agreement	FALSE	1	0
2124	YEL025C	Agreement	TRUE	1	0.131
2125	YCR068W	No agreement	FALSE	1	0.083
2126	YPL082C	No agreement	TRUE	0	0.029
2127	YKL212W	No agreement	FALSE	1	0
2128	YPL023C	No agreement	FALSE	1	0.001
2129	YBR201W	No agreement	TRUE	1	0.127
2130	YBR125C	No agreement	FALSE	1	0
2131	YLL026W	Agreement	TRUE	1	0
2132	YOR307C	Agreement	FALSE	1	0.013
2133	YNR027W	No agreement	FALSE	1	0.061
2134	YJR036C	Agreement	FALSE	1	0
2135	YNL249C	Agreement	FALSE	1	0.01
2136	YER156C	Agreement	FALSE	1	0.003
2137	YOR051C	No agreement	TRUE	1	0.087
2138	YFL005W	No agreement	TRUE	0	0.033
2139	YNR056C	No agreement	FALSE	1	0
2140	YMR019W	Agreement	TRUE	1	0.002
2141	YBR177C	Agreement	FALSE	1	0.006
2142	YGL215W	No agreement	FALSE	1	0.22
2143	YLL010C	No agreement	FALSE	1	0.212
2144	YER120W	Agreement	FALSE	1	0.075
2145	YGR082W	No agreement	FALSE	0	0
2146	YBR159W	Agreement	FALSE	1	0
2147	YIL105C	Agreement	TRUE	1	0
2148	YDL233W	Agreement	FALSE	1	0
2149	YDR179W-A	No agreement	FALSE	1	0.266
2150	YLR150W	Agreement	TRUE	1	0
2151	YPL144W	No agreement	FALSE	1	0

2152	YKR100C	Agreement	FALSE	1	0.156
2153	YJL118W	No agreement	FALSE	1	0
2154	YDR180W	Agreement	FALSE	0	0.244
2155	YFL049W	No agreement	FALSE	1	0.172
2156	YDL132W	Agreement	TRUE	0	0
2157	YOR224C	Agreement	FALSE	0	0.012
2158	YOR081C	No agreement	FALSE	1	0.067
2159	YDL051W	Agreement	TRUE	1	0.02
2160	YGL057C	No agreement	TRUE	1	0.001
2161	YJL204C	No agreement	TRUE	1	0.273
2162	YHR020W	Agreement	FALSE	0	0
2163	YBR038W	Agreement	FALSE	0	0
2164	YGR076C	No agreement	TRUE	1	0
2165	YLR449W	Agreement	TRUE	1	0.023
2166	YDR089W	No agreement	FALSE	1	0.005
2167	YNL079C	Agreement	TRUE	1	0
2168	YOR389W	Agreement	TRUE	0	0
2169	YML062C	No agreement	TRUE	1	0.004
2170	YER165W	Agreement	FALSE	0	0
2171	YDL240W	Agreement	TRUE	1	0.005
2172	YGL207W	No agreement	FALSE	0	0.152
2173	YMR152W	Agreement	TRUE	1	0
2174	YIL149C	Agreement	TRUE	1	0.029
2175	YJL126W	Agreement	FALSE	1	0.023
2176	YPR072W	Agreement	TRUE	1	0.189
2177	YLR368W	Agreement	FALSE	1	0.141
2178	YBL030C	No agreement	FALSE	0	0
2179	YOR092W	Agreement	TRUE	1	0.015
2180	YKL114C	Agreement	TRUE	1	0.027
2181	YDR276C	Agreement	FALSE	1	0
2182	YER019W	No agreement	FALSE	1	0
2183	YLR118C	Agreement	FALSE	1	0.191
2184	YLR342W	No agreement	FALSE	1	0
2185	YGL080W	Agreement	TRUE	1	0
2186	YDR217C	Agreement	TRUE	1	0.011
2187	YOR188W	Agreement	TRUE	1	0
2188	YFL024C	Agreement	TRUE	0	0
2189	YFR049W	Agreement	TRUE	1	0
2190	YKL135C	Agreement	FALSE	1	0.16
2191	YJR126C	Agreement	FALSE	1	0
2192	YJL141C	Agreement	TRUE	1	0
2193	YNL040W	No agreement	FALSE	1	0.011
2194	YPR125W	No agreement	TRUE	1	0.001
2195	YHR110W	Agreement	TRUE	1	0.044
2196	YMR187C	No agreement	FALSE	1	0.017
2197	YMR289W	Agreement	FALSE	1	0.192
2198	YOR280C	No agreement	FALSE	1	0
2199	YLR275W	No agreement	TRUE	0	0.044
2200	YNR001C	Agreement	FALSE	1	0

2201	YPL083C	No agreement	FALSE	0	0
2202	YKL020C	Agreement	TRUE	1	0
2203	YOR124C	Agreement	TRUE	1	0.074
2204	YML061C	Agreement	TRUE	1	0
2205	YJR040W	Agreement	FALSE	1	0.219
2206	YPL141C	Agreement	TRUE	1	0
2207	YNL186W	Agreement	TRUE	0	0.056
2208	YPL237W	Agreement	TRUE	0	0.004
2209	YLR117C	Agreement	TRUE	0	0
2210	YJL117W	Agreement	TRUE	1	0.214
2211	YCR020C-A	No agreement	FALSE	1	0.004
2212	YDL013W	No agreement	TRUE	1	0
2213	YDR023W	Agreement	FALSE	0	0
2214	YKR027W	Agreement	FALSE	1	0.026
2215	YCR027C	No agreement	FALSE	1	0.018
2216	YOR375C	Agreement	FALSE	1	0.164
2217	YGR147C	No agreement	FALSE	0	0
2218	YBR196C	Agreement	TRUE	0	0.017
2219	YER155C	Agreement	FALSE	1	0.28
2220	YJL163C	Agreement	TRUE	1	0
2221	YOR076C	Agreement	TRUE	1	0.053
2222	YJL208C	Agreement	TRUE	1	0
2223	YOL137W	No agreement	FALSE	1	0.094
2224	YPR137W	Agreement	TRUE	0	0.032
2225	YJL184W	No agreement	FALSE	1	0.168
2226	YLR210W	No agreement	TRUE	1	0.048
2227	YBL076C	No agreement	TRUE	0	0.006
2228	YPL217C	Agreement	TRUE	0	0.048
2229	YOR208W	Agreement	FALSE	1	0
2230	YBL112C	No agreement	TRUE	0	0
2231	YOR103C	No agreement	FALSE	0	0.182
2232	YBL034C	Agreement	FALSE	0	0
2233	YLR372W	Agreement	FALSE	1	0
2234	YPL239W	Agreement	TRUE	1	0.006
2235	YHR155W	Agreement	FALSE	1	0.139
2236	YPL053C	No agreement	TRUE	1	0.001
2237	YOL129W	Agreement	FALSE	1	0
2238	YOR285W	Agreement	FALSE	1	0
2239	YFR028C	Agreement	FALSE	0	0.022
2240	YFR040W	No agreement	FALSE	1	0.183
2241	YPR144C	Agreement	FALSE	0	0.098
2242	YHR026W	No agreement	TRUE	1	0.004
2243	YDR135C	No agreement	FALSE	1	0.001
2244	YIL154C	No agreement	TRUE	1	0
2245	YGR218W	No agreement	TRUE	0	0.036
2246	YOR209C	Agreement	TRUE	1	0
2247	YLR264W	Agreement	FALSE	1	0
2248	YHR144C	Agreement	TRUE	0	0
2249	YLR393W	No agreement	FALSE	1	0

2250	YGL077C	Agreement	FALSE	1	0.176
2251	YML002W	Agreement	TRUE	1	0
2252	YDL092W	No agreement	FALSE	0	0.006
2253	YDR045C	Agreement	TRUE	0	0.02
2254	YOR316C	No agreement	FALSE	1	0.2
2255	YOR356W	Agreement	TRUE	1	0
2256	YDL101C	Agreement	TRUE	1	0
2257	YLR288C	Agreement	TRUE	1	0.001
2258	YOL056W	No agreement	TRUE	1	0
2259	YFR002W	No agreement	TRUE	0	0.167
2260	YLR248W	No agreement	FALSE	1	0.192
2261	YML009C	No agreement	FALSE	1	0
2262	YBL052C	No agreement	TRUE	1	0.003
2263	YLR350W	No agreement	TRUE	1	0.001
2264	YER075C	No agreement	FALSE	1	0.171
2265	YDR178W	No agreement	TRUE	1	0
2266	YKR084C	No agreement	TRUE	1	0
2267	YLR093C	Agreement	TRUE	1	0
2268	YHR111W	No agreement	FALSE	1	0.256
2269	YLR046C	Agreement	FALSE	1	0.032
2270	YPL236C	Agreement	FALSE	1	0
2271	YPL190C	Agreement	TRUE	0	0.009
2272	YMR292W	No agreement	FALSE	1	0
2273	YKL179C	No agreement	FALSE	1	0.142
2274	YDR092W	No agreement	FALSE	1	0.141
2275	YGR128C	Agreement	FALSE	0	0.057
2276	YDR153C	No agreement	TRUE	1	0
2277	YGR157W	Agreement	FALSE	1	0
2278	YOR142W	Agreement	TRUE	1	0
2279	YNL112W	Agreement	TRUE	0	0.049
2280	YER170W	Agreement	FALSE	1	0
2281	YDL180W	No agreement	FALSE	1	0
2282	YJR041C	Agreement	FALSE	0	0.017
2283	YEL016C	No agreement	FALSE	1	0
2284	YBR088C	Agreement	TRUE	0	0
2285	YDR032C	Agreement	TRUE	1	0
2286	YML031W	No agreement	FALSE	0	0.245
2287	YLR292C	No agreement	TRUE	1	0.011
2288	YNL288W	No agreement	FALSE	1	0.004
2289	YDR159W	No agreement	TRUE	1	0.081
2290	YDR130C	Agreement	TRUE	1	0
2291	YDR028C	No agreement	TRUE	1	0.001
2292	YKR101W	Agreement	FALSE	1	0.253
2293	YGR007W	No agreement	FALSE	1	0.002
2294	YLR336C	Agreement	FALSE	0	0.066
2295	YER166W	No agreement	FALSE	1	0.137
2296	YCR084C	No agreement	FALSE	1	0.034
2297	YPL054W	Agreement	FALSE	1	0
2298	YNR021W	Agreement	FALSE	1	0.114

2299	YOR039W	No agreement	FALSE	1	0.031
2300	YBR199W	Agreement	FALSE	1	0.042
2301	YPL258C	No agreement	TRUE	1	0
2302	YHR036W	No agreement	FALSE	0	0.269
2303	YCR031C	Agreement	FALSE	1	0.011
2304	YGL076C	Agreement	FALSE	1	0
2305	YJL073W	Agreement	TRUE	1	0
2306	YPR020W	No agreement	TRUE	1	0
2307	YGL226C-A	No agreement	TRUE	1	0.006
2308	YOR021C	No agreement	FALSE	1	0.164
2309	YGR056W	No agreement	FALSE	1	0.111
2310	YJL166W	Agreement	FALSE	1	0.178
2311	YPR110C	Agreement	FALSE	0	0.046
2312	YDR299W	Agreement	FALSE	0	0.058
2313	YKR011C	Agreement	TRUE	1	0.052
2314	YLR268W	No agreement	TRUE	1	0
2315	YEL013W	Agreement	FALSE	1	0
2316	YPR016C	Agreement	FALSE	0	0.026
2317	YPL177C	No agreement	FALSE	1	0
2318	YMR029C	Agreement	TRUE	1	0
2319	YMR099C	Agreement	TRUE	1	0
2320	YJR098C	Agreement	FALSE	1	0
2321	YDL006W	Agreement	TRUE	1	0.014
2322	YKR068C	No agreement	FALSE	0	0.018
2323	YEL054C	Agreement	FALSE	1	0
2324	YKL077W	No agreement	FALSE	1	0.238
2325	YER122C	No agreement	TRUE	1	0
2326	YDR208W	Agreement	TRUE	0	0.004
2327	YGR003W	No agreement	FALSE	1	0.042
2328	YPL078C	No agreement	FALSE	1	0
2329	YMR078C	Agreement	TRUE	1	0
2330	YGL191W	Agreement	TRUE	0	0
2331	YOL027C	Agreement	TRUE	1	0.1
2332	YKL154W	No agreement	FALSE	0	0.031
2333	YJR030C	Agreement	TRUE	1	0
2334	YJL044C	Agreement	FALSE	1	0.001
2335	YGL130W	No agreement	TRUE	0	0.01
2336	YGL111W	Agreement	TRUE	0	0.024
2337	YGR097W	No agreement	FALSE	1	0
2338	YEL060C	Agreement	FALSE	1	0
2339	YJR143C	No agreement	FALSE	0	0
2340	YDL214C	Agreement	FALSE	1	0
2341	YCL043C	No agreement	FALSE	0	0.278
2342	YDL138W	No agreement	FALSE	1	0.001
2343	YJR139C	Agreement	TRUE	1	0.013
2344	YIL155C	Agreement	TRUE	1	0
2345	YBR274W	Agreement	FALSE	1	0.018
2346	YBL014C	No agreement	FALSE	0	0.271
2347	YGR009C	No agreement	FALSE	0	0.291

2348	YJL134W	No agreement	TRUE	1	0.006
2349	YLR293C	Agreement	FALSE	1	0.001
2350	YGL049C	No agreement	TRUE	1	0.001
2351	YER171W	No agreement	TRUE	0	0.05
2352	YEL037C	No agreement	TRUE	1	0.004
2353	YGL229C	Agreement	TRUE	1	0
2354	YER180C	No agreement	TRUE	1	0.105
2355	YGR241C	Agreement	TRUE	1	0
2356	YER026C	Agreement	FALSE	0	0
2357	YDR333C	No agreement	FALSE	1	0.091
2358	YLR115W	Agreement	FALSE	0	0.097
2359	YBL066C	No agreement	FALSE	1	0.007
2360	YJL047C	No agreement	TRUE	1	0.001
2361	YNL262W	Agreement	TRUE	0	0
2362	YDL073W	No agreement	FALSE	1	0.121
2363	YNR017W	No agreement	TRUE	0	0
2364	YKR063C	No agreement	TRUE	0	0.001
2365	YOR089C	No agreement	FALSE	1	0.102
2366	YPL022W	No agreement	FALSE	1	0.044
2367	YGL196W	No agreement	FALSE	1	0
2368	YFL016C	Agreement	FALSE	0	0.23
2369	YIL067C	No agreement	FALSE	1	0.064
2370	YJR067C	No agreement	TRUE	0	0
2371	YJR125C	Agreement	TRUE	1	0.02
2372	YPL050C	No agreement	TRUE	1	0.009
2373	YGR245C	Agreement	TRUE	0	0.035
2374	YDR224C	Agreement	FALSE	0	0
2375	YDL137W	Agreement	FALSE	1	0
2376	YMR242C	Agreement	FALSE	1	0.217
2377	YGL246C	No agreement	TRUE	1	0.026
2378	YMR030W	Agreement	TRUE	1	0
2379	YDR331W	No agreement	TRUE	0	0
2380	YPR048W	No agreement	FALSE	0	0.015
2381	YDR216W	Agreement	FALSE	1	0
2382	YDL084W	No agreement	FALSE	0	0.246
2383	YNR011C	No agreement	FALSE	0	0.226
2384	YDR249C	No agreement	FALSE	1	0.004
2385	YKL184W	No agreement	FALSE	1	0.002
2386	YNR026C	Agreement	TRUE	0	0.058
2387	YDR235W	No agreement	FALSE	0	0
2388	YLR094C	No agreement	TRUE	1	0.019
2389	YAL021C	Agreement	FALSE	1	0.134
2390	YNL232W	Agreement	TRUE	0	0.134
2391	YJR064W	Agreement	TRUE	0	0.165
2392	YIL036W	Agreement	TRUE	1	0
2393	YGL223C	No agreement	FALSE	1	0.263
2394	YEL036C	No agreement	TRUE	1	0.017
2395	YGR015C	Agreement	FALSE	1	0.001
2396	YNR008W	Agreement	TRUE	1	0.001

2397	YLR429W	Agreement	TRUE	1	0
2398	YMR203W	Agreement	FALSE	0	0
2399	YER167W	No agreement	TRUE	1	0
2400	YJR045C	No agreement	FALSE	0	0
2401	YDL069C	No agreement	FALSE	1	0
2402	YDR398W	Agreement	FALSE	0	0.125
2403	YGR262C	No agreement	TRUE	1	0.001
2404	YGR090W	Agreement	FALSE	0	0.003
2405	YNL229C	No agreement	FALSE	1	0.285
2406	YPL274W	Agreement	FALSE	1	0
2407	YMR156C	Agreement	TRUE	1	0
2408	YIL049W	Agreement	TRUE	1	0.049
2409	YOR322C	No agreement	TRUE	1	0.045
2410	YGR238C	Agreement	TRUE	1	0
2411	YNL263C	No agreement	FALSE	0	0.013
2412	YGL062W	No agreement	FALSE	1	0
2413	YKR044W	No agreement	FALSE	1	0.138
2414	YDR101C	Agreement	FALSE	1	0.057
2415	YGR270W	Agreement	FALSE	1	0.189
2416	YER005W	No agreement	FALSE	1	0.002
2417	YAR008W	Agreement	TRUE	0	0
2418	YGL022W	No agreement	FALSE	0	0
2419	YKL157W	Agreement	FALSE	1	0.18
2420	YOR172W	Agreement	FALSE	1	0.246
2421	YFL007W	Agreement	FALSE	0	0
2422	YEL058W	No agreement	TRUE	0	0.037
2423	YHR010W	Agreement	FALSE	1	0
2424	YDR534C	No agreement	FALSE	1	0
2425	YGL150C	No agreement	TRUE	0	0.006
2426	YLR133W	Agreement	FALSE	1	0
2427	YBR136W	Agreement	FALSE	0	0
2428	YPR055W	Agreement	FALSE	0	0.003
2429	YKL203C	Agreement	TRUE	0	0.005
2430	YMR120C	Agreement	FALSE	1	0
2431	YPR057W	No agreement	TRUE	1	0
2432	YER083C	No agreement	FALSE	1	0.033
2433	YLR010C	Agreement	FALSE	0	0
2434	YMR125W	No agreement	FALSE	1	0.196
2435	YGL060W	Agreement	FALSE	1	0.134
2436	YGL003C	Agreement	TRUE	1	0.015
2437	YOR138C	Agreement	TRUE	1	0.1
2438	YIR028W	No agreement	FALSE	1	0.023
2439	YLR456W	Agreement	FALSE	1	0.21
2440	YIL143C	No agreement	TRUE	0	0.042
2441	YGR080W	Agreement	FALSE	1	0.26
2442	YMR241W	Agreement	FALSE	1	0
2443	YBR249C	No agreement	FALSE	1	0
2444	YGL167C	Agreement	FALSE	1	0.163
2445	YDR457W	Agreement	FALSE	1	0.031

2446	YPR107C	Agreement	FALSE	0	0.006
2447	YKL173W	No agreement	TRUE	0	0.022
2448	YBR042C	Agreement	FALSE	1	0.21
2449	YKR070W	No agreement	TRUE	1	0.023
2450	YCR028C	No agreement	FALSE	1	0.025
2451	YKL033W-A	No agreement	FALSE	1	0.256
2452	YMR305C	No agreement	FALSE	1	0
2453	YJL159W	No agreement	FALSE	1	0.004
2454	YJR110W	Agreement	TRUE	1	0.003
2455	YOR145C	Agreement	FALSE	0	0.014
2456	YGL103W	Agreement	FALSE	0	0.002
2457	YHR082C	Agreement	FALSE	1	0.105
2458	YHR161C	No agreement	TRUE	1	0
2459	YNL163C	Agreement	TRUE	0	0.081
2460	YBL011W	Agreement	FALSE	1	0.001
2461	YJR002W	Agreement	TRUE	0	0.017
2462	YGR174C	Agreement	FALSE	1	0
2463	YBR019C	Agreement	FALSE	1	0.052
2464	YLR309C	No agreement	TRUE	1	0.008
2465	YAL009W	No agreement	TRUE	1	0.065
2466	YGL097W	Agreement	TRUE	0	0
2467	YPR026W	Agreement	FALSE	1	0
2468	YBR264C	Agreement	TRUE	1	0.01
2469	YOR109W	No agreement	FALSE	1	0.133
2470	YHR061C	Agreement	FALSE	1	0
2471	YDR300C	Agreement	TRUE	1	0
2472	YJL171C	No agreement	FALSE	1	0.258
2473	YJL090C	No agreement	FALSE	0	0.244
2474	YER074W	Agreement	FALSE	1	0
2475	YHR107C	No agreement	TRUE	0	0
2476	YKL082C	Agreement	TRUE	0	0.099
2477	YPL086C	Agreement	FALSE	1	0.012
2478	YBR165W	Agreement	FALSE	1	0.005
2479	YGR134W	No agreement	FALSE	1	0.018
2480	YMR073C	Agreement	FALSE	1	0.18
2481	YCL002C	No agreement	FALSE	1	0.072
2482	YNR065C	No agreement	FALSE	1	0.099
2483	YDR087C	Agreement	TRUE	0	0.091
2484	YNL327W	No agreement	FALSE	1	0
2485	YHR080C	Agreement	FALSE	1	0
2486	YJL099W	No agreement	FALSE	1	0.034
2487	YER173W	Agreement	TRUE	1	0.157
2488	YLR203C	No agreement	FALSE	1	0
2489	YGR027C	Agreement	FALSE	1	0.002
2490	YBR220C	No agreement	FALSE	1	0
2491	YIL008W	No agreement	TRUE	1	0.089
2492	YDR158W	Agreement	FALSE	1	0
2493	YIL152W	Agreement	TRUE	1	0
2494	YLL036C	No agreement	TRUE	0	0.073

2495	YPL014W	Agreement	FALSE	1	0.056
2496	YDR428C	No agreement	FALSE	1	0
2497	YGR167W	Agreement	TRUE	1	0.002
2498	YBR302C	Agreement	TRUE	0	0
2499	YHR193C	Agreement	FALSE	1	0.21
2500	YHR042W	No agreement	FALSE	0	0.002
2501	YLR095C	No agreement	FALSE	1	0.09
2502	YCR082W	Agreement	FALSE	1	0.001
2503	YLR277C	No agreement	TRUE	0	0.136
2504	YNL230C	Agreement	TRUE	1	0
2505	YNL239W	Agreement	FALSE	1	0
2506	YIL048W	No agreement	FALSE	0	0.202
2507	YGL094C	No agreement	TRUE	1	0.017
2508	YBR211C	No agreement	FALSE	0	0.232
2509	YKL015W	No agreement	FALSE	1	0
2510	YDR086C	Agreement	FALSE	0	0.125
2511	YMR143W	Agreement	FALSE	1	0.004
2512	YGL093W	No agreement	TRUE	0	0.016
2513	YLR373C	Agreement	TRUE	1	0
2514	YGR255C	Agreement	FALSE	0	0.001
2515	YGL113W	No agreement	FALSE	0	0.225
2516	YLR190W	Agreement	FALSE	1	0
2517	YLR243W	Agreement	TRUE	0	0.069
2518	YGL210W	Agreement	TRUE	1	0.128
2519	YJR032W	No agreement	FALSE	1	0.203
2520	YPR181C	Agreement	FALSE	0	0.003
2521	YNR006W	No agreement	TRUE	1	0.009
2522	YML032C	Agreement	FALSE	1	0.083
2523	YGR276C	No agreement	FALSE	0	0.209
2524	YOR085W	Agreement	FALSE	1	0.069
2525	YCR032W	No agreement	TRUE	1	0.025
2526	YOL020W	No agreement	FALSE	1	0
2527	YHR052W	Agreement	FALSE	0	0.056
2528	YDL063C	Agreement	FALSE	1	0.071
2529	YPL032C	No agreement	FALSE	1	0
2530	YER025W	Agreement	FALSE	0	0.006
2531	YDL127W	Agreement	TRUE	1	0.025
2532	YKL110C	Agreement	FALSE	1	0.085
2533	YNL316C	No agreement	FALSE	1	0.002
2534	YLR278C	No agreement	FALSE	1	0.124
2535	YOL154W	No agreement	TRUE	0	0
2536	YBL026W	No agreement	FALSE	0	0.007
2537	YJL046W	Agreement	TRUE	1	0
2538	YCR054C	Agreement	FALSE	0	0.094
2539	YOR160W	Agreement	FALSE	0	0.183
2540	YGR061C	Agreement	FALSE	1	0
2541	YDR517W	No agreement	FALSE	1	0.015
2542	YPL108W	No agreement	TRUE	1	0.04
2543	YOR110W	No agreement	TRUE	0	0.022

2544	YFL025C	Agreement	FALSE	1	0
2545	YLR406C	Agreement	TRUE	1	0
2546	YDL226C	Agreement	TRUE	1	0.001
2547	YDR069C	Agreement	TRUE	1	0.001
2548	YGR252W	No agreement	TRUE	0	0.055
2549	YBR255W	Agreement	TRUE	1	0
2550	YMR261C	Agreement	FALSE	1	0
2551	YML095C	No agreement	FALSE	1	0.175
2552	YOR297C	No agreement	FALSE	1	0.008
2553	YDR408C	Agreement	FALSE	1	0.001
2554	YDR298C	No agreement	FALSE	1	0
2555	YCR005C	Agreement	FALSE	1	0
2556	YCL034W	Agreement	TRUE	1	0.049
2557	YLR067C	No agreement	TRUE	1	0
2558	YOL105C	Agreement	FALSE	1	0
2559	YER093C	No agreement	FALSE	0	0.225
2560	YDL081C	Agreement	TRUE	1	0.002
2561	YGR084C	No agreement	TRUE	1	0
2562	YOL018C	Agreement	TRUE	1	0
2563	YIL114C	No agreement	FALSE	1	0.149
2564	YBL092W	Agreement	TRUE	0	0
2565	YLL021W	Agreement	TRUE	1	0
2566	YGR136W	No agreement	FALSE	1	0.118
2567	YDL178W	No agreement	TRUE	1	0
2568	YHR039C	No agreement	FALSE	1	0
2569	YPR159W	Agreement	FALSE	1	0
2570	YHL039W	No agreement	FALSE	1	0.056
2571	YGL017W	No agreement	FALSE	1	0
2572	YNL083W	No agreement	TRUE	1	0
2573	YMR198W	Agreement	FALSE	1	0
2574	YML102W	Agreement	TRUE	1	0
2575	YFL036W	No agreement	FALSE	1	0
2576	YLR347C	Agreement	FALSE	0	0.008
2577	YPL137C	Agreement	FALSE	1	0.097
2578	YHR070W	Agreement	TRUE	0	0.053
2579	YER161C	Agreement	FALSE	1	0.065
2580	YHR170W	Agreement	FALSE	0	0.059
2581	YJL133W	Agreement	FALSE	1	0.001
2582	YPL243W	Agreement	TRUE	0	0.004
2583	YCL001W	No agreement	FALSE	1	0.037
2584	YLR301W	No agreement	FALSE	0	0.102
2585	YNR024W	Agreement	TRUE	1	0.259
2586	YIL145C	No agreement	TRUE	1	0.008
2587	YAR002W	Agreement	FALSE	1	0.107
2588	YKL079W	Agreement	FALSE	1	0.052
2589	YMR098C	No agreement	TRUE	1	0
2590	YPR091C	Agreement	TRUE	1	0
2591	YML088W	Agreement	TRUE	1	0
2592	YDR035W	No agreement	TRUE	1	0

2593	YBL015W	Agreement	FALSE	1	0
2594	YDR441C	No agreement	FALSE	1	0.002
2595	YOR219C	Agreement	FALSE	1	0.001
2596	YGL244W	No agreement	TRUE	1	0.219
2597	YER043C	No agreement	FALSE	0	0
2598	YER036C	Agreement	FALSE	0	0.024
2599	YOL127W	Agreement	FALSE	0	0
2600	YNL090W	Agreement	TRUE	1	0.007
2601	YKR095W	No agreement	TRUE	1	0.019
2602	YPL183C	Agreement	FALSE	1	0.048
2603	YGR057C	No agreement	FALSE	1	0.229
2604	YAL030W	Agreement	FALSE	1	0
2605	YLR238W	Agreement	FALSE	1	0.2
2606	YOR373W	No agreement	FALSE	0	0
2607	YOR324C	Agreement	TRUE	1	0
2608	YIL088C	No agreement	FALSE	1	0.114
2609	YMR293C	No agreement	FALSE	1	0
2610	YOR067C	No agreement	FALSE	1	0.044
2611	YGL038C	No agreement	FALSE	1	0.214
2612	YOR241W	No agreement	TRUE	1	0.001
2613	YPR079W	No agreement	TRUE	1	0.008
2614	YPL230W	Agreement	TRUE	1	0
2615	YJR090C	Agreement	FALSE	1	0.225
2616	YNL292W	Agreement	TRUE	1	0.003
2617	YHR142W	No agreement	TRUE	1	0.002
2618	YHR078W	Agreement	FALSE	1	0.168
2619	YBR286W	Agreement	FALSE	1	0
2620	YNL123W	Agreement	FALSE	1	0.039
2621	YDL185W	Agreement	FALSE	1	0.089
2622	YGL197W	Agreement	TRUE	1	0.001
2623	YHR005C-A	No agreement	TRUE	1	0
2624	YKL021C	Agreement	TRUE	0	0.042
2625	YGL137W	No agreement	TRUE	0	0
2626	YPR032W	Agreement	FALSE	1	0.05
2627	YJL124C	No agreement	TRUE	1	0
2628	YER151C	No agreement	FALSE	1	0.289
2629	YOL102C	No agreement	FALSE	0	0.184
2630	YEL029C	No agreement	FALSE	1	0.001
2631	YDR258C	No agreement	TRUE	1	0.006
2632	YGL071W	No agreement	TRUE	1	0
2633	YMR012W	No agreement	TRUE	1	0
2634	YHR128W	Agreement	FALSE	0	0.014
2635	YER003C	No agreement	FALSE	0	0
2636	YDR363W-A	Agreement	TRUE	1	0.014
2637	YNL247W	Agreement	TRUE	0	0.003
2638	YOR353C	Agreement	TRUE	0	0.017
2639	YGR063C	No agreement	FALSE	1	0.09
2640	YOR147W	Agreement	FALSE	1	0.032
2641	YHR133C	No agreement	FALSE	1	0.003

2642	YOR198C	Agreement	TRUE	1	0
2643	YOL068C	No agreement	FALSE	1	0.105
2644	YIL056W	Agreement	FALSE	0	0.019
2645	YHR046C	Agreement	FALSE	1	0.022
2646	YDR429C	Agreement	FALSE	0	0.001
2647	YDR472W	No agreement	FALSE	0	0
2648	YLR193C	Agreement	TRUE	1	0.001
2649	YOR278W	No agreement	TRUE	0	0.001
2650	YEL040W	Agreement	FALSE	1	0
2651	YER168C	No agreement	TRUE	0	0
2652	YGR096W	No agreement	FALSE	1	0.106
2653	YER146W	No agreement	TRUE	0	0.074
2654	YIL047C	No agreement	FALSE	1	0.18
2655	YER113C	Agreement	FALSE	1	0.001
2656	YNL121C	No agreement	TRUE	1	0
2657	YLR305C	No agreement	FALSE	0	0.095
2658	YIL135C	No agreement	FALSE	1	0.018
2659	YOL063C	Agreement	TRUE	1	0.2
2660	YHR062C	No agreement	TRUE	0	0.046
2661	YER128W	No agreement	TRUE	1	0.036
2662	YOL052C	No agreement	FALSE	1	0
2663	YLR079W	No agreement	TRUE	1	0
2664	YHR024C	Agreement	FALSE	0	0
2665	YGR207C	No agreement	TRUE	1	0
2666	YLR226W	No agreement	TRUE	1	0.015
2667	YOR299W	No agreement	FALSE	1	0
2668	YDR073W	Agreement	TRUE	1	0
2669	YML021C	Agreement	TRUE	1	0
2670	YJR024C	Agreement	FALSE	1	0.074
2671	YOR154W	No agreement	TRUE	1	0.097
2672	YPL085W	No agreement	TRUE	0	0.057
2673	YJR124C	Agreement	FALSE	1	0
2674	YPL263C	Agreement	TRUE	1	0.044
2675	YDL093W	No agreement	FALSE	1	0.001
2676	YMR204C	Agreement	FALSE	1	0.19
2677	YOL022C	Agreement	TRUE	0	0.033
2678	YHL025W	No agreement	FALSE	1	0.145
2679	YOL090W	Agreement	TRUE	1	0
2680	YDR395W	Agreement	FALSE	1	0.003
2681	YGR274C	No agreement	TRUE	0	0.075
2682	YBR290W	Agreement	TRUE	1	0.086
2683	YEL021W	No agreement	FALSE	0	0.083
2684	YGL044C	Agreement	TRUE	0	0.027
2685	YMR178W	Agreement	TRUE	1	0.002
2686	YEL042W	No agreement	FALSE	1	0.004
2687	YAL015C	Agreement	TRUE	1	0.254
2688	YMR171C	No agreement	FALSE	1	0.226
2689	YGR026W	Agreement	FALSE	1	0.033
2690	YAR033W	Agreement	FALSE	0	0.07

2691	YBR023C	Agreement	FALSE	1	0.071
2692	YFR047C	Agreement	FALSE	1	0.275
2693	YBR189W	Agreement	FALSE	1	0
2694	YDR194C	Agreement	TRUE	1	0
2695	YGL245W	Agreement	FALSE	0	0.046
2696	YML064C	Agreement	TRUE	0	0.015
2697	YLR459W	Agreement	FALSE	0	0.093
2698	YOL081W	Agreement	FALSE	1	0
2699	YGL055W	No agreement	FALSE	0	0.034
2700	YMR076C	Agreement	TRUE	0	0
2701	YDL064W	Agreement	TRUE	0	0.003
2702	YGL025C	No agreement	FALSE	1	0.17
2703	YGL008C	No agreement	FALSE	0	0.002
2704	YGL242C	Agreement	TRUE	1	0.182
2705	YHR068W	Agreement	FALSE	0	0
2706	YOR098C	No agreement	FALSE	0	0.156
2707	YPL060W	Agreement	FALSE	1	0.117
2708	YDR508C	Agreement	FALSE	1	0.222
2709	YDR099W	Agreement	TRUE	1	0
2710	YLR325C	Agreement	FALSE	1	0.001
2711	YGR033C	No agreement	TRUE	1	0
2712	YNL056W	No agreement	TRUE	1	0.001
2713	YGR140W	No agreement	TRUE	0	0
2714	YIL151C	No agreement	FALSE	0	0.262
2715	YMR196W	Agreement	TRUE	1	0
2716	YGR285C	No agreement	FALSE	1	0.002
2717	YOR007C	No agreement	TRUE	1	0
2718	YBL072C	Agreement	FALSE	1	0.002
2719	YOR330C	Agreement	TRUE	1	0.004
2720	YLR407W	No agreement	TRUE	1	0.044
2721	YPR133C	Agreement	TRUE	0	0.012
2722	YLR132C	Agreement	FALSE	0	0
2723	YOR156C	No agreement	FALSE	1	0.167
2724	YER048W-A	Agreement	FALSE	0	0.001
2725	YML096W	Agreement	FALSE	1	0.001
2726	YOR361C	Agreement	FALSE	0	0
2727	YER048C	Agreement	TRUE	1	0
2728	YOR171C	No agreement	FALSE	1	0.127
2729	YBR029C	Agreement	FALSE	0	0
2730	YER066W	Agreement	FALSE	1	0
2731	YBR014C	No agreement	FALSE	1	0.238
2732	YIL128W	Agreement	FALSE	1	0.004
2733	YOL098C	No agreement	FALSE	1	0.007
2734	YJR075W	No agreement	TRUE	1	0.041
2735	YDL143W	No agreement	FALSE	0	0.064
2736	YNL222W	Agreement	FALSE	0	0.005
2737	YCL009C	No agreement	TRUE	1	0.223
2738	YGR243W	No agreement	FALSE	1	0
2739	YIL130W	Agreement	TRUE	1	0.072

2740	YDR477W	Agreement	TRUE	1	0
2741	YLR048W	Agreement	FALSE	1	0
2742	YML023C	Agreement	FALSE	0	0.065
2743	YPR042C	Agreement	FALSE	1	0
2744	YNR016C	No agreement	TRUE	0	0.017
2745	YJL034W	No agreement	FALSE	0	0
2746	YLR397C	Agreement	FALSE	0	0.132
2747	YCR059C	No agreement	TRUE	1	0
2748	YNL113W	Agreement	FALSE	0	0.048
2749	YPL233W	No agreement	FALSE	0	0.026
2750	YHL027W	No agreement	FALSE	1	0.018
2751	YBR031W	Agreement	FALSE	1	0
2752	YOR374W	Agreement	TRUE	1	0
2753	YER095W	No agreement	FALSE	1	0.062
2754	YNL016W	No agreement	FALSE	1	0
2755	YDR425W	Agreement	FALSE	1	0
2756	YMR009W	No agreement	FALSE	1	0.002
2757	YGL220W	No agreement	FALSE	1	0.049
2758	YDL195W	Agreement	FALSE	0	0.068
2759	YDL144C	Agreement	TRUE	1	0.159
2760	YPR182W	Agreement	FALSE	0	0
2761	YGR279C	No agreement	TRUE	1	0
2762	YDL103C	Agreement	TRUE	0	0
2763	YGR267C	No agreement	FALSE	0	0.024
2764	YML013W	Agreement	TRUE	1	0.227
2765	YDR167W	No agreement	TRUE	0	0.002
2766	YMR310C	Agreement	FALSE	1	0.014
2767	YDR432W	Agreement	TRUE	1	0
2768	YBL091C	No agreement	FALSE	1	0.098
2769	YBR162W-A	Agreement	TRUE	1	0
2770	YPR056W	No agreement	TRUE	0	0.037
2771	YLL014W	Agreement	FALSE	1	0.008
2772	YLR304C	Agreement	FALSE	1	0
2773	YMR064W	Agreement	TRUE	1	0.001
2774	YMR247C	No agreement	FALSE	1	0.03
2775	YPL005W	Agreement	TRUE	1	0.001
2776	YPR083W	Agreement	FALSE	0	0.176
2777	YJL178C	Agreement	TRUE	1	0.002
2778	YNL326C	No agreement	TRUE	1	0.001
2779	YLR145W	No agreement	FALSE	0	0.142
2780	YDL036C	No agreement	TRUE	1	0.06
2781	YML091C	No agreement	TRUE	0	0
2782	YCR057C	Agreement	FALSE	0	0.099
2783	YER144C	No agreement	FALSE	1	0
2784	YJL209W	No agreement	FALSE	1	0.19
2785	YIR002C	No agreement	TRUE	1	0.046
2786	YER040W	No agreement	FALSE	1	0.042
2787	YOL006C	No agreement	FALSE	1	0.22
2788	YOR119C	No agreement	TRUE	0	0.082

2789	YFR044C	Agreement	FALSE	1	0.001
2790	YOR232W	No agreement	TRUE	0	0
2791	YGR102C	Agreement	TRUE	1	0.038
2792	YKL185W	No agreement	FALSE	1	0
2793	YHR088W	Agreement	TRUE	0	0.023
2794	YNL213C	No agreement	TRUE	1	0
2795	YGR065C	No agreement	TRUE	0	0
2796	YNR020C	No agreement	TRUE	1	0
2797	YAL023C	Agreement	FALSE	1	0
2798	YBR182C	No agreement	TRUE	1	0.143
2799	YDR388W	Agreement	TRUE	1	0
2800	YLR310C	Agreement	FALSE	0	0.128
2801	YNL283C	No agreement	FALSE	1	0
2802	YOR008C	Agreement	TRUE	1	0.002
2803	YLR422W	Agreement	FALSE	1	0
2804	YML081W	Agreement	FALSE	1	0.046
2805	YDR370C	No agreement	FALSE	1	0.165
2806	YGR058W	No agreement	TRUE	1	0.018
2807	YDR066C	No agreement	FALSE	1	0.006
2808	YMR260C	Agreement	TRUE	0	0.002
2809	YOL078W	No agreement	TRUE	0	0.027
2810	YDL243C	No agreement	FALSE	1	0
2811	YBR237W	Agreement	TRUE	0	0.037
2812	YFL002C	Agreement	TRUE	0	0.013
2813	YDL191W	Agreement	TRUE	1	0
2814	YLR002C	Agreement	FALSE	0	0.076
2815	YML086C	No agreement	FALSE	1	0.052
2816	YJL173C	Agreement	FALSE	0	0.05
2817	YDL135C	Agreement	TRUE	1	0
2818	YDR006C	Agreement	TRUE	1	0.077
2819	YDR062W	No agreement	FALSE	0	0
2820	YOR187W	No agreement	FALSE	1	0
2821	YGR173W	Agreement	TRUE	1	0
2822	YBR004C	Agreement	FALSE	0	0.002
2823	YLL002W	Agreement	TRUE	1	0
2824	YLR378C	Agreement	FALSE	0	0.092
2825	YAL046C	No agreement	FALSE	1	0
2826	YBL085W	Agreement	TRUE	1	0.008
2827	YGR264C	Agreement	TRUE	0	0.002
2828	YBR164C	Agreement	TRUE	1	0.002
2829	YBR279W	Agreement	TRUE	1	0.001
2830	YDL099W	Agreement	TRUE	1	0.002
2831	YML057W	Agreement	FALSE	1	0.013
2832	YPL042C	No agreement	FALSE	1	0.076
2833	YGR041W	No agreement	FALSE	1	0.001
2834	YML035C	No agreement	FALSE	1	0.115
2835	YNL118C	Agreement	TRUE	0	0.003
2836	YGL116W	No agreement	FALSE	0	0
2837	YOR367W	No agreement	FALSE	1	0.102

2838	YJL008C	No agreement	FALSE	0	0.091
2839	YER178W	Agreement	FALSE	1	0
2840	YPL048W	Agreement	FALSE	1	0.079
2841	YGR217W	No agreement	FALSE	1	0.132
2842	YLR088W	No agreement	TRUE	0	0.046
2843	YGR046W	Agreement	FALSE	0	0.033
2844	YLR300W	Agreement	FALSE	1	0.048
2845	YNL153C	Agreement	FALSE	1	0.225
2846	YDR326C	No agreement	FALSE	0	0.284
2847	YMR259C	Agreement	FALSE	1	0.268
2848	YPL015C	Agreement	FALSE	1	0.056
2849	YHR187W	No agreement	FALSE	0	0.229
2850	YNL243W	No agreement	TRUE	1	0.004
2851	YGL185C	No agreement	TRUE	0	0.046
2852	YLR410W	No agreement	FALSE	1	0.17
2853	YDL056W	No agreement	TRUE	1	0.133
2854	YBR103W	Agreement	TRUE	1	0.001
2855	YBR130C	No agreement	TRUE	1	0
2856	YBR240C	No agreement	FALSE	1	0
2857	YNL168C	Agreement	FALSE	1	0.253
2858	YGR196C	No agreement	TRUE	1	0.028
2859	YMR296C	Agreement	FALSE	0	0.027
2860	YPL146C	Agreement	TRUE	0	0.119
2861	YHR021C	Agreement	TRUE	1	0.009
2862	YGR158C	Agreement	TRUE	0	0.008
2863	YPL120W	No agreement	TRUE	1	0
2864	YBR166C	Agreement	FALSE	1	0.026
2865	YDR071C	Agreement	FALSE	1	0.219
2866	YMR269W	Agreement	FALSE	1	0.088
2867	YGL086W	No agreement	FALSE	1	0.23
2868	YGL151W	Agreement	FALSE	1	0.138
2869	YNL240C	No agreement	FALSE	0	0.038
2870	YBR078W	No agreement	FALSE	1	0
2871	YDL065C	Agreement	FALSE	1	0.217
2872	YHR099W	Agreement	FALSE	0	0
2873	YDR378C	No agreement	FALSE	1	0.035
2874	YOR201C	Agreement	TRUE	1	0
2875	YMR282C	No agreement	TRUE	1	0
2876	YOL011W	Agreement	FALSE	1	0.003
2877	YHL001W	Agreement	FALSE	0	0
2878	YML104C	No agreement	TRUE	1	0.054
2879	YGR123C	Agreement	FALSE	1	0.011
2880	YLR089C	No agreement	TRUE	1	0
2881	YML101C	No agreement	FALSE	1	0
2882	YFR033C	Agreement	TRUE	1	0
2883	YGR014W	No agreement	FALSE	1	0
2884	YIL106W	Agreement	FALSE	0	0.005
2885	YJL029C	Agreement	TRUE	1	0.005
2886	YPR043W	Agreement	FALSE	1	0

2887	YGR229C	No agreement	TRUE	1	0
2888	YFR021W	Agreement	FALSE	1	0.128
2889	YJL172W	Agreement	FALSE	1	0.208
2890	YMR281W	Agreement	FALSE	0	0.006
2891	YMR275C	No agreement	FALSE	1	0.194
2892	YML011C	No agreement	FALSE	1	0
2893	YKR066C	Agreement	FALSE	1	0
2894	YKL048C	Agreement	TRUE	1	0.135
2895	YJL167W	Agreement	FALSE	0	0.141
2896	YEL053C	No agreement	TRUE	1	0.001
2897	YIR010W	No agreement	TRUE	0	0
2898	YER183C	No agreement	FALSE	1	0.05
2899	YDL061C	Agreement	FALSE	1	0.005
2900	YNL231C	Agreement	TRUE	1	0
2901	YIL063C	No agreement	FALSE	0	0.162
2902	YDL028C	No agreement	TRUE	0	0.048
2903	YDL100C	Agreement	TRUE	1	0
2904	YLR260W	No agreement	FALSE	1	0.101
2905	YNL164C	No agreement	TRUE	1	0
2906	YPR191W	No agreement	TRUE	1	0.002
2907	YER072W	Agreement	TRUE	0	0
2908	YFL047W	Agreement	TRUE	1	0
2909	YMR252C	Agreement	FALSE	1	0.012
2910	YGL112C	No agreement	FALSE	0	0.045
2911	YLR066W	No agreement	FALSE	0	0.229
2912	YPL096W	No agreement	TRUE	1	0.002
2913	YLR333C	Agreement	FALSE	1	0
2914	YFR042W	No agreement	FALSE	0	0.011
2915	YJL012C	No agreement	FALSE	0	0.022
2916	YKL186C	Agreement	TRUE	0	0.115
2917	YKL198C	No agreement	FALSE	1	0.002
2918	YPL030W	Agreement	FALSE	1	0.041
2919	YHR108W	Agreement	FALSE	1	0.004
2920	YJL176C	Agreement	FALSE	1	0.278
2921	YKR031C	No agreement	TRUE	1	0.133
2922	YDL188C	Agreement	FALSE	1	0
2923	YLR065C	Agreement	FALSE	1	0.003
2924	YCR077C	Agreement	FALSE	1	0.036
2925	YNL236W	No agreement	FALSE	1	0.13
2926	YOL158C	Agreement	FALSE	1	0.162
2927	YDR275W	No agreement	FALSE	1	0.164
2928	YER089C	Agreement	FALSE	0	0.003
2929	YDR228C	Agreement	FALSE	0	0.273
2930	YGL099W	Agreement	TRUE	0	0.025
2931	YPR163C	Agreement	TRUE	1	0.011
2932	YNL268W	No agreement	FALSE	1	0.216
2933	YPL225W	Agreement	TRUE	1	0.005
2934	YMR071C	No agreement	TRUE	1	0.007
2935	YHR119W	Agreement	TRUE	0	0.024

2936	YGR251W	No agreement	TRUE	0	0.001
2937	YML019W	No agreement	FALSE	1	0.013
2938	YBR160W	Agreement	TRUE	0	0
2939	YNR057C	No agreement	FALSE	1	0.131
2940	YLR070C	Agreement	TRUE	1	0
2941	YMR032W	Agreement	FALSE	1	0
2942	YHL031C	No agreement	FALSE	1	0.002
2943	YNL008C	Agreement	FALSE	1	0
2944	YDL090C	No agreement	FALSE	1	0.036
2945	YKL109W	No agreement	TRUE	1	0
2946	YDL131W	Agreement	FALSE	1	0
2947	YHR013C	No agreement	TRUE	1	0.001
2948	YML015C	No agreement	TRUE	0	0.033
2949	YJR117W	Agreement	FALSE	1	0
2950	YDL025C	No agreement	TRUE	1	0.019
2951	YIR008C	Agreement	FALSE	0	0.002
2952	YBR245C	No agreement	TRUE	1	0.001
2953	YHR207C	No agreement	FALSE	1	0.234
2954	YOR120W	Agreement	TRUE	1	0
2955	YKL183W	No agreement	FALSE	1	0.237
2956	YDL095W	No agreement	FALSE	1	0
2957	YDR127W	No agreement	FALSE	1	0.188
2958	YBR129C	Agreement	FALSE	1	0.06
2959	YGR162W	Agreement	TRUE	0	0.002
2960	YER110C	Agreement	FALSE	1	0.015
2961	YIL014W	No agreement	FALSE	1	0.274
2962	YKL201C	Agreement	FALSE	1	0.04
2963	YJL078C	No agreement	FALSE	1	0
2964	YBR218C	No agreement	FALSE	1	0.032
2965	YLL048C	No agreement	FALSE	1	0.036
2966	YBR002C	No agreement	TRUE	0	0.053
2967	YER088C	Agreement	FALSE	1	0.008
2968	YGR131W	No agreement	TRUE	1	0.032
2969	YNR051C	No agreement	TRUE	1	0.011
2970	YJR104C	Agreement	FALSE	1	0.001
2971	YJL108C	Agreement	TRUE	1	0
2972	YNL224C	No agreement	TRUE	1	0.06
2973	YPR019W	No agreement	FALSE	0	0.013
2974	YHR077C	No agreement	TRUE	1	0.18
2975	YOL111C	Agreement	FALSE	1	0
2976	YPL154C	Agreement	FALSE	1	0
2977	YDR372C	No agreement	FALSE	1	0.124
2978	YOL125W	Agreement	TRUE	1	0.017
2979	YDR201W	Agreement	TRUE	0	0.01
2980	YDL236W	Agreement	FALSE	1	0
2981	YLR455W	No agreement	TRUE	1	0.066
2982	YMR307W	No agreement	TRUE	1	0
2983	YKL134C	No agreement	TRUE	1	0
2984	YOR221C	Agreement	FALSE	1	0.09

2985	YDR238C	No agreement	TRUE	0	0.056
2986	YGR200C	Agreement	FALSE	1	0.014
2987	YKL149C	Agreement	FALSE	1	0
2988	YBL084C	Agreement	TRUE	0	0.059
2989	YLL041C	Agreement	TRUE	1	0.002
2990	YHR001W-A	Agreement	FALSE	1	0.001
2991	YEL002C	No agreement	FALSE	0	0.074
2992	YHR100C	No agreement	FALSE	1	0.001
2993	YBR207W	Agreement	FALSE	1	0.016
2994	YIR012W	Agreement	FALSE	0	0.028
2995	YKL213C	Agreement	TRUE	1	0.001
2996	YNL133C	No agreement	TRUE	1	0
2997	YML072C	Agreement	FALSE	1	0
2998	YEL055C	Agreement	TRUE	0	0
2999	YAL019W	Agreement	TRUE	1	0.003
3000	YPL011C	Agreement	FALSE	0	0.234
3001	YJR121W	No agreement	FALSE	1	0
3002	YNL037C	Agreement	FALSE	1	0
3003	YML098W	No agreement	TRUE	0	0.002
3004	YGR271W	No agreement	FALSE	0	0.114
3005	YLR212C	Agreement	FALSE	0	0
3006	YGL142C	Agreement	FALSE	0	0.17
3007	YGL023C	No agreement	FALSE	1	0.245
3008	YKR003W	Agreement	TRUE	1	0
3009	YDL117W	No agreement	FALSE	1	0.003
3010	YEL007W	Agreement	FALSE	1	0.131
3011	YPL160W	Agreement	FALSE	0	0.001
3012	YNL158W	No agreement	FALSE	0	0.003
3013	YOL124C	Agreement	FALSE	1	0.089
3014	YDR139C	No agreement	FALSE	1	0.281
3015	YMR123W	Agreement	FALSE	1	0.034
3016	YKL061W	Agreement	FALSE	1	0.097
3017	YPR063C	Agreement	FALSE	1	0.272
3018	YCL005W	Agreement	FALSE	1	0.006
3019	YLR047C	No agreement	FALSE	1	0.124
3020	YPR036W	No agreement	FALSE	1	0.023
3021	YML069W	Agreement	TRUE	0	0.261
3022	YBR162C	No agreement	FALSE	1	0
3023	YCR038C	Agreement	TRUE	0	0.001
3024	YNL233W	Agreement	TRUE	1	0
3025	YKL181W	Agreement	FALSE	1	0.005
3026	YNR010W	No agreement	TRUE	1	0.008
3027	YBR061C	Agreement	FALSE	1	0.16
3028	YPR075C	Agreement	FALSE	1	0.061
3029	YOR129C	Agreement	FALSE	1	0.214
3030	YLR436C	Agreement	FALSE	1	0.002
3031	YJR112W	No agreement	TRUE	0	0.031
3032	YHR081W	No agreement	TRUE	1	0.163
3033	YOR226C	Agreement	FALSE	1	0.002

3034	YMR284W	Agreement	TRUE	1	0
3035	YAL022C	No agreement	FALSE	1	0.014
3036	YOL031C	Agreement	FALSE	1	0
3037	YIL009W	No agreement	FALSE	1	0.049
3038	YDR524C	No agreement	FALSE	1	0.172
3039	YOR390W	No agreement	FALSE	0	0.122
3040	YOR080W	Agreement	FALSE	1	0.004
3041	YPL094C	No agreement	FALSE	0	0.126
3042	YFL045C	Agreement	TRUE	0	0
3043	YML025C	No agreement	TRUE	0	0
3044	YGL140C	No agreement	FALSE	1	0.158
3045	YIL023C	No agreement	FALSE	1	0.256
3046	YHR079C	No agreement	FALSE	1	0.234
3047	YBR153W	No agreement	TRUE	0	0.078
3048	YER056C	Agreement	FALSE	1	0
3049	YOL053W	Agreement	TRUE	1	0
3050	YBR073W	Agreement	TRUE	1	0
3051	YOR332W	Agreement	TRUE	1	0.002
3052	YDR207C	No agreement	TRUE	1	0.018
3053	YML099C	Agreement	TRUE	1	0.004
3054	YAL040C	No agreement	FALSE	1	0.011
3055	YOR137C	Agreement	TRUE	1	0
3056	YIL022W	Agreement	TRUE	0	0
3057	YPL101W	No agreement	FALSE	1	0.053
3058	YML048W	Agreement	FALSE	1	0.005
3059	YLR328W	Agreement	FALSE	1	0.069
3060	YGL195W	Agreement	FALSE	1	0
3061	YHR032W	Agreement	FALSE	1	0.06
3062	YJL054W	No agreement	TRUE	0	0
3063	YGL213C	No agreement	FALSE	1	0.087
3064	YCR037C	No agreement	FALSE	1	0.058
3065	YGR165W	No agreement	TRUE	1	0
3066	YDR004W	No agreement	FALSE	1	0
3067	YPL227C	No agreement	FALSE	1	0.001
3068	YHR175W	Agreement	TRUE	0	0.018
3069	YLR082C	Agreement	FALSE	1	0.144
3070	YBR112C	Agreement	FALSE	1	0.055
3071	YBR020W	Agreement	FALSE	1	0.095
3072	YLL018C-A	Agreement	TRUE	1	0.027
3073	YFL018C	No agreement	FALSE	1	0.243
3074	YNL022C	No agreement	FALSE	1	0.262
3075	YLR191W	Agreement	TRUE	1	0.001
3076	YKL101W	No agreement	TRUE	1	0
3077	YOL005C	No agreement	FALSE	0	0.038
3078	YPR082C	Agreement	FALSE	0	0
3079	YDR303C	No agreement	FALSE	0	0.054
3080	YMR190C	No agreement	TRUE	1	0.004
3081	YKR038C	No agreement	FALSE	0	0
3082	YIL078W	Agreement	FALSE	0	0

3083	YJL177W	Agreement	TRUE	1	0
3084	YDR325W	No agreement	TRUE	0	0.012
3085	YLR352W	No agreement	FALSE	1	0
3086	YDL231C	No agreement	FALSE	1	0.098
3087	YLR051C	Agreement	TRUE	0	0.029
3088	YML026C	Agreement	FALSE	1	0
3089	YJL125C	Agreement	TRUE	0	0.063
3090	YMR080C	No agreement	FALSE	1	0.244
3091	YBR060C	Agreement	TRUE	0	0.002
3092	YDR422C	No agreement	FALSE	1	0.093
3093	YNL321W	Agreement	FALSE	1	0
3094	YHR151C	No agreement	TRUE	1	0.011
3095	YDL224C	Agreement	FALSE	1	0.262
3096	YGR099W	No agreement	FALSE	0	0.001
3097	YKL073W	No agreement	TRUE	1	0.015
3098	YBR146W	No agreement	FALSE	1	0
3099	YGR095C	Agreement	FALSE	0	0.088
3100	YOR126C	Agreement	FALSE	1	0.288
3101	YDL076C	Agreement	TRUE	1	0
3102	YBR080C	No agreement	TRUE	0	0.046
3103	YLR052W	No agreement	TRUE	1	0.135
3104	YGL155W	No agreement	FALSE	0	0.003
3105	YPL029W	No agreement	TRUE	1	0
3106	YNL071W	No agreement	FALSE	1	0
3107	YGR074W	Agreement	FALSE	0	0.174
3108	YBR135W	No agreement	FALSE	0	0.019
3109	YPR165W	No agreement	FALSE	0	0.232
3110	YBR093C	Agreement	FALSE	1	0.018
3111	YDL213C	Agreement	TRUE	1	0.024
3112	YNL125C	No agreement	FALSE	1	0
3113	YOR296W	Agreement	FALSE	1	0.098
3114	YML005W	No agreement	FALSE	1	0.253
3115	YLR081W	Agreement	FALSE	1	0.006
3116	YBR015C	No agreement	FALSE	1	0.038
3117	YBL056W	Agreement	FALSE	1	0.148
3118	YNR039C	No agreement	FALSE	1	0
3119	YFR034C	Agreement	TRUE	1	0.001
3120	YML110C	No agreement	FALSE	1	0
3121	YPR105C	Agreement	FALSE	0	0.225
3122	YHR162W	Agreement	FALSE	1	0.069
3123	YER177W	Agreement	TRUE	1	0.032
3124	YDR529C	Agreement	FALSE	1	0.001
3125	YNR029C	No agreement	TRUE	1	0.038
3126	YOR336W	No agreement	FALSE	0	0.005
3127	YIL125W	No agreement	FALSE	1	0
3128	YJR077C	No agreement	FALSE	1	0
3129	YPL211W	Agreement	FALSE	0	0.021
3130	YDR407C	Agreement	FALSE	0	0.166
3131	YER090W	No agreement	FALSE	1	0

3132	YLR077W	No agreement	TRUE	1	0
3133	YMR083W	Agreement	FALSE	1	0
3134	YMR233W	No agreement	TRUE	1	0.117
3135	YLR072W	Agreement	TRUE	1	0.013
3136	YBR034C	Agreement	FALSE	1	0.073
3137	YOR304W	No agreement	TRUE	1	0.079
3138	YPR041W	Agreement	FALSE	0	0.003
3139	YEL006W	No agreement	FALSE	1	0.039
3140	YJL156C	No agreement	TRUE	0	0.113
3141	YDR043C	No agreement	TRUE	1	0.022
3142	YKR008W	Agreement	TRUE	0	0.033
3143	YCR028C-A	Agreement	FALSE	1	0
3144	YER157W	No agreement	TRUE	0	0
3145	YML024W	Agreement	TRUE	1	0.01
3146	YBR055C	No agreement	TRUE	0	0.045
3147	YBR030W	Agreement	TRUE	1	0.05
3148	YIL024C	No agreement	FALSE	1	0.279
3149	YPL010W	No agreement	FALSE	0	0.078
3150	YLR078C	No agreement	FALSE	0	0.234
3151	YNL099C	Agreement	TRUE	1	0.049
3152	YGR206W	Agreement	FALSE	1	0.006
3153	YBL003C	Agreement	FALSE	1	0
3154	YNL087W	No agreement	FALSE	1	0.001
3155	YMR074C	Agreement	FALSE	1	0.113
3156	YJL206C	Agreement	FALSE	1	0.09
3157	YDR379W	Agreement	TRUE	1	0.022
3158	YDL067C	No agreement	FALSE	1	0
3159	YDL111C	No agreement	FALSE	0	0.178
3160	YBL024W	Agreement	TRUE	1	0.043
3161	YNL172W	Agreement	FALSE	0	0.178
3162	YBL101C	No agreement	FALSE	1	0
3163	YLR026C	No agreement	TRUE	0	0
3164	YLR189C	Agreement	TRUE	1	0
3165	YFL031W	No agreement	FALSE	1	0
3166	YMR209C	Agreement	FALSE	1	0.01
3167	YMR111C	No agreement	FALSE	1	0.142
3168	YDR348C	No agreement	TRUE	1	0
3169	YLR106C	No agreement	TRUE	0	0.016
3170	YOR212W	No agreement	FALSE	1	0.049
3171	YPL049C	No agreement	TRUE	1	0.007
3172	YNL290W	Agreement	TRUE	0	0
3173	YMR200W	No agreement	FALSE	0	0
3174	YNL238W	No agreement	FALSE	1	0.121
3175	YPR164W	No agreement	FALSE	1	0.001
3176	YMR043W	No agreement	TRUE	0	0.012
3177	YER123W	Agreement	FALSE	1	0.239
3178	YDR293C	Agreement	FALSE	1	0
3179	YMR304W	Agreement	TRUE	1	0
3180	YJL139C	No agreement	FALSE	1	0

3181	YJL183W	Agreement	FALSE	1	0
3182	YIL090W	No agreement	TRUE	1	0.002
3183	YIL137C	No agreement	FALSE	1	0.013
3184	YIL076W	No agreement	TRUE	1	0.012
3185	YNL038W	No agreement	FALSE	0	0.263
3186	YHR115C	Agreement	FALSE	1	0.238
3187	YLR443W	No agreement	FALSE	1	0.066
3188	YNL027W	Agreement	TRUE	1	0.001
3189	YCR024C-A	No agreement	FALSE	1	0.002
3190	YLL031C	Agreement	FALSE	0	0.157
3191	YBR285W	Agreement	FALSE	1	0
3192	YOR094W	No agreement	FALSE	1	0.061
3193	YHR030C	No agreement	FALSE	1	0.165
3194	YDR120C	Agreement	TRUE	1	0.019
3195	YBL023C	Agreement	TRUE	0	0.044
3196	YKL080W	Agreement	FALSE	1	0.18
3197	YPL221W	No agreement	FALSE	1	0.068
3198	YFR051C	No agreement	FALSE	0	0
3199	YDR197W	Agreement	TRUE	1	0
3200	YLR376C	Agreement	FALSE	1	0.187
3201	YGR119C	No agreement	FALSE	0	0.249
3202	YLR262C-A	Agreement	FALSE	1	0.109
3203	YHL017W	Agreement	FALSE	1	0.015
3204	YJL207C	No agreement	FALSE	1	0.219
3205	YOR234C	Agreement	FALSE	1	0
3206	YGR237C	Agreement	TRUE	1	0
3207	YDR339C	Agreement	TRUE	0	0.016
3208	YNR052C	No agreement	TRUE	1	0.02
3209	YPR025C	Agreement	TRUE	0	0.049
3210	YGL128C	No agreement	FALSE	0	0.186
3211	YLR181C	Agreement	TRUE	1	0.025
3212	YCR076C	Agreement	TRUE	1	0
3213	YKL085W	No agreement	FALSE	1	0
3214	YBR143C	Agreement	FALSE	0	0.013
3215	YER078C	Agreement	TRUE	1	0
3216	YMR121C	No agreement	TRUE	1	0
3217	YER092W	Agreement	FALSE	1	0.164
3218	YML077W	No agreement	TRUE	0	0.002
3219	YOR384W	Agreement	FALSE	1	0.012
3220	YJL020C	Agreement	FALSE	1	0
3221	YOR046C	Agreement	TRUE	0	0.01
3222	YER100W	Agreement	TRUE	0	0
3223	YNL271C	Agreement	TRUE	1	0
3224	YIL129C	No agreement	FALSE	0	0.042
3225	YBR227C	No agreement	TRUE	1	0.044
3226	YNL166C	No agreement	TRUE	1	0
3227	YPL169C	Agreement	FALSE	0	0.108
3228	YDL044C	No agreement	TRUE	1	0
3229	YDR016C	Agreement	TRUE	0	0

3230	YGL018C	No agreement	TRUE	0	0
3231	YGR093W	Agreement	TRUE	1	0.009
3232	YER086W	No agreement	FALSE	1	0.005
3233	YGL083W	No agreement	FALSE	1	0.061
3234	YJL095W	No agreement	TRUE	1	0.108
3235	YEL061C	No agreement	TRUE	1	0.001
3236	YER118C	No agreement	FALSE	1	0
3237	YMR283C	No agreement	TRUE	1	0.034
3238	YAL044C	Agreement	TRUE	1	0.005
3239	YMR255W	Agreement	FALSE	1	0.255
3240	YGL066W	No agreement	FALSE	1	0.269
3241	YBR041W	Agreement	FALSE	1	0
3242	YER013W	No agreement	FALSE	0	0.162
3243	YMR313C	Agreement	FALSE	1	0
3244	YBR238C	No agreement	FALSE	1	0.203
3245	YER076C	No agreement	FALSE	0	0.102
3246	YGR079W	No agreement	TRUE	1	0.131
3247	YDR131C	Agreement	FALSE	1	0.052
3248	YER070W	Agreement	FALSE	1	0
3249	YDL042C	No agreement	TRUE	1	0.113
3250	YGR288W	Agreement	FALSE	1	0.021
3251	YLR244C	Agreement	FALSE	1	0.011
3252	YOL149W	No agreement	TRUE	0	0.001
3253	YOL030W	No agreement	TRUE	1	0
3254	YIL103W	Agreement	FALSE	1	0.027
3255	YDR143C	No agreement	FALSE	1	0.02
3256	YLR242C	No agreement	FALSE	1	0.272
3257	YDR506C	No agreement	TRUE	1	0.007
3258	YLR014C	Agreement	FALSE	1	0.031
3259	YIL001W	No agreement	FALSE	1	0.239
3260	YBR066C	Agreement	TRUE	1	0
3261	YML049C	Agreement	TRUE	0	0
3262	YLR015W	No agreement	FALSE	1	0.12
3263	YKR090W	No agreement	TRUE	1	0
3264	YNL259C	No agreement	TRUE	1	0
3265	YDL176W	No agreement	FALSE	1	0.087
3266	YBR017C	Agreement	FALSE	1	0.04
3267	YJR076C	No agreement	TRUE	0	0
3268	YDR471W	Agreement	FALSE	1	0.001
3269	YGR189C	No agreement	FALSE	1	0.03
3270	YHL011C	Agreement	FALSE	1	0.004
3271	YBR297W	No agreement	FALSE	1	0
3272	YER082C	Agreement	TRUE	0	0.051
3273	YLR214W	No agreement	FALSE	1	0.023
3274	YIR035C	No agreement	FALSE	1	0.148
3275	YCR035C	No agreement	TRUE	0	0.081
3276	YNL256W	Agreement	FALSE	0	0.124
3277	YML059C	Agreement	FALSE	1	0.006
3278	YDL190C	Agreement	FALSE	1	0.123

3279	YLR335W	No agreement	TRUE	1	0
3280	YDR126W	No agreement	FALSE	1	0.054
3281	YMR251W	No agreement	FALSE	1	0
3282	YHR098C	No agreement	TRUE	0	0
3283	YMR140W	Agreement	FALSE	1	0
3284	YBR198C	Agreement	TRUE	0	0.032
3285	YPL244C	No agreement	FALSE	1	0.001
3286	YGL203C	No agreement	TRUE	1	0.001
3287	YBR169C	Agreement	TRUE	1	0
3288	YOL051W	No agreement	TRUE	1	0.003
3289	YOR066W	Agreement	TRUE	1	0.002
3290	YDR151C	No agreement	FALSE	1	0
3291	YGR195W	No agreement	FALSE	0	0.01
3292	YKR096W	Agreement	TRUE	1	0.069
3293	YBL036C	Agreement	TRUE	1	0.002
3294	YGL064C	No agreement	TRUE	1	0
3295	YDL052C	Agreement	FALSE	1	0
3296	YDR034C	No agreement	TRUE	1	0.037
3297	YOL004W	Agreement	FALSE	1	0.117
3298	YOR096W	Agreement	FALSE	1	0
3299	YKL057C	Agreement	FALSE	1	0
3300	YBR154C	Agreement	TRUE	0	0.012
3301	YKL130C	Agreement	FALSE	1	0.28
3302	YBR228W	No agreement	FALSE	1	0.004
3303	YGL206C	Agreement	TRUE	1	0.003
3304	YGR083C	No agreement	TRUE	0	0.021
3305	YLR224W	No agreement	FALSE	1	0.047
3306	YNL094W	No agreement	FALSE	1	0.067
3307	YPL158C	No agreement	TRUE	0	0
3308	YER056C-A	Agreement	FALSE	1	0
3309	YJR089W	Agreement	FALSE	0	0.286
3310	YKL219W	No agreement	FALSE	0	0.114
3311	YPR069C	No agreement	FALSE	1	0.237
3312	YIL133C	Agreement	FALSE	1	0.002
3313	YNL138W	Agreement	TRUE	1	0.006
3314	YOR127W	Agreement	TRUE	1	0.001
3315	YBR074W	Agreement	FALSE	1	0
3316	YDR009W	No agreement	FALSE	1	0.018
3317	YER009W	Agreement	FALSE	0	0.004
3318	YPR173C	No agreement	FALSE	1	0.105
3319	YOR194C	No agreement	FALSE	0	0.104
3320	YEL009C	Agreement	TRUE	1	0.029
3321	YER125W	Agreement	FALSE	0	0.045
3322	YGL225W	Agreement	FALSE	0	0
3323	YDR128W	Agreement	TRUE	1	0
3324	YOR112W	No agreement	FALSE	1	0.001
3325	YFR006W	Agreement	FALSE	1	0
3326	YNL255C	Agreement	TRUE	1	0.001
3327	YIL053W	No agreement	FALSE	1	0

3328	YER105C	No agreement	FALSE	0	0.141
3329	YAL024C	Agreement	TRUE	0	0.001
3330	YDR177W	No agreement	TRUE	0	0.266
3331	YNL245C	No agreement	FALSE	0	0.013
3332	YGL187C	No agreement	FALSE	0	0
3333	YPL057C	No agreement	FALSE	1	0
3334	YMR323W	Agreement	FALSE	0	0
3335	YLR223C	Agreement	TRUE	0	0.133
3336	YBR200W	Agreement	FALSE	1	0.122
3337	YOL094C	Agreement	TRUE	0	0
3338	YBL068W	No agreement	FALSE	1	0
3339	YDR483W	No agreement	FALSE	1	0.041
3340	YLR152C	No agreement	FALSE	1	0.005
3341	YGR202C	Agreement	TRUE	1	0.039
3342	YNL004W	Agreement	TRUE	1	0.069
3343	YGR222W	Agreement	TRUE	1	0.088
3344	YDR377W	Agreement	FALSE	1	0
3345	YPL157W	No agreement	FALSE	1	0.155
3346	YIR007W	Agreement	FALSE	1	0.058
3347	YDR321W	Agreement	FALSE	1	0.031
3348	YOL039W	Agreement	FALSE	1	0
3349	YCR065W	Agreement	FALSE	1	0
3350	YPR070W	Agreement	FALSE	1	0.109
3351	YIL020C	No agreement	FALSE	1	0.027
3352	YMR256C	Agreement	FALSE	1	0
3353	YJR056C	Agreement	TRUE	1	0.152
3354	YGR170W	No agreement	TRUE	1	0.023
3355	YBR188C	No agreement	TRUE	1	0.003
3356	YBL009W	Agreement	FALSE	1	0
3357	YKL146W	Agreement	FALSE	1	0.145
3358	YOR176W	Agreement	FALSE	0	0.001
3359	YBR039W	No agreement	FALSE	1	0
3360	YGR020C	Agreement	FALSE	1	0
3361	YER185W	Agreement	FALSE	1	0.166
3362	YLR071C	Agreement	FALSE	0	0.077
3363	YOR281C	No agreement	FALSE	0	0.111
3364	YPL001W	No agreement	FALSE	1	0.266
3365	YDR353W	No agreement	FALSE	0	0
3366	YGL200C	No agreement	FALSE	1	0
3367	YFR001W	No agreement	TRUE	1	0.17
3368	YHR123W	Agreement	FALSE	1	0
3369	YGL107C	No agreement	TRUE	1	0
3370	YCL050C	No agreement	FALSE	1	0
3371	YFR029W	No agreement	FALSE	0	0.174
3372	YDR227W	Agreement	TRUE	1	0.03
3373	YLR043C	No agreement	FALSE	1	0.057
3374	YLR086W	No agreement	FALSE	0	0.203
3375	YFR032C-A	Agreement	FALSE	1	0
3376	YDR291W	No agreement	TRUE	1	0.001

3377	YGL247W	No agreement	FALSE	0	0.024
3378	YKL006C-A	Agreement	TRUE	0	0.002
3379	YNL091W	Agreement	TRUE	1	0
3380	YDL112W	Agreement	FALSE	1	0.029
3381	YEL032W	Agreement	FALSE	0	0
3382	YDR364C	No agreement	FALSE	1	0.038
3383	YAL043C	Agreement	FALSE	0	0.21
3384	YDL219W	No agreement	FALSE	1	0
3385	YGR075C	No agreement	TRUE	0	0.27
3386	YML085C	No agreement	FALSE	0	0
3387	YER007C-A	Agreement	TRUE	1	0.003
3388	YDR459C	No agreement	FALSE	1	0.044
3389	YFL022C	Agreement	FALSE	0	0
3390	YJR091C	Agreement	FALSE	1	0.001
3391	YDR489W	Agreement	FALSE	0	0.001
3392	YPR139C	No agreement	FALSE	1	0.137
3393	YDR211W	Agreement	TRUE	0	0.01
3394	YPL143W	Agreement	FALSE	0	0
3395	YEL018W	No agreement	TRUE	1	0.132
3396	YMR020W	No agreement	FALSE	1	0.215
3397	YKL025C	No agreement	FALSE	1	0.283
3398	YDR030C	Agreement	TRUE	1	0
3399	YCR067C	No agreement	TRUE	1	0
3400	YDL212W	No agreement	FALSE	0	0.185
3401	YOR118W	Agreement	TRUE	1	0
3402	YNL314W	No agreement	FALSE	1	0.05
3403	YDL125C	Agreement	FALSE	1	0
3404	YDL116W	No agreement	FALSE	1	0.113
3405	YOR151C	No agreement	TRUE	0	0.057
3406	YPL128C	No agreement	FALSE	0	0.03
3407	YLR208W	Agreement	FALSE	0	0.003
3408	YOR378W	No agreement	FALSE	1	0.069
3409	YNL246W	Agreement	FALSE	1	0.057
3410	YDL005C	Agreement	TRUE	1	0.004
3411	YNL199C	No agreement	TRUE	1	0.107
3412	YHL026C	No agreement	FALSE	1	0.047
3413	YGR060W	No agreement	FALSE	0	0
3414	YNL267W	No agreement	TRUE	0	0.031
3415	YMR218C	No agreement	FALSE	0	0.041
3416	YER098W	No agreement	TRUE	1	0.036
3417	YBR171W	Agreement	TRUE	1	0.122
3418	YBL103C	No agreement	TRUE	1	0.001
3419	YOL103W	No agreement	FALSE	1	0.01
3420	YLR064W	Agreement	FALSE	1	0.011
3421	YNL010W	No agreement	FALSE	1	0
3422	YMR164C	Agreement	TRUE	1	0
3423	YDR335W	Agreement	FALSE	1	0.272
3424	YBR048W	Agreement	FALSE	1	0.001
3425	YGR257C	Agreement	FALSE	0	0

3426	YIL118W	No agreement	FALSE	0	0.178
3427	YER011W	No agreement	TRUE	1	0.002
3428	YNL006W	No agreement	FALSE	0	0.247
3429	YOR369C	Agreement	FALSE	1	0.001
3430	YIL064W	Agreement	TRUE	1	0.003
3431	YDR361C	Agreement	TRUE	0	0.02
3432	YKL081W	Agreement	FALSE	1	0
3433	YBR071W	No agreement	FALSE	1	0.001
3434	YDR210W	No agreement	TRUE	1	0
3435	YKL051W	No agreement	FALSE	1	0.001
3436	YLR096W	No agreement	TRUE	1	0.058
3437	YNL126W	No agreement	TRUE	0	0
3438	YNR028W	Agreement	FALSE	1	0.001
3439	YBR072W	No agreement	FALSE	1	0
3440	YNL257C	Agreement	TRUE	1	0.004
3441	YKL047W	Agreement	TRUE	1	0.024
3442	YAL063C	Agreement	FALSE	0	0.077
3443	YLR276C	Agreement	TRUE	0	0.064
3444	YOR101W	No agreement	TRUE	1	0.024
3445	YLR383W	Agreement	TRUE	0	0
3446	YGL139W	No agreement	FALSE	1	0.017
3447	YKL139W	No agreement	TRUE	1	0.166
3448	YOL049W	Agreement	TRUE	1	0
3449	YDL030W	Agreement	FALSE	0	0.274
3450	YJL079C	No agreement	FALSE	1	0.048
3451	YJR105W	Agreement	FALSE	1	0.002
3452	YML105C	Agreement	TRUE	0	0.03
3453	YKL046C	No agreement	FALSE	1	0
3454	YDL157C	Agreement	FALSE	1	0
3455	YOR038C	No agreement	FALSE	1	0
3456	YHR076W	No agreement	TRUE	1	0
3457	YOL061W	Agreement	FALSE	1	0.007
3458	YPL272C	Agreement	FALSE	1	0.204
3459	YER127W	Agreement	TRUE	0	0.057
3460	YKL032C	Agreement	TRUE	1	0
3461	YLR187W	Agreement	FALSE	1	0.006
3462	YOR020C	No agreement	FALSE	0	0
3463	YDR399W	Agreement	FALSE	1	0.009
3464	YGR210C	No agreement	TRUE	1	0
3465	YIL085C	No agreement	FALSE	0	0.256
3466	YLR144C	No agreement	FALSE	1	0
3467	YDL046W	Agreement	FALSE	1	0
3468	YIL147C	No agreement	FALSE	0	0.035
3469	YBL060W	Agreement	TRUE	1	0.036
3470	YKL033W	No agreement	TRUE	0	0.02
3471	YNL032W	No agreement	TRUE	1	0.001
3472	YOR206W	Agreement	TRUE	0	0.079
3473	YKR071C	Agreement	FALSE	0	0.151
3474	YDR530C	Agreement	TRUE	1	0

3475	YOR131C	No agreement	TRUE	1	0
3476	YPR046W	No agreement	TRUE	1	0.011
3477	YDL155W	Agreement	FALSE	1	0.269
3478	YBR086C	Agreement	FALSE	1	0.258
3479	YBL045C	Agreement	TRUE	1	0
3480	YLR398C	Agreement	TRUE	1	0.001
3481	YOR079C	No agreement	FALSE	1	0
3482	YLL043W	No agreement	FALSE	1	0.103
3483	YGL016W	Agreement	FALSE	1	0.087
3484	YNL047C	No agreement	FALSE	0	0.001
3485	YGR094W	Agreement	FALSE	0	0
3486	YDL128W	No agreement	FALSE	1	0.026
3487	YIR029W	No agreement	FALSE	1	0.003
3488	YOR304C-A	Agreement	TRUE	1	0.051
3489	YBR276C	Agreement	FALSE	1	0.153
3490	YJR073C	Agreement	FALSE	1	0
3491	YOL142W	No agreement	FALSE	0	0.288
3492	YLR215C	Agreement	TRUE	0	0.002
3493	YNL193W	Agreement	TRUE	1	0.266
3494	YBL018C	Agreement	TRUE	0	0.138
3495	YPL112C	No agreement	FALSE	1	0.088
3496	YJR054W	No agreement	TRUE	1	0.062
3497	YBR049C	Agreement	FALSE	0	0.148
3498	YDR054C	Agreement	TRUE	0	0.004
3499	YDR463W	No agreement	FALSE	1	0.02
3500	YJR094W-A	Agreement	FALSE	0	0
3501	YPL176C	No agreement	FALSE	1	0.207
3502	YHR172W	Agreement	FALSE	0	0
3503	YHR003C	No agreement	TRUE	1	0.007
3504	YHR073W	No agreement	FALSE	1	0.069
3505	YDR351W	No agreement	TRUE	1	0.05
3506	YLR104W	No agreement	FALSE	1	0.024
3507	YJL014W	No agreement	FALSE	0	0.233
3508	YLR126C	No agreement	TRUE	1	0.029
3509	YNL088W	No agreement	TRUE	0	0
3510	YJL187C	Agreement	TRUE	1	0
3511	YPR132W	Agreement	FALSE	1	0.254
3512	YOR090C	No agreement	TRUE	1	0.04
3513	YDL145C	No agreement	FALSE	0	0
3514	YOR253W	Agreement	FALSE	1	0.189
3515	YLL003W	Agreement	FALSE	0	0.074
3516	YGL126W	No agreement	FALSE	1	0.028
3517	YGR282C	No agreement	FALSE	1	0.215
3518	YGL131C	Agreement	FALSE	1	0.021
3519	YGL238W	No agreement	FALSE	0	0.141
3520	YPL194W	Agreement	FALSE	1	0.086
3521	YDR146C	Agreement	TRUE	1	0
3522	YLR367W	Agreement	TRUE	1	0
3523	YLR437C	Agreement	FALSE	1	0

3524	YBL055C	No agreement	FALSE	1	0.241
3525	YKL127W	Agreement	FALSE	1	0.002
3526	YER107C	Agreement	FALSE	1	0.001
3527	YBR127C	Agreement	FALSE	1	0.121
3528	YDR329C	Agreement	TRUE	1	0
3529	YDL075W	Agreement	FALSE	1	0.001
3530	YHR210C	No agreement	FALSE	1	0.004
3531	YHR135C	Agreement	TRUE	1	0.002
3532	YKR026C	Agreement	FALSE	1	0.008
3533	YPL193W	Agreement	TRUE	1	0.001
3534	YHR084W	Agreement	TRUE	1	0.013
3535	YNL067W	Agreement	TRUE	1	0
3536	YDR307W	Agreement	FALSE	1	0.11
3537	YMR287C	No agreement	TRUE	1	0
3538	YNL323W	No agreement	TRUE	0	0
3539	YGR214W	Agreement	TRUE	1	0
3540	YJL030W	No agreement	FALSE	1	0.146
3541	YPR129W	Agreement	TRUE	1	0.038
3542	YML020W	No agreement	FALSE	1	0.057
3543	YOR122C	Agreement	FALSE	0	0
3544	YML115C	No agreement	FALSE	1	0.003
3545	YNL044W	Agreement	FALSE	1	0
3546	YGL211W	No agreement	TRUE	1	0.129
3547	YDR109C	No agreement	TRUE	1	0.004
3548	YPR183W	Agreement	FALSE	0	0
3549	YIL109C	Agreement	FALSE	0	0.075
3550	YJR044C	Agreement	FALSE	0	0
3551	YNL021W	No agreement	TRUE	1	0
3552	YIR033W	No agreement	FALSE	1	0.115
3553	YAL029C	No agreement	TRUE	1	0.082
3554	YOR179C	No agreement	FALSE	1	0.144
3555	YDR301W	No agreement	FALSE	0	0.17
3556	YJL147C	Agreement	FALSE	1	0.275
3557	YPR024W	Agreement	TRUE	1	0
3558	YPR117W	Agreement	TRUE	1	0
3559	YJR010C-A	Agreement	FALSE	1	0
3560	YGL073W	No agreement	TRUE	0	0
3561	YBR082C	Agreement	FALSE	1	0.003
3562	YOR256C	Agreement	TRUE	0	0.042
3563	YJR093C	No agreement	TRUE	0	0.17
3564	YIL016W	Agreement	FALSE	1	0.183
3565	YER096W	No agreement	FALSE	1	0
3566	YFR009W	Agreement	FALSE	1	0.004
3567	YMR309C	Agreement	FALSE	0	0.009
3568	YOR269W	Agreement	FALSE	1	0.048
3569	YDR266C	Agreement	TRUE	1	0.019
3570	YOR217W	Agreement	TRUE	0	0.021
3571	YGL085W	Agreement	FALSE	1	0.238
3572	YDR183W	No agreement	TRUE	1	0

3573	YIL138C	Agreement	TRUE	1	0.03
3574	YJL002C	No agreement	FALSE	0	0.189
3575	YGL070C	No agreement	FALSE	1	0.008
3576	YKR004C	Agreement	FALSE	0	0.012
3577	YLL032C	Agreement	TRUE	1	0
3578	YBR133C	No agreement	FALSE	1	0
3579	YML094W	No agreement	FALSE	1	0.002
3580	YPR143W	Agreement	TRUE	0	0.034
3581	YDR041W	No agreement	FALSE	0	0
3582	YCR051W	No agreement	TRUE	1	0.009
3583	YJL061W	No agreement	TRUE	0	0.242
3584	YKL117W	Agreement	TRUE	1	0.001
3585	YKL148C	Agreement	TRUE	1	0
3586	YER114C	No agreement	TRUE	1	0.269
3587	YHL035C	No agreement	FALSE	1	0.097
3588	YFR039C	No agreement	FALSE	1	0.172
3589	YHR163W	No agreement	FALSE	1	0.027
3590	YJL084C	Agreement	FALSE	1	0
3591	YLR154C	Agreement	FALSE	1	0.104
3592	YIL110W	Agreement	FALSE	1	0.021
3593	YOR347C	Agreement	FALSE	1	0.011
3594	YPR156C	Agreement	FALSE	1	0.21
3595	YPL039W	No agreement	TRUE	1	0.007
3596	YFL039C	No agreement	TRUE	0	0.024
3597	YPL246C	No agreement	FALSE	1	0.121
3598	YPL131W	Agreement	FALSE	0	0
3599	YML081C-A	No agreement	FALSE	1	0
3600	YGR098C	No agreement	FALSE	0	0
3601	YKL144C	No agreement	FALSE	0	0.039
3602	YAL051W	Agreement	FALSE	1	0.223
3603	YGL039W	Agreement	FALSE	1	0
3604	YMR277W	Agreement	TRUE	0	0.001
3605	YPR062W	No agreement	FALSE	1	0.14
3606	YDR520C	No agreement	FALSE	1	0.02
3607	YMR047C	Agreement	TRUE	0	0.029
3608	YOR370C	No agreement	TRUE	0	0.008
3609	YFL038C	Agreement	TRUE	0	0.052
3610	YGR227W	No agreement	FALSE	1	0.198
3611	YKR064W	No agreement	FALSE	1	0.193
3612	YKL092C	No agreement	TRUE	1	0.006
3613	YPR033C	Agreement	FALSE	0	0.001
3614	YDL001W	No agreement	TRUE	1	0.237
3615	YGR085C	Agreement	FALSE	1	0.001
3616	YOR058C	Agreement	FALSE	1	0
3617	YLL018C	No agreement	FALSE	0	0
3618	YGR286C	Agreement	TRUE	1	0.084
3619	YOR270C	No agreement	TRUE	1	0.005
3620	YER017C	Agreement	FALSE	1	0
3621	YPL003W	No agreement	FALSE	1	0

3622	YDR152W	No agreement	TRUE	1	0.001
3623	YNL023C	No agreement	TRUE	1	0.002
3624	YPL081W	No agreement	FALSE	1	0
3625	YDR490C	No agreement	TRUE	1	0.002
3626	YBR170C	No agreement	TRUE	1	0.039
3627	YOR249C	No agreement	FALSE	0	0.172
3628	YLR382C	No agreement	TRUE	1	0
3629	YLR038C	Agreement	FALSE	1	0
3630	YOR113W	Agreement	TRUE	1	0
3631	YPR114W	Agreement	TRUE	1	0
3632	YNL085W	No agreement	FALSE	1	0.179
3633	YHR058C	No agreement	TRUE	0	0.009
3634	YLR438C-A	No agreement	TRUE	0	0.005
3635	YLR229C	No agreement	FALSE	0	0.004
3636	YMR033W	Agreement	TRUE	0	0
3637	YBR018C	Agreement	FALSE	1	0
3638	YLR259C	No agreement	TRUE	0	0.004
3639	YNL154C	Agreement	TRUE	1	0.004
3640	YDR093W	Agreement	FALSE	1	0
3641	YOR017W	Agreement	TRUE	1	0
3642	YJL121C	Agreement	FALSE	1	0.285
3643	YOR334W	Agreement	FALSE	1	0.001
3644	YLR440C	Agreement	TRUE	0	0.09
3645	YIL123W	No agreement	FALSE	1	0
3646	YMR037C	Agreement	FALSE	1	0.007
3647	YAL013W	No agreement	TRUE	1	0.007
3648	YOL073C	Agreement	FALSE	0	0
3649	YNL301C	Agreement	FALSE	1	0.002
3650	YNL291C	Agreement	FALSE	1	0
3651	YPL199C	No agreement	TRUE	1	0
3652	YHR117W	No agreement	FALSE	1	0.011
3653	YGL189C	Agreement	FALSE	0	0.181
3654	YDL004W	No agreement	FALSE	0	0
3655	YDR186C	No agreement	TRUE	1	0.152
3656	YKL074C	No agreement	TRUE	1	0.081
3657	YBR047W	No agreement	FALSE	1	0
3658	YKL180W	Agreement	TRUE	0	0
3659	YLL022C	No agreement	TRUE	0	0.056
3660	YAL048C	Agreement	FALSE	1	0.066
3661	YBR252W	Agreement	TRUE	0	0
3662	YPL172C	Agreement	FALSE	1	0
3663	YPL047W	No agreement	FALSE	1	0.007
3664	YPR188C	No agreement	FALSE	1	0.282
3665	YNL287W	No agreement	TRUE	0	0.002
3666	YGL256W	No agreement	FALSE	1	0.258
3667	YDR403W	Agreement	FALSE	1	0.008
3668	YDR518W	No agreement	FALSE	1	0.022
3669	YCR043C	No agreement	FALSE	1	0.002
3670	YMR214W	No agreement	TRUE	1	0.002

3671	YKR094C	Agreement	FALSE	1	0
3672	YPR111W	Agreement	TRUE	1	0.004
3673	YJL104W	No agreement	TRUE	0	0
3674	YGL058W	No agreement	TRUE	1	0.221
3675	YPL271W	No agreement	FALSE	1	0.002
3676	YEL059C-A	Agreement	FALSE	0	0
3677	YLR395C	Agreement	FALSE	1	0
3678	YOR178C	Agreement	FALSE	1	0
3679	YNL039W	No agreement	TRUE	0	0.205
3680	YIL039W	No agreement	FALSE	1	0.001
3681	YPL037C	Agreement	FALSE	1	0.186
3682	YER004W	Agreement	TRUE	1	0
3683	YMR185W	No agreement	TRUE	0	0.257
3684	YOR246C	No agreement	FALSE	1	0
3685	YLR245C	No agreement	FALSE	0	0.242
3686	YJR042W	No agreement	FALSE	0	0.229
3687	YGR166W	No agreement	TRUE	1	0
3688	YKR037C	Agreement	TRUE	0	0
3689	YML070W	Agreement	TRUE	1	0
3690	YLR110C	No agreement	TRUE	1	0.095
3691	YGL014W	No agreement	FALSE	1	0.26
3692	YLR167W	Agreement	FALSE	0	0.08
3693	YHR063C	Agreement	FALSE	0	0
3694	YPL270W	Agreement	FALSE	1	0.274
3695	YBR167C	Agreement	TRUE	0	0
3696	YDR497C	Agreement	FALSE	1	0
3697	YGR054W	No agreement	FALSE	1	0
3698	YCR053W	No agreement	FALSE	1	0.015
3699	YBL082C	No agreement	FALSE	1	0.001
3700	YJR003C	Agreement	FALSE	1	0.259
3701	YDL232W	No agreement	FALSE	1	0
3702	YPL125W	No agreement	TRUE	1	0.094
3703	YGL172W	No agreement	FALSE	0	0.106
3704	YDR017C	No agreement	TRUE	1	0.1
3705	YER137C	Agreement	TRUE	1	0.066
3706	YNL189W	Agreement	FALSE	0	0.001
3707	YNL197C	Agreement	FALSE	1	0
3708	YKR082W	Agreement	TRUE	1	0.156
3709	YDR392W	No agreement	FALSE	1	0.059
3710	YOL089C	Agreement	FALSE	1	0
3711	YHR023W	Agreement	TRUE	0	0
3712	YLR185W	Agreement	FALSE	1	0
3713	YAR015W	Agreement	TRUE	1	0.001
3714	YML119W	Agreement	FALSE	1	0
3715	YEL031W	No agreement	FALSE	1	0
3716	YBR123C	No agreement	TRUE	0	0
3717	YLR272C	No agreement	TRUE	0	0
3718	YCR081W	No agreement	FALSE	1	0.229
3719	YPL151C	No agreement	TRUE	0	0.108

3720	YHL029C	No agreement	FALSE	1	0.027
3721	YDR454C	Agreement	TRUE	0	0.007
3722	YMR273C	No agreement	TRUE	1	0.092
3723	YKL022C	No agreement	FALSE	0	0.187
3724	YIL035C	No agreement	FALSE	1	0.291
3725	YGR092W	Agreement	TRUE	1	0
3726	YKL214C	Agreement	FALSE	1	0.079
3727	YFL017W-A	No agreement	FALSE	0	0.257
3728	YDR281C	No agreement	FALSE	1	0
3729	YDR012W	No agreement	FALSE	0	0
3730	YPL129W	No agreement	TRUE	1	0.019
3731	YBR083W	Agreement	TRUE	1	0.006
3732	YNL080C	No agreement	FALSE	1	0.074
3733	YGR030C	No agreement	FALSE	0	0.003
3734	YOL059W	No agreement	FALSE	1	0.001
3735	YDR389W	Agreement	FALSE	1	0
3736	YGL123W	Agreement	TRUE	0	0
3737	YDR081C	No agreement	FALSE	0	0.123
3738	YBR288C	No agreement	FALSE	1	0.119
3739	YGR223C	Agreement	FALSE	1	0
3740	YOL086C	No agreement	FALSE	1	0
3741	YEL064C	Agreement	FALSE	1	0.072
3742	YKL027W	Agreement	FALSE	1	0.208
3743	YJR007W	Agreement	FALSE	0	0.226
3744	YJL076W	No agreement	TRUE	0	0.11
3745	YHR165C	No agreement	FALSE	0	0.155
3746	YLR320W	No agreement	FALSE	1	0.235
3747	YMR142C	Agreement	FALSE	1	0
3748	YDR190C	Agreement	TRUE	0	0.078
3749	YER147C	No agreement	FALSE	0	0.267
3750	YBR283C	No agreement	FALSE	1	0.238
3751	YPL242C	Agreement	FALSE	0	0
3752	YPR131C	No agreement	TRUE	1	0.075
3753	YGL092W	No agreement	TRUE	0	0
3754	YBR087W	No agreement	FALSE	0	0.001
3755	YML022W	Agreement	FALSE	1	0.008
3756	YDL225W	No agreement	TRUE	1	0
3757	YHR206W	No agreement	FALSE	1	0.002
3758	YMR010W	No agreement	FALSE	1	0.19
3759	YKR072C	Agreement	TRUE	1	0.012
3760	YJL174W	No agreement	FALSE	0	0.173
3761	YGR198W	No agreement	FALSE	0	0
3762	YLR426W	No agreement	FALSE	1	0.069
3763	YLR083C	Agreement	FALSE	1	0
3764	YPR104C	No agreement	FALSE	0	0.194
3765	YDL149W	Agreement	FALSE	1	0
3766	YLR316C	No agreement	TRUE	0	0.085
3767	YLR420W	Agreement	FALSE	1	0.007
3768	YPR095C	No agreement	FALSE	1	0.067

3769	YMR067C	Agreement	FALSE	1	0.061
3770	YOL120C	Agreement	FALSE	0	0
3771	YDL108W	No agreement	TRUE	0	0.071
3772	YCL017C	No agreement	FALSE	0	0.002
3773	YLR173W	No agreement	FALSE	1	0.001
3774	YER039C-A	No agreement	FALSE	1	0
3775	YNL212W	Agreement	TRUE	1	0
3776	YBL001C	Agreement	FALSE	1	0.003
3777	YLR137W	Agreement	FALSE	1	0
3778	YLR418C	No agreement	TRUE	1	0.016
3779	YJL091C	Agreement	FALSE	0	0.015
3780	YHR041C	Agreement	FALSE	1	0.02
3781	YJL130C	Agreement	FALSE	1	0.203
3782	YKL060C	Agreement	FALSE	0	0.278
3783	YMR146C	Agreement	TRUE	0	0.001
3784	YGR071C	No agreement	FALSE	1	0.148
3785	YBR081C	Agreement	FALSE	1	0.071
3786	YNL068C	Agreement	TRUE	1	0.008
3787	YHR064C	Agreement	FALSE	1	0.055
3788	YOR267C	Agreement	FALSE	1	0.177
3789	YAL033W	No agreement	FALSE	0	0.003
3790	YNR023W	No agreement	FALSE	1	0.251
3791	YLR131C	Agreement	TRUE	1	0
3792	YLL006W	No agreement	FALSE	1	0.102
3793	YOR061W	No agreement	TRUE	1	0.125
3794	YMR216C	No agreement	FALSE	1	0.056
3795	YML055W	No agreement	FALSE	1	0
3796	YBR246W	No agreement	FALSE	1	0.28
3797	YDR393W	Agreement	TRUE	1	0
3798	YJL074C	Agreement	TRUE	0	0
3799	YKR086W	No agreement	FALSE	0	0.274
3800	YKL215C	Agreement	TRUE	1	0.011
3801	YFL009W	Agreement	FALSE	0	0.03
3802	YKL206C	Agreement	FALSE	1	0.082
3803	YNL317W	No agreement	TRUE	0	0.017
3804	YER019C-A	No agreement	FALSE	1	0.176
3805	YOL139C	Agreement	FALSE	0	0.048
3806	YPL126W	Agreement	TRUE	0	0.072
3807	YGR004W	No agreement	FALSE	1	0.019
3808	YLR182W	Agreement	TRUE	1	0
3809	YDR019C	Agreement	FALSE	1	0.003
3810	YDL019C	Agreement	TRUE	1	0
3811	YLR388W	Agreement	FALSE	1	0
3812	YLR427W	Agreement	TRUE	1	0
3813	YKL190W	Agreement	FALSE	1	0.113
3814	YJL039C	No agreement	FALSE	0	0.215
3815	YMR184W	No agreement	FALSE	1	0.223
3816	YOL021C	Agreement	TRUE	0	0.079
3817	YOR239W	No agreement	TRUE	1	0

3818	YJL189W	Agreement	FALSE	1	0.001
3819	YHR191C	No agreement	FALSE	1	0.257
3820	YEL015W	No agreement	FALSE	1	0.091
3821	YDR233C	No agreement	TRUE	1	0.226
3822	YLR319C	No agreement	FALSE	1	0.093
3823	YCR073W-A	Agreement	FALSE	1	0.009
3824	YKL116C	No agreement	FALSE	1	0.053
3825	YBR176W	Agreement	FALSE	1	0.099
3826	YDL035C	No agreement	TRUE	1	0.001
3827	YNL244C	Agreement	FALSE	0	0.098
3828	YKR042W	No agreement	FALSE	1	0.002
3829	YHR031C	Agreement	FALSE	1	0.237
3830	YNL261W	Agreement	FALSE	0	0.121
3831	YMR061W	Agreement	TRUE	0	0.073
3832	YLR430W	No agreement	TRUE	0	0.055
3833	YKL165C	Agreement	FALSE	0	0
3834	YKL205W	Agreement	TRUE	1	0.002
3835	YPR094W	No agreement	FALSE	0	0.279
3836	YGL216W	No agreement	FALSE	1	0.046
3837	YOR159C	No agreement	TRUE	0	0.138
3838	YNL201C	No agreement	TRUE	1	0.047
3839	YDL140C	Agreement	FALSE	0	0.079
3840	YBR067C	No agreement	TRUE	1	0
3841	YHR205W	No agreement	FALSE	0	0.004
3842	YIL021W	No agreement	TRUE	0	0.046
3843	YLR188W	No agreement	FALSE	1	0.001
3844	YAL026C	Agreement	FALSE	1	0.045
3845	YPL115C	No agreement	FALSE	1	0.041
3846	YOR043W	No agreement	FALSE	1	0.002
3847	YNL130C	No agreement	FALSE	1	0.075
3848	YML097C	Agreement	FALSE	1	0.083
3849	YLR321C	Agreement	FALSE	0	0.092
3850	YOR236W	No agreement	TRUE	0	0.056
3851	YOR288C	No agreement	FALSE	1	0.007
3852	YBR102C	Agreement	TRUE	0	0.259
3853	YOR148C	No agreement	TRUE	0	0
3854	YDL012C	No agreement	FALSE	1	0.004
3855	YNL107W	No agreement	TRUE	1	0.008
3856	YLR447C	Agreement	FALSE	1	0.018
3857	YLR008C	Agreement	FALSE	0	0
3858	YPL019C	No agreement	FALSE	0	0
3859	YDR026C	No agreement	TRUE	1	0.004
3860	YER154W	No agreement	FALSE	1	0
3861	YAR003W	No agreement	FALSE	1	0.023
3862	YJL197W	Agreement	TRUE	1	0.008
3863	YMR145C	Agreement	FALSE	1	0
3864	YHR105W	No agreement	FALSE	1	0
3865	YDL015C	No agreement	FALSE	0	0.235
3866	YOR073W	No agreement	FALSE	1	0.053

3867	YHR143W-A	Agreement	FALSE	1	0.12
3868	YER032W	No agreement	TRUE	1	0
3869	YGR169C	No agreement	TRUE	1	0
3870	YDR090C	No agreement	FALSE	1	0.139
3871	YLR287C-A	Agreement	FALSE	1	0.25
3872	YOR276W	Agreement	FALSE	1	0
3873	YLR211C	Agreement	TRUE	1	0
3874	YJR123W	Agreement	FALSE	0	0.002
3875	YDR002W	No agreement	TRUE	0	0.17
3876	YNR038W	Agreement	FALSE	0	0.044
3877	YJL153C	Agreement	FALSE	1	0
3878	YLR045C	No agreement	TRUE	0	0
3879	YML111W	No agreement	TRUE	1	0.033
3880	YCR012W	Agreement	FALSE	0	0.001
3881	YIL051C	No agreement	FALSE	0	0.135
3882	YLR340W	Agreement	FALSE	0	0.133
3883	YMR223W	No agreement	FALSE	1	0.257
3884	YLR141W	Agreement	TRUE	0	0
3885	YLR380W	Agreement	FALSE	1	0.013
3886	YPL116W	Agreement	FALSE	1	0
3887	YDR500C	Agreement	FALSE	1	0.007
3888	YPL139C	No agreement	FALSE	1	0.044
3889	YLR405W	No agreement	TRUE	1	0.031
3890	YML073C	Agreement	FALSE	0	0
3891	YLR029C	Agreement	TRUE	0	0
3892	YML078W	No agreement	FALSE	1	0
3893	YPL045W	No agreement	FALSE	1	0.148
3894	YDR170C	Agreement	FALSE	0	0.001
3895	YOR238W	No agreement	TRUE	1	0.005
3896	YLR130C	No agreement	FALSE	1	0.2
3897	YCR008W	No agreement	FALSE	1	0
3898	YOR233W	No agreement	TRUE	1	0.002
3899	YFL017C	No agreement	TRUE	0	0.001
3900	YOR115C	No agreement	TRUE	1	0
3901	YPR157W	No agreement	FALSE	1	0.056
3902	YNL045W	Agreement	FALSE	1	0
3903	YJL051W	No agreement	FALSE	1	0
3904	YJL192C	No agreement	FALSE	1	0
3905	YBL005W	No agreement	FALSE	1	0.008
3906	YKL005C	No agreement	TRUE	1	0.001
3907	YPL148C	Agreement	FALSE	1	0
3908	YFL037W	No agreement	TRUE	0	0
3909	YPR004C	No agreement	FALSE	1	0
3910	YDR382W	Agreement	FALSE	1	0
3911	YPL235W	Agreement	TRUE	0	0.067
3912	YER057C	No agreement	FALSE	1	0.19
3913	YOL066C	Agreement	TRUE	0	0.086
3914	YEL043W	No agreement	FALSE	1	0.027
3915	YPL041C	Agreement	FALSE	1	0.005

3916	YOR168W	Agreement	TRUE	0	0.033
3917	YMR253C	Agreement	FALSE	1	0.018
3918	YLR354C	Agreement	FALSE	1	0.001
3919	YOR311C	No agreement	TRUE	1	0.002
3920	YNR003C	No agreement	TRUE	0	0.087
3921	YJL065C	No agreement	FALSE	1	0.091
3922	YPL232W	No agreement	FALSE	1	0.137
3923	YGL190C	Agreement	FALSE	0	0
3924	YJL179W	No agreement	TRUE	1	0.278
3925	YIL161W	No agreement	FALSE	1	0.217
3926	YIL134W	No agreement	FALSE	1	0
3927	YOL130W	No agreement	FALSE	0	0.045
3928	YKR028W	No agreement	FALSE	1	0.249
3929	YDR050C	Agreement	TRUE	0	0.027
3930	YKL024C	Agreement	FALSE	0	0.007
3931	YHL007C	Agreement	TRUE	1	0.013
3932	YMR094W	Agreement	FALSE	0	0.003
3933	YCR086W	No agreement	TRUE	1	0.001
3934	YMR148W	No agreement	FALSE	1	0
3935	YLR421C	Agreement	TRUE	1	0.029
3936	YCR106W	No agreement	FALSE	1	0.188
3937	YEL034W	No agreement	FALSE	0	0.083
3938	YBL057C	No agreement	FALSE	1	0
3939	YOL044W	No agreement	FALSE	1	0
3940	YKL056C	Agreement	FALSE	1	0
3941	YBR187W	No agreement	FALSE	1	0.001
3942	YJR138W	No agreement	FALSE	0	0.236
3943	YIL126W	No agreement	TRUE	0	0.035
3944	YIL003W	No agreement	FALSE	0	0.009
3945	YMR095C	No agreement	FALSE	1	0.003
3946	YML121W	No agreement	TRUE	1	0.013
3947	YML046W	No agreement	FALSE	0	0.04
3948	YCR048W	No agreement	FALSE	1	0.001
3949	YOR210W	Agreement	TRUE	0	0.092
3950	YPL180W	No agreement	TRUE	1	0.016
3951	YLR003C	No agreement	TRUE	1	0.256
3952	YLR005W	No agreement	TRUE	0	0.024
3953	YFR038W	No agreement	TRUE	1	0.001
3954	YPL277C	Agreement	TRUE	0	0
3955	YDL074C	No agreement	FALSE	1	0.107
3956	YOL029C	Agreement	TRUE	1	0
3957	YDR322C-A	No agreement	FALSE	0	0
3958	YDR311W	No agreement	FALSE	0	0
3959	YDR013W	No agreement	FALSE	0	0.132
3960	YPL249C-A	Agreement	TRUE	1	0
3961	YBR109C	Agreement	FALSE	0	0
3962	YOR335C	Agreement	FALSE	0	0.002
3963	YPL210C	Agreement	TRUE	0	0.039
3964	YDR037W	Agreement	FALSE	0	0.019

3965	YMR127C	No agreement	TRUE	1	0.002
3966	YLR147C	No agreement	TRUE	0	0.028
3967	YMR243C	No agreement	TRUE	1	0.015
3968	YGR089W	No agreement	FALSE	1	0.201
3969	YJL196C	Agreement	FALSE	1	0.016
3970	YCL011C	No agreement	FALSE	1	0.101
3971	YGR031W	Agreement	TRUE	1	0
3972	YLR359W	Agreement	FALSE	0	0
3973	YBR011C	Agreement	FALSE	0	0.154
3974	YOR047C	Agreement	FALSE	1	0.131
3975	YGL021W	Agreement	FALSE	1	0
3976	YDR507C	Agreement	TRUE	1	0
3977	YKR053C	No agreement	TRUE	1	0
3978	YML106W	Agreement	FALSE	1	0
3979	YGL169W	Agreement	FALSE	0	0.007
3980	YDR448W	No agreement	FALSE	1	0.147
3981	YLR249W	Agreement	FALSE	0	0
3982	YGR277C	No agreement	TRUE	0	0.005
3983	YPR187W	Agreement	TRUE	0	0.024
3984	YIL052C	Agreement	FALSE	1	0
3985	YDR027C	Agreement	FALSE	1	0
3986	YNL135C	No agreement	FALSE	1	0.016
3987	YGR037C	Agreement	FALSE	1	0.143
3988	YLR228C	No agreement	FALSE	1	0
3989	YDR263C	No agreement	TRUE	1	0.001
3990	YBR265W	Agreement	FALSE	0	0.065
3991	YNR030W	No agreement	FALSE	1	0.04
3992	YNL029C	Agreement	TRUE	1	0.114
3993	YBR095C	No agreement	FALSE	1	0.266
3994	YGR118W	Agreement	FALSE	1	0.003
3995	YOL155C	Agreement	TRUE	1	0
3996	YKL163W	No agreement	FALSE	1	0
3997	YNL304W	Agreement	FALSE	1	0.265
3998	YDR531W	No agreement	TRUE	0	0.121
3999	YPR034W	Agreement	FALSE	0	0.155
4000	YOR383C	No agreement	TRUE	1	0
4001	YER023W	No agreement	FALSE	0	0
4002	YJR144W	No agreement	TRUE	1	0.004
4003	YLR384C	Agreement	TRUE	1	0.093
4004	YNL302C	Agreement	FALSE	1	0.027
4005	YLR353W	Agreement	FALSE	1	0.008
4006	YPR029C	Agreement	TRUE	1	0.074
4007	YJL128C	No agreement	FALSE	1	0.291
4008	YDR212W	No agreement	FALSE	0	0.124
4009	YHR069C	Agreement	TRUE	0	0.034
4010	YOR189W	No agreement	TRUE	1	0.038
4011	YDR280W	No agreement	TRUE	0	0.204
4012	YKL104C	Agreement	FALSE	0	0.143
4013	YLR109W	Agreement	FALSE	1	0

4014	YKL063C	No agreement	FALSE	1	0.234
4015	YGL119W	No agreement	TRUE	0	0.031
4016	YMR212C	No agreement	FALSE	0	0.271
4017	YDL106C	Agreement	TRUE	0	0
4018	YPL208W	Agreement	FALSE	1	0.001
4019	YBR091C	No agreement	FALSE	0	0
4020	YER031C	No agreement	FALSE	1	0.223
4021	YOR063W	Agreement	FALSE	0	0
4022	YDR206W	Agreement	FALSE	1	0.203
4023	YMR149W	No agreement	FALSE	0	0.256
4024	YDL089W	Agreement	TRUE	1	0.183
4025	YNR046W	Agreement	FALSE	0	0.076
4026	YJR017C	No agreement	FALSE	0	0.061
4027	YBR263W	No agreement	TRUE	1	0.005
4028	YLR396C	Agreement	FALSE	1	0.175
4029	YDR424C	Agreement	TRUE	1	0
4030	YMR235C	Agreement	FALSE	0	0.262
4031	YJR069C	Agreement	FALSE	1	0.002
4032	YBR260C	Agreement	FALSE	1	0.058
4033	YLL013C	No agreement	FALSE	1	0.066
4034	YLL050C	Agreement	FALSE	0	0.001
4035	YDR302W	Agreement	FALSE	0	0.021
4036	YPL092W	No agreement	FALSE	1	0
4037	YCL045C	Agreement	FALSE	1	0.214
4038	YMR244C-A	Agreement	TRUE	1	0
4039	YNR048W	No agreement	FALSE	1	0
4040	YOR045W	No agreement	FALSE	1	0
4041	YPL231W	Agreement	FALSE	0	0.013
4042	YOR306C	No agreement	FALSE	1	0.093
4043	YKR057W	Agreement	FALSE	1	0.002
4044	YFR031C-A	Agreement	TRUE	1	0.001
4045	YPL134C	Agreement	FALSE	1	0.001
4046	YJL056C	Agreement	FALSE	1	0.061
4047	YGL106W	No agreement	TRUE	0	0.015
4048	YPR141C	No agreement	TRUE	1	0
4049	YMR312W	No agreement	FALSE	1	0.01
4050	YBL008W	No agreement	TRUE	1	0
4051	YOR326W	No agreement	TRUE	0	0
4052	YPL262W	Agreement	FALSE	1	0
4053	YOR167C	Agreement	FALSE	1	0.001
4054	YDR267C	No agreement	TRUE	0	0
4055	YKL156W	Agreement	FALSE	1	0.003
4056	YDR359C	Agreement	TRUE	1	0
4057	YAL007C	Agreement	FALSE	1	0
4058	YGL176C	Agreement	FALSE	1	0.024
4059	YOL146W	Agreement	FALSE	0	0.188
4060	YML113W	Agreement	FALSE	1	0.07
4061	YML065W	No agreement	TRUE	0	0.023
4062	YJR107W	Agreement	FALSE	1	0.01

4063	YOL092W	No agreement	FALSE	1	0.002
4064	YPL195W	No agreement	FALSE	1	0.076
4065	YOR360C	No agreement	FALSE	1	0.088
4066	YNR058W	Agreement	FALSE	1	0
4067	YLR291C	No agreement	FALSE	0	0.011
4068	YDL129W	No agreement	FALSE	1	0.001
4069	YHL034C	Agreement	TRUE	1	0.001
4070	YLR039C	Agreement	TRUE	1	0.006
4071	YKL220C	No agreement	FALSE	1	0.089
4072	YDL018C	Agreement	FALSE	1	0.001
4073	YDL130W	Agreement	TRUE	1	0.007
4074	YML012W	Agreement	FALSE	1	0.016
4075	YOR274W	Agreement	TRUE	1	0.001
4076	YDR188W	No agreement	TRUE	0	0.033
4077	YDL160C	Agreement	FALSE	1	0.005
4078	YIL043C	No agreement	FALSE	1	0.028
4079	YGR024C	No agreement	FALSE	0	0.209
4080	YBR010W	Agreement	FALSE	1	0
4081	YEL003W	No agreement	FALSE	1	0.269
4082	YBR254C	No agreement	FALSE	0	0.09
4083	YKL122C	No agreement	FALSE	0	0.285
4084	YLL033W	Agreement	FALSE	1	0.018
4085	YHR201C	No agreement	TRUE	0	0.102
4086	YCR088W	Agreement	TRUE	1	0
4087	YPR088C	No agreement	FALSE	0	0.119
4088	YBL027W	Agreement	FALSE	1	0
4089	YBR239C	No agreement	FALSE	1	0.255
4090	YOL133W	Agreement	TRUE	0	0
4091	YDR245W	No agreement	TRUE	1	0
4092	YOR382W	No agreement	FALSE	1	0
4093	YMR004W	Agreement	FALSE	1	0.167
4094	YCL031C	No agreement	TRUE	0	0.275
4095	YOL026C	Agreement	FALSE	0	0
4096	YKL006W	Agreement	FALSE	1	0.007
4097	YLR116W	No agreement	FALSE	0	0
4098	YBL087C	Agreement	FALSE	1	0.003
4099	YER148W	Agreement	TRUE	0	0.089
4100	YDR510W	No agreement	TRUE	0	0
4101	YDL166C	Agreement	TRUE	0	0.018
4102	YLR200W	No agreement	TRUE	1	0.168
4103	YKL210W	Agreement	FALSE	0	0
4104	YJR048W	Agreement	FALSE	1	0
4105	YER047C	No agreement	TRUE	1	0.009
4106	YLR221C	Agreement	TRUE	1	0.104
4107	YDL122W	Agreement	TRUE	1	0.011
4108	YOR078W	Agreement	TRUE	1	0.043
4109	YLR381W	Agreement	FALSE	1	0
4110	YKL017C	Agreement	TRUE	1	0.069
4111	YJL186W	No agreement	FALSE	1	0.013

4112	YMR130W	No agreement	FALSE	1	0.287
4113	YOR327C	No agreement	TRUE	1	0.002
4114	YNL031C	Agreement	FALSE	1	0
4115	YML053C	Agreement	FALSE	1	0.153
4116	YLL045C	Agreement	FALSE	1	0
4117	YNL119W	No agreement	TRUE	1	0.023
4118	YJL123C	No agreement	TRUE	1	0.014
4119	YNL098C	Agreement	TRUE	1	0
4120	YNL208W	No agreement	FALSE	1	0
4121	YDR310C	Agreement	TRUE	1	0
4122	YDR097C	Agreement	TRUE	1	0
4123	YMR054W	Agreement	FALSE	1	0
4124	YGR040W	No agreement	FALSE	1	0
4125	YCL063W	Agreement	FALSE	1	0
4126	YBL002W	No agreement	FALSE	1	0
4127	YHR167W	No agreement	FALSE	1	0.006
4128	YOL128C	Agreement	FALSE	1	0
4129	YBR191W	Agreement	FALSE	1	0
4130	YGL178W	No agreement	FALSE	0	0.007
4131	YJR119C	Agreement	TRUE	1	0.002
4132	YKL010C	Agreement	FALSE	1	0.082
4133	YDR498C	No agreement	FALSE	0	0.079
4134	YNL165W	No agreement	TRUE	1	0.064
4135	YCR023C	Agreement	FALSE	1	0.001
4136	YBR203W	Agreement	FALSE	1	0
4137	YDL083C	Agreement	FALSE	1	0
4138	YMR215W	Agreement	TRUE	1	0
4139	YMR251W-A	No agreement	TRUE	1	0.03
4140	YPL252C	No agreement	FALSE	0	0
4141	YNL225C	Agreement	TRUE	1	0.001
4142	YNL069C	Agreement	FALSE	1	0
4143	YPL103C	No agreement	TRUE	1	0
4144	YDR410C	No agreement	FALSE	1	0.002
4145	YIL131C	No agreement	FALSE	0	0
4146	YOR283W	No agreement	TRUE	1	0.039
4147	YDL184C	No agreement	FALSE	1	0.077
4148	YMR262W	No agreement	FALSE	1	0
4149	YPR031W	Agreement	TRUE	1	0.026
4150	YDR104C	No agreement	FALSE	1	0.025
4151	YCL047C	No agreement	FALSE	1	0.07
4152	YAL005C	No agreement	TRUE	1	0.015
4153	YML124C	No agreement	TRUE	1	0
4154	YDL192W	Agreement	FALSE	1	0
4155	YBL104C	No agreement	FALSE	1	0.065
4156	YBR106W	Agreement	FALSE	1	0
4157	YHR086W	Agreement	TRUE	1	0
4158	YNL322C	No agreement	FALSE	1	0.013
4159	YJR014W	No agreement	TRUE	1	0.009
4160	YAL003W	Agreement	FALSE	0	0

4161	YDR414C	No agreement	FALSE	1	0
4162	YLR355C	Agreement	FALSE	0	0.002
4163	YML010W	No agreement	FALSE	0	0.094
4164	YCR034W	Agreement	FALSE	1	0
4165	YKR029C	Agreement	FALSE	1	0.088
4166	YER149C	No agreement	TRUE	1	0.002
4167	YDR198C	No agreement	FALSE	1	0.05
4168	YOR305W	No agreement	TRUE	1	0.003
4169	YJR131W	No agreement	FALSE	1	0.054
4170	YLR146C	No agreement	FALSE	1	0.002
4171	YMR036C	Agreement	TRUE	1	0
4172	YKL124W	Agreement	TRUE	1	0
4173	YMR002W	Agreement	FALSE	1	0
4174	YLR344W	Agreement	FALSE	1	0
4175	YNL041C	No agreement	FALSE	1	0.126
4176	YEL027W	No agreement	FALSE	1	0.114
4177	YML063W	Agreement	FALSE	1	0
4178	YGR012W	No agreement	TRUE	1	0.021
4179	YGR148C	Agreement	FALSE	1	0
4180	YKL004W	No agreement	FALSE	0	0.248
4181	YNL325C	No agreement	FALSE	1	0.093
4182	YLR419W	No agreement	TRUE	0	0.027
4183	YLR285W	Agreement	TRUE	1	0.075
4184	YBR175W	No agreement	FALSE	1	0.023
4185	YGR261C	Agreement	TRUE	1	0.012
4186	YEL024W	Agreement	TRUE	1	0
4187	YLR075W	Agreement	FALSE	0	0.065
4188	YLR389C	Agreement	FALSE	1	0.008
4189	YOR247W	No agreement	TRUE	1	0
4190	YGR068C	Agreement	FALSE	1	0.037
4191	YIL044C	No agreement	FALSE	1	0.003
4192	YMR134W	Agreement	FALSE	0	0.156
4193	YLR412W	No agreement	TRUE	1	0.002
4194	YMR236W	No agreement	TRUE	0	0.001
4195	YNL310C	No agreement	TRUE	0	0.075
4196	YDR064W	Agreement	FALSE	0	0
4197	YNL209W	Agreement	FALSE	0	0
4198	YJR092W	Agreement	TRUE	1	0
4199	YCR020W-B	No agreement	TRUE	1	0.024
4200	YAL038W	Agreement	TRUE	0	0
4201	YJL062W	Agreement	TRUE	1	0.056
4202	YJL138C	Agreement	FALSE	1	0.002
4203	YGL236C	Agreement	FALSE	1	0
4204	YML109W	Agreement	TRUE	1	0
4205	YDL207W	Agreement	TRUE	0	0.006
4206	YKL088W	No agreement	FALSE	0	0.144
4207	YMR102C	No agreement	FALSE	1	0
4208	YKR054C	No agreement	FALSE	1	0.004
4209	YJR148W	No agreement	FALSE	1	0

4210	YBL069W	No agreement	FALSE	1	0.025
4211	YAL041W	Agreement	TRUE	0	0.023
4212	YML127W	No agreement	FALSE	0	0
4213	YAR002C-A	No agreement	FALSE	1	0
4214	YMR129W	No agreement	FALSE	1	0.139
4215	YOR265W	Agreement	FALSE	1	0.228
4216	YML006C	No agreement	TRUE	1	0.174
4217	YMR153W	No agreement	TRUE	1	0.043
4218	YML028W	Agreement	FALSE	1	0.002
4219	YPR051W	No agreement	FALSE	1	0.118
4220	YML074C	No agreement	TRUE	1	0.004
4221	YPL155C	No agreement	TRUE	1	0
4222	YML058W	Agreement	TRUE	1	0.002
4223	YDR356W	No agreement	TRUE	0	0.003
4224	YOR048C	No agreement	TRUE	0	0.004
4225	YGL100W	No agreement	FALSE	0	0.001
4226	YLR105C	Agreement	TRUE	0	0.005
4227	YKL152C	Agreement	TRUE	0	0.047
4228	YCL068C	Agreement	FALSE	0	0.209
4229	YGR192C	No agreement	FALSE	1	0.073
4230	YKL018W	Agreement	FALSE	0	0.278
4231	YOR084W	Agreement	FALSE	1	0
