# Supplementary material for: Adaptation of Organisms by Resonance of RNA Transcription with the Cellular Redox Cycle
Source: PLoS One. 2011 Sep 28;6(9):e25270. doi: 10.1371/journal.pone.0025270 (PMC3182209; doi:10.1371/journal.pone.0025270)
Supplement: Appendix S1 — (DOC) [file pone.0025270.s014.doc]

**Supporting Online Material**

This supplement contains:

Material and methods
Supplementary web file
Figs. S1 to S9
Tables S1 to S3
References

**Materials and Methods**

**Microarray Data Analysis**

Preprocessed normalized *log*-transformed intensity data for *S. cerevisiae* transcriptome was obtained from Gene Expression Omnibus database (GEO), the yeast metabolic cycle (YMC), Series GSE3431 (3). Related study of redox cycling in *S. cerevisiae*, the transcriptional redox attractor cycle **(**TRAC), Series GSE2583 (4), was obtained from GEO. TRAC expression data was normalized by adjustment of time-series expressions for differences in hybridization using biotinylated standards and a polynomial fit (4), after which it was *log*-transformed. Correspondence between YMC and TRAC redox cycles was found by maximizing the sum of pairwise cross-correlation coefficients between two expression time-series for each gene (normalized between 0 and 1). Because the periods of the two cycles differ, i.e. 40 minutes period for TRAC and 4 to 5 hours period for YMC, we aligned the time points as follows: points 8-12 of YMC were aligned to points 1-4 of TRAC (oxidative phase, renumbered as points 1-4 for YMC), and 1-7 of YMC to points 5-11(or 10 for the first cycle) of TRAC (reductive phase, renumbered as points 5-12 for YMC) in all three cycles considered. The reductive and oxidative phases were identified by grouping factor levels (time points) into two groups chosen based on minimization of the model deviance specified by Akaike's information criterion (AIC) (5). Additionally, similar split between reductive and oxidative phase was obtained using the results of Tuckey’s all-pair wise time points comparison test. Two expression profiles for the same gene were considered in agreement between two data sets when the cross-correlation coefficient was significantly large (> 0.5) and the cross-correlation coefficient maxima were close together (≤ 3 points apart). These thresholds were chosen based on agreement between gene expression profiles for all genes. Altogether 4,231 ORFs were in agreement between YMC and TRAC. YMC normalized data was chosen for further analysis due to its higher expression profile consistency among the three cycles. Codon adaptation index, phylogenetic similarity values, frequencies of sequence variation were attributed to the specific time-points and genes were considered expressed if normalized gene expression (between 0 and 1) was above 0.5. Other thresholds were considered and resulted in similar oscillations of all values.

**Periodicity and viability**

Periodicity was assessed with Fisher’s Exact g test for multiple time series with false discovery rate (*fdr*) correction (6) (*fdr* < 0.05) in addition to checking that one of the three dominating oscillation frequencies of gene expression was consistent with the period of dissolved oxygen oscillation. The check was performed using fast Fourier transform analysis (FFT) (7). Viability designation (*i.e.* non-essential *vs.* essential genes) was obtained from the Saccharomyces Genome Database (SGD) under phenotype properties at <http://www.yeastgenome.org/cgi-bin/search/featureSearch>. Accessed 2010, December 1.

**Codon usage bias and sequence similarity**

For each gene, codon usage bias or codon adaptation index (CAI) was estimated using CODONW^8^. Correspondence analysis (COA) (8) was employed to identify a codon reference set that explains the largest trend of codon usage variation across *S. cerevisiae* genome, Saccharomyces genus stricto clade (*S. paradoxus, S. mikatae, S. kudriavzevii, S. bayanus, S. castellii, S. kluyvery)*, and H. sapiens genome. Additionally, other available codon usage indices were used as complementary methods to confirm the result of oscillating codon bias in phase with the redox cycle (data not shown). Specifically, Frequency of Optimal Codons (16), Codon Bias Index (17) and The Effective Number of Codons (18) were used. *Saccharomyces sensu stricto* clade genes and *H. sapiens* protein-coding genes that are phylogenetically similar to *S. cerevisiae* ORFs were determined using blast search of *S. cerevisiae* ORFs against FASTA protein coding sequences obtained from the National Center for Biotechnology Information (NCBI) (blast *e*-value < 10^-50^). Additionally, Codon Bias Index, Frequency of Optimal Codons, and The Effective Number of Codons (8) were used as independent methods for computing codon bias and generated the same results as CAI (data not shown). To restrict the BLAST analysis results to orthologous ORFs, a stringent criterion was used: significance level blast *e*-value < 10^-50^, a minimum of 50% homology with yeast species, or a minimum of 40% homology with human proteins, and a cut-off against accepting multiple best hits, such that only the best single match was used for each *S. cerevisiae* ORF.

# Frequency of sequence variation

Starting and ending coordinates for ORFs and introns were obtained from Saccharomyces Genome Database (SGD) (9), while 5’ and 3’ UTR coordinates from SGD were verified with (10). Coordinates of un-transcribed regions were identified using overlap between absence of transcribed elements in SGD, absence of 5’ and 3’ UTRs (9), presence of 36-mers mapped to regions with nearly absent expression levels (11) and the presence of non-coding regions (12). Sequence variations for each genomic element were counted in 63 *S. cerevisiae* strains whole-genome single nucleotide polymorphisms (SNPs) data (13), and normalized by the gene length and number of strains. The mean and confidence intervals of the frequency of sequence variation ratios between transcribed elements (ORFs, introns, 5’ and 3’UTRs) and un-transcribed regions were estimated by a non-parametric bootstrap procedure (14). Along with the data measured at the points of chemical extrema, i.e. at the infection point of the transition between high and low residual dissolved oxygen (oxidative point), and at the inflection point of the transition between low and high accumulated dissolved hydrogen sulfide (reductive point) the values measured across the chemical potential, i.e. the time interval between the extrema, were combined.

**Statistical analysis:**

Kolmogorov-Smirnov test and Q-Q plots showed that distributions of CAI, similarity, log-transformed expressions, and frequencies of sequence variation were non-normally distributed. Variance was compared using two-tailed Fisher’s F-test. Non-parametric two-tailed and one-tailed Wilcoxon rank sum test was used to test the quality of the two median values with non-normal errors for the oxidative and reductive phases. The significance was examined at the 5% level for all statistical tests employed. Complete enumeration of subsets of model parameters was used to determine which parameters explain CAI regression. The best fit was found minimizing the Bayesian information criterion (BIC) (15). The model with the smallest BIC that corresponds to the model with maximum posterior probability was chosen. The BIC was used since in comparison with AIC it often selects more parsimonious models (15).

**Supplementary data file**

ORFs that were expressed periodically and aperiodically during each measured time interval in the redox cycle of both data sets are provided in a Supplementary data file. Within the periodic ORF set, 2,768 out of 3,614 ORFs (~76.6%) were non-essential, and 846 (~23.4%) were essential. Similar distribution was observed for non-periodic genes: ~72.8% non-essential and ~27.2 % essential. 846/1,014 of the essential genes (~83.4%) were periodic, and 2,768/3,217 of the non-essential genes (~86.0%) were periodic.

**Supplementary Figures and Tables**

The mean CAI values vary from 0.369±0.140(SD, N=4055) during the oxidative phase, to 0.337±0.099(SD, N=8799) during the reductive phase for non-essential genes. Similarly, for essential genes, the mean CAI values vary from 0.395±0.150(SD, N=1454) to 0.364±0.130(SD, N=1932) for the oxidative and reductive phases, respectively. The values given for the second cycle were chosen as representative. Similar oscillations were observed in all three cycles, **Table S1**. Using variance test, *i.e.* Fisher’s F-test, we also found that the ratio of variances of CAI value distributions during oxidative *vs.* reductive phase is ~2.0 for non-essential genes (*p*-value < 2.2x10^-16^) and is ~1.5 for essential genes (*p*-value =10^-7^), which coincides with a larger variance of amplitude in RNA transcripts during the oxidative *vs*. reductive phase (**Table S1** and **Figure S6**).

Additionally, we examined CAI values in *Saccharomyces sensu stricto* clade (*S. paradoxus, S. mikatae, S. kudriavzevii, S. bayanus, S. castellii, S. kluyvery*) genes and in human protein-coding genes that are similar in their amino acid sequence coding potential to *S. cerevisiae* ORFs (Blast *e*-value <10^-50^). The observed mean difference in CAI values between the oxidative and reductive phases for the *sensu stricto* gene homologues expressed at each corresponding phase is ~0.01, with *p*-value 10^-10^ (**fig. S1, Table** **S1**), while the same difference for human homologues is ~0.03 with *p*-value 10^-9^ (**fig. S2, Table S1**). Presumably, the larger variance in CAI values for human gene homologues is a result of a larger variance in the amplitude of RNA transcription during the oxidative phase in human cells *vs*. in the *Saccharomyces sensu stricto* clade.

Mean CAI values between the oxidative and reductive phases for the *sensu stricto* gene homologues expressed at each corresponding phase are shown in **Figure S1**, and for the *H. sapiens* gene homologues are depicted in **Figure S2**. The mean pair wise phylogenetic similarity values between *H. sapiens* genes and *S. cerevisiae* redox cycle genes are shown in **Figure S3**. **Figure S4** shows the relationship between the frequency of sequence variation in 5’UTRs and 3’UTRs expressed during the redox cycle of yeast *S. cerevisiae*. **Figure S5** shows oscillation of the number and the average length of *S. cerevisiae* introns, 5’, and 3’UTRs . **Figure S6** shows oscillation of the mean and total log-normalized RNA expression values for *S. cerevisiae* during redox cycle. **Figure S7** shows oscillation of mean ORF length and **Figure S8** shows oscillations of third base in synonymous codons. **Figure S9** shows the result of modeling the relationship between CAI values and the total log-normalized RNA expression level in the oxidative phase for each gene in *S. cerevisiae*.

Mean CAI values for *S. cerevisiae* and *Saccharomyces sensu stricto* and their standard deviations for all of the three cycles are given in **Table S1**. **Table S2** details mean pair wise ORF sequence similarity comparison between *S. cerevisiae* and all ORFs in the *Saccharomyces* *sensu stricto* group, and between *S. cerevisiae* and all *H. sapiens* ORFs matching the period of the redox cycle for all of the three cycles. The mean frequencies of sequence variation for *S. cerevisiae* ORFs, introns, 5’ and 3’ UTRs, and their corresponding standard deviations for all the cycles are given in **Table S3**. Two-tailed Wilcoxon rank sum test was used to test equality of the median values between the oxidative and reductive phases of the cycle.

Interestingly, there are more introns encoded in genes expressed during the oxidative rather than the reductive phase (**fig. S5 A**). In addition, introns expressed during the oxidative phase have longer lengths. In contrast, the length of intron-coding ORFs is shorter for genes transcribed during the oxidative phase *vs*. the reductive phase (**fig. S7**). In addition, the average lengths of 5’ and 3’UTRs are also reduced in the oxidative phase, however their numbers were oscillating consistently with the number of ORFs (**fig. S5 B and C**). Introduction of introns into non-essential genes, possibly at the expense of ORF lengths and the lengths of 5’ and 3’UTRs, may be consistent with a possible conversion of non-essential genes into essential function genes. Thus, the intron number and length correlate with CAI values, suggesting that the oxidizing environment favors intron propagation and maintenance.

To identify a statistical model that best explains variation in CAI values, a complete enumeration of subsets of relevant model parameters was performed. The model parameters that were considered included the total RNA transcript level during both phases and individually, minimum RNA transcript level during the cycle (the baseline), GC content, and gene length. The total RNA transcript level during the oxidative phase together with the baseline amplitude of RNA transcripts for each gene resulted in the best-fit model explaining 51% and 58% of total variance of CAI values for non-essential and essential genes, respectively (Materials and Methods section). The total RNA transcript levels during the oxidative phase alone explained 49% and 57% of total CAI variance among non-essential and essential genes, respectively (**fig.** **S9**).


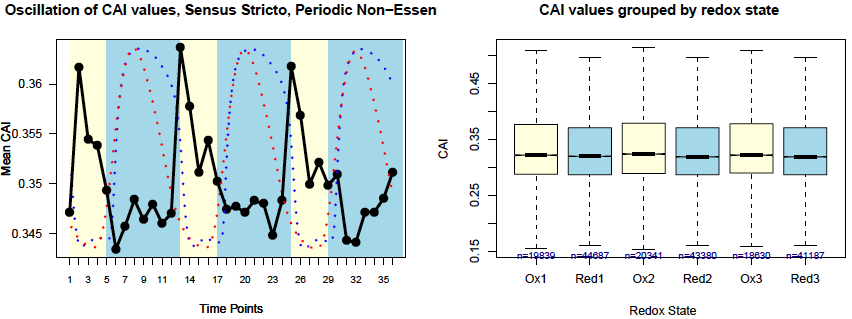


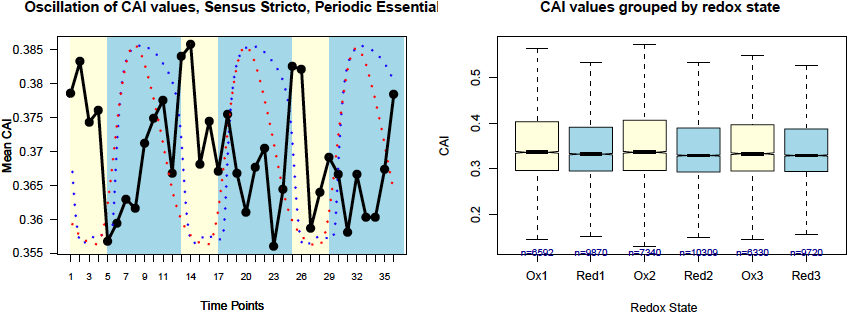


**Fig. S1** The relationship between dissolved oxygen (blue curve), hydrogen sulfide (red curve) (from reference 1), codon adaptation index (CAI, black line) of *sensu stricto* clade genes (*S. paradoxus, S. mikatae, S. kudriavzevii, S. bayanus, S. castellii, S. kluyvery*) similar in amino acid sequences to *S. cerevisiae* ORFs expressed periodically during the redox cycle. Yellow color (time points 1-4) is the oxidative phase, and the blue color (time points 5-12) is the reductive phase. Boxes show median values with statistical significance with notches (if two boxes' notches do not overlap this is a ‘strong evidence’ that their medians differ (2)), first quantile (25%) and third quantile (75%); whiskers indicate minimum and maximum values.


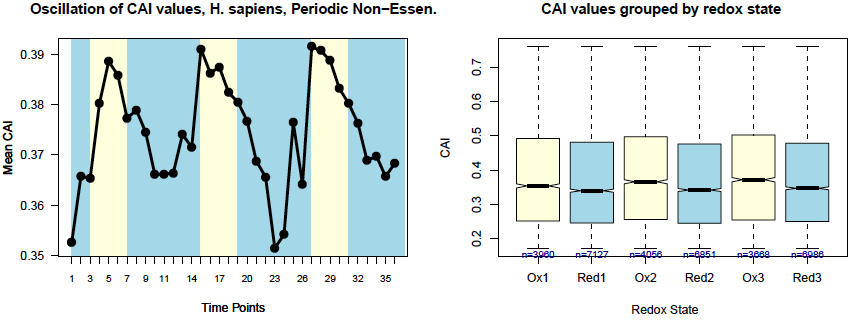


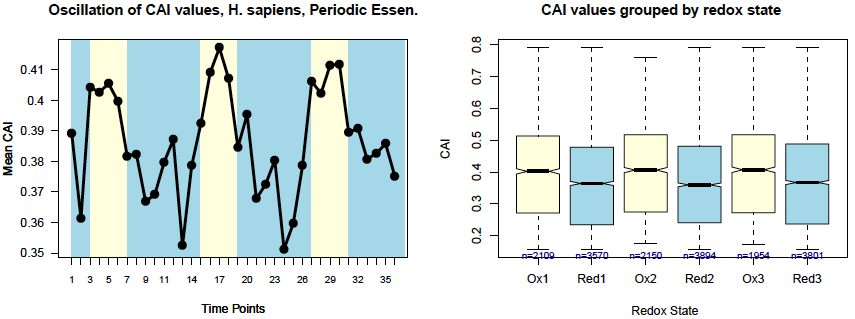


**Fig. S2** The relationship between codon adaptation index (CAI, black line) for *H. sapiens* genes that are similar in amino acid sequence to *S. cerevisiae* genes expressed periodically in the redox cycle. Yellow color (time points 3-6) is the suggested oxidative phase, and the blue color (time points 1-2 and 7-12) is the suggested reductive phase. Boxes show median values with statistical significance with notches (if two boxes' notches do not overlap this is a ‘strong evidence’ that their medians differ (2)), first quantile (25%) and third quantile (75%); whiskers indicate minimum and maximum values.


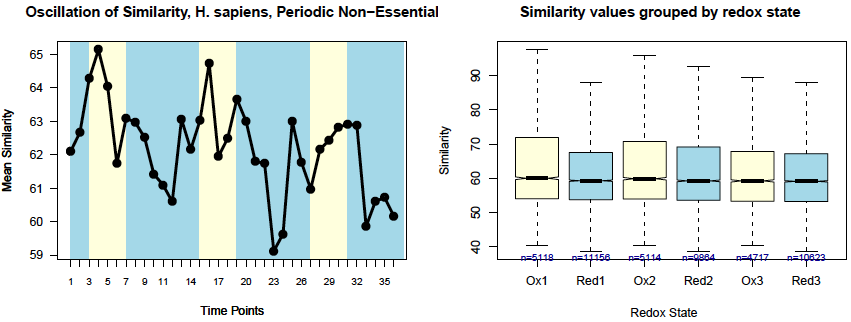


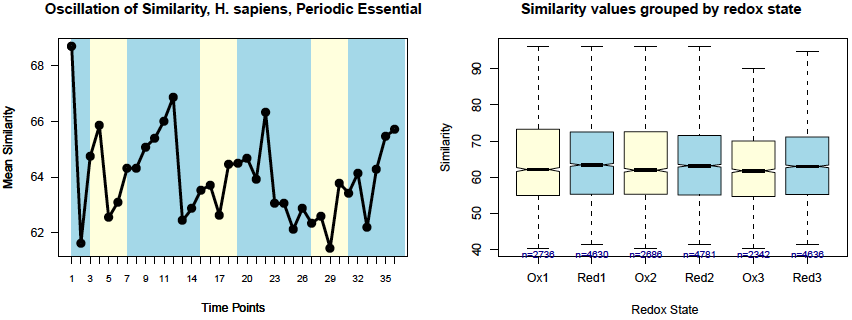


**Fig. S3** The relationship between sequence similarity of *H. sapiens* genes to *S. cerevisiae* genes periodically expressed in the redox cycle. Yellow color (time points 3-6) is the suggested oxidative phase, and the blue color (time points 1-2 and 7-12) is the suggested reductive phase. Boxes show median values with statistical significance with notches (if two boxes' notches do not overlap this is a ‘strong evidence’ that their medians differ (2)), first quantile (25%) and third quantile (75%); whiskers indicate minimum and maximum values.

**
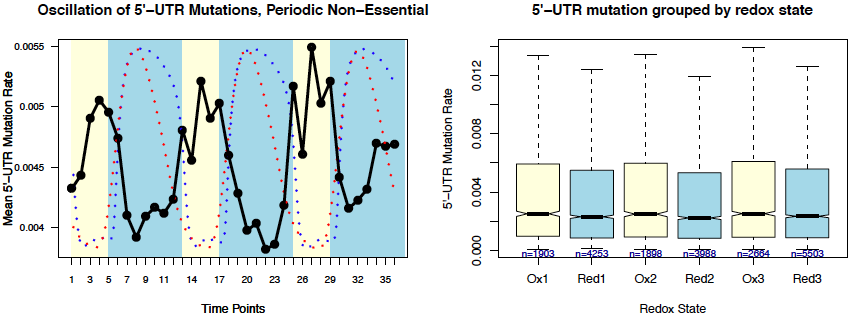
**

**
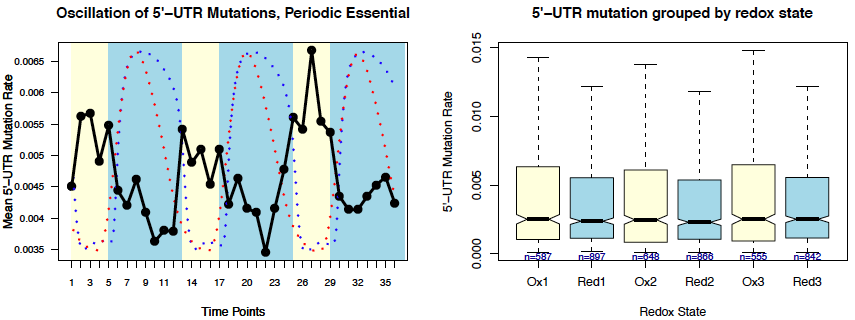
**

**
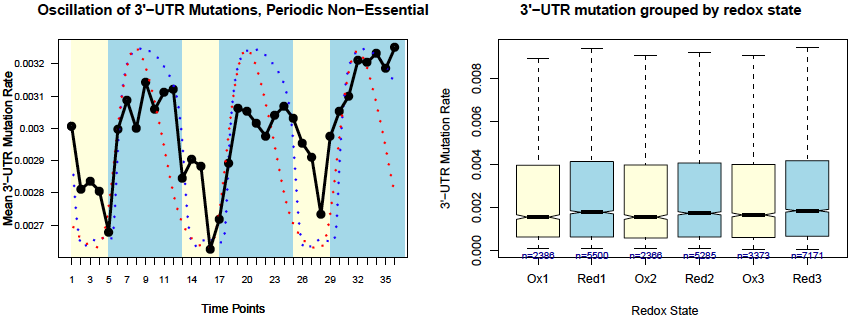
**

**
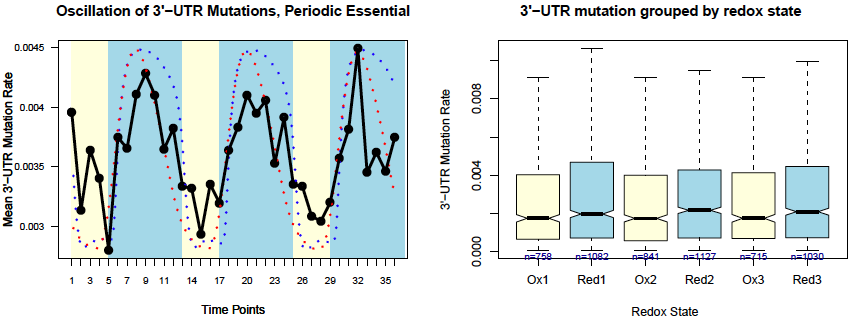
**

**Fig. S4** The relationship between dissolved oxygen (blue dots), hydrogen sulfide (red dots) (from reference 1), and the frequency of sequence variation during the redox cycle of yeast *S. cerevisiae*. The opposite phased oscillations in the frequency of sequence variation values in 5’UTRs and 3’UTRs (black lines) match the period of the fluctuations in the redox states.


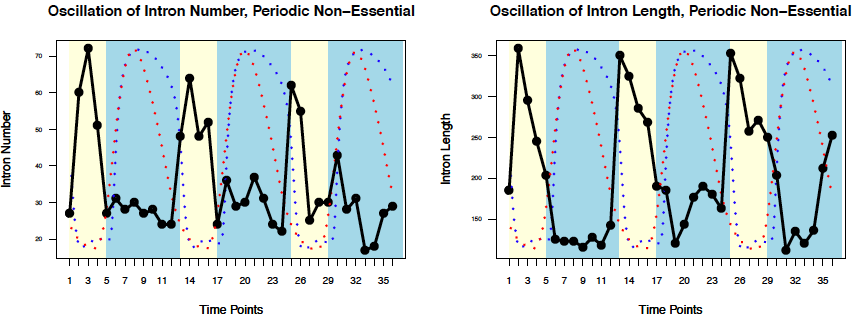


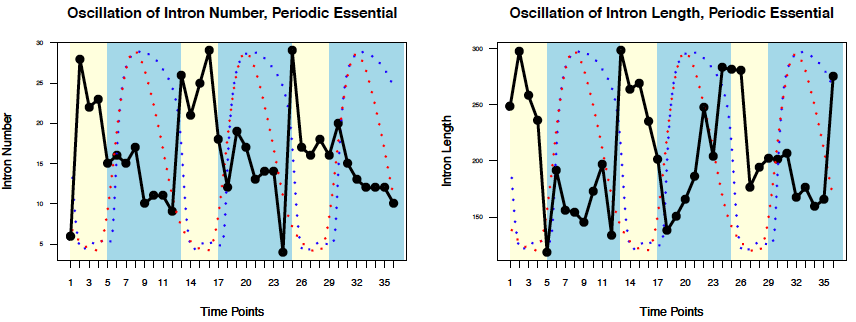


**Fig. S5A** The relationship between number and length of introns, 5’ and 3’UTRs in *S. cerevisiae* genes and the redox cycle. Yellow color (time points 1-4) is the oxidative phase, and the blue color (time points 5-12) is the reductive phase. Boxes show median values with statistical significance with notches (if two boxes' notches do not overlap this is a ‘strong evidence’ that their medians differ (2)), first quantile (25%) and third quantile (75%); whiskers indicate minimum and maximum values. **A.** The oscillation of intron number and length matching the period of the fluctuations in the oxidation state.

**
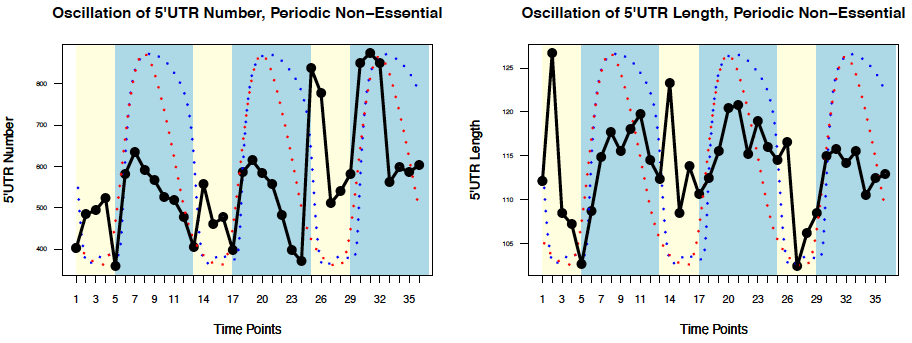
**

**
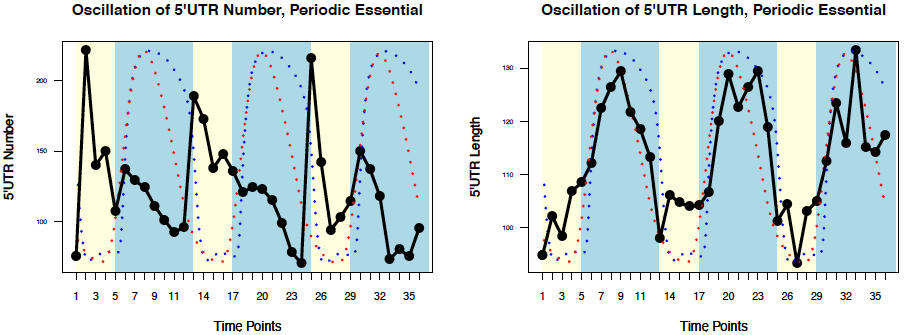
**

**Fig. S5B** The oscillation of 5’UTR number and length matching the period of the fluctuations in the oxidation state.


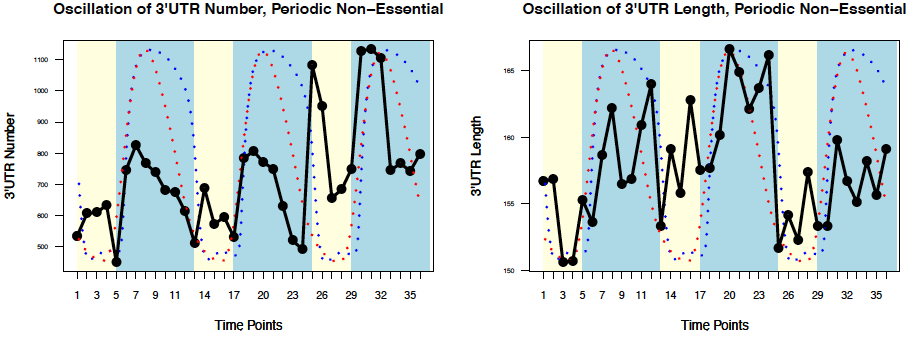


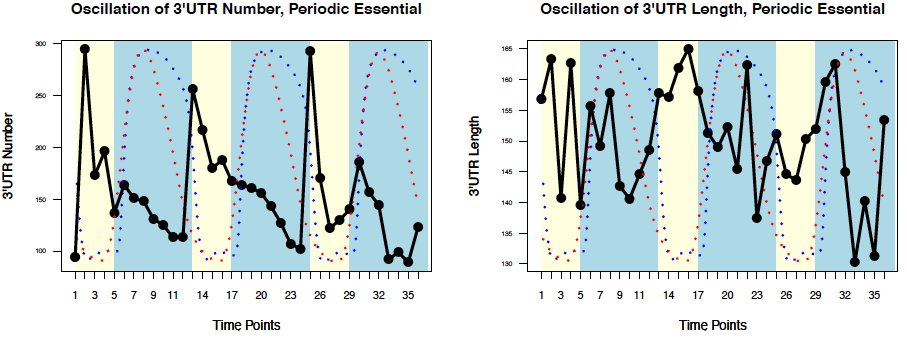


**Fig. S5C** The oscillation of 3’UTR number and length matching the period of the fluctuations in the oxidation state.


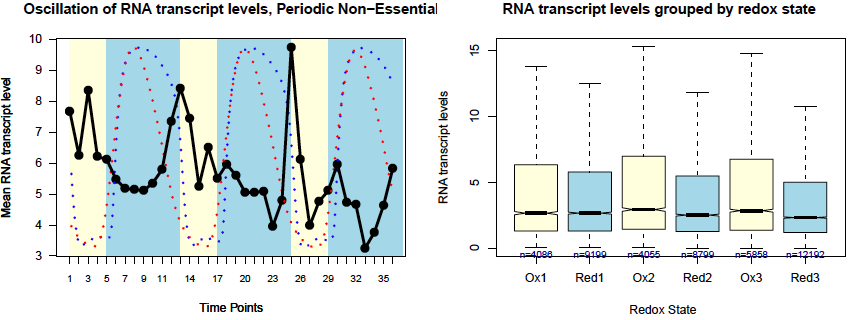


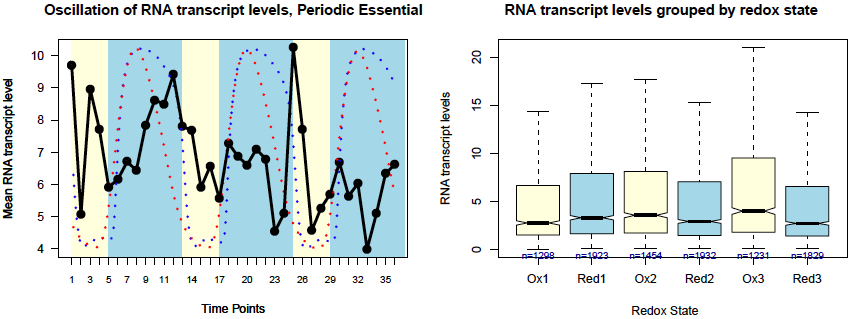


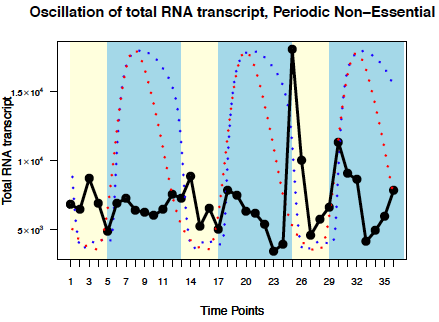

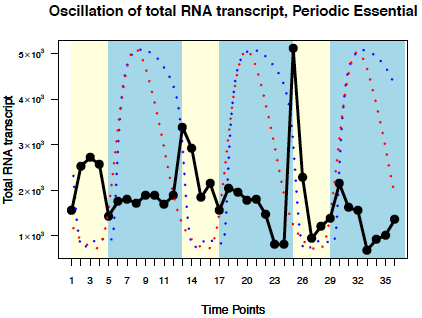


**Fig. S6** The relationship between mean RNA transcript levels, total RNA transcript levels of *S. cerevisiae* genes and the redox cycle. Yellow color (time points 1-4) is the oxidative phase, and the blue color (time points 5-12) is the reductive phase. Boxes show median values with statistical significance with notches (if two boxes' notches do not overlap this is a ‘strong evidence’ that their medians differ (2)), first quantile (25%) and third quantile (75%); whiskers indicate minimum and maximum values.

**
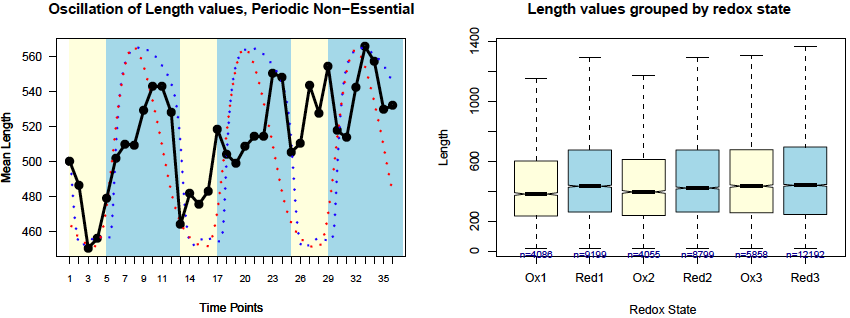
**

**
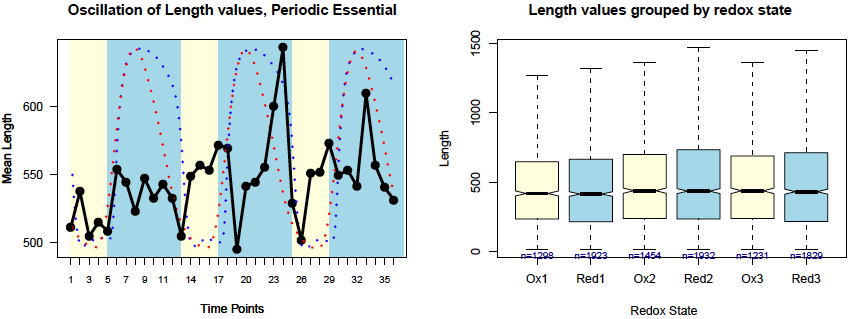
**

**Fig. S7** The relationship between ORF length and the redox cycle of *S. cerevisiae*. Yellow color (time points 1-4) is the oxidative phase, and the blue color (time points 5-12) is the reductive phase. Boxes show median values with statistical significance with notches (if two boxes' notches do not overlap this is a ‘strong evidence’ that their medians differ (2)), first quantile (25%) and third quantile (75%); whiskers indicate minimum and maximum values.


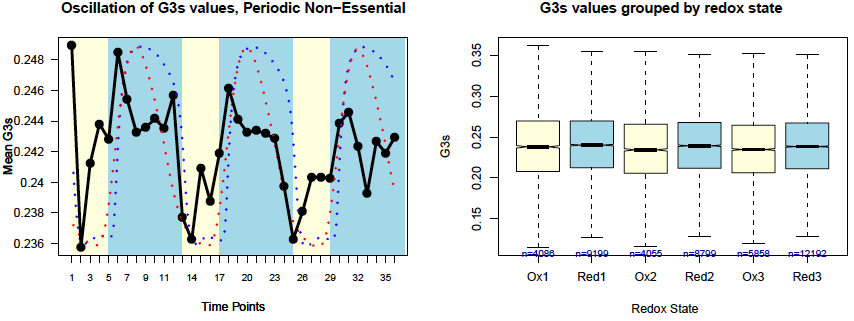


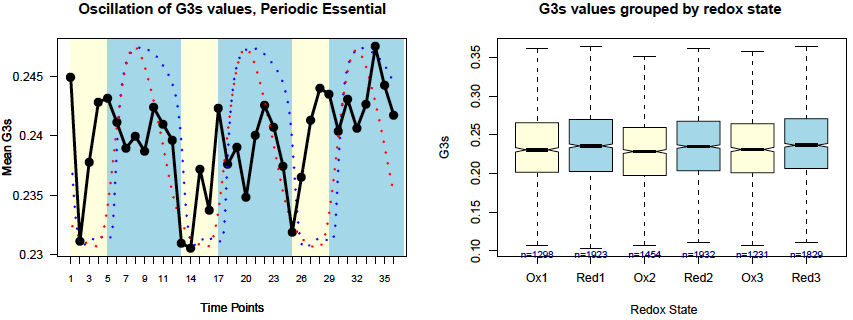


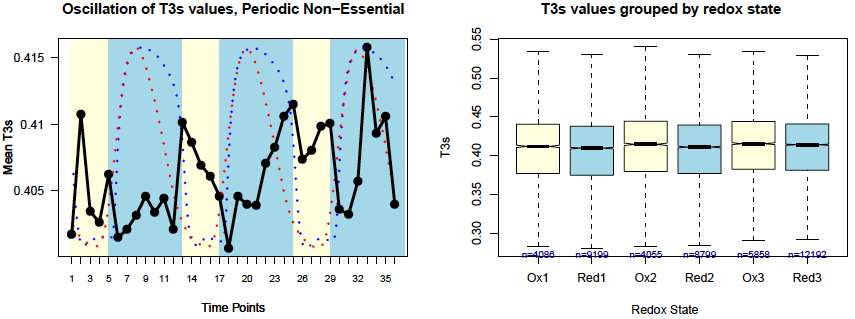

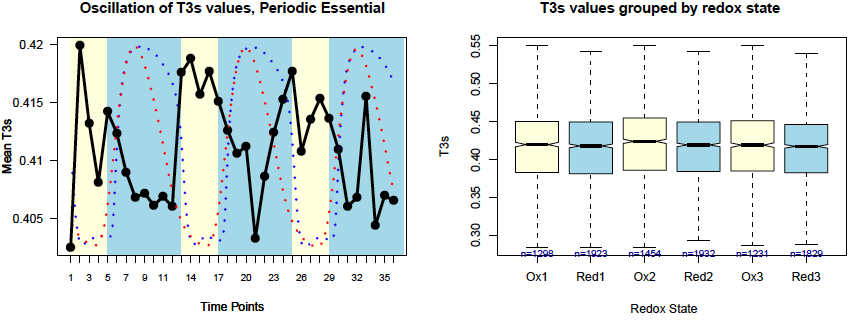


**
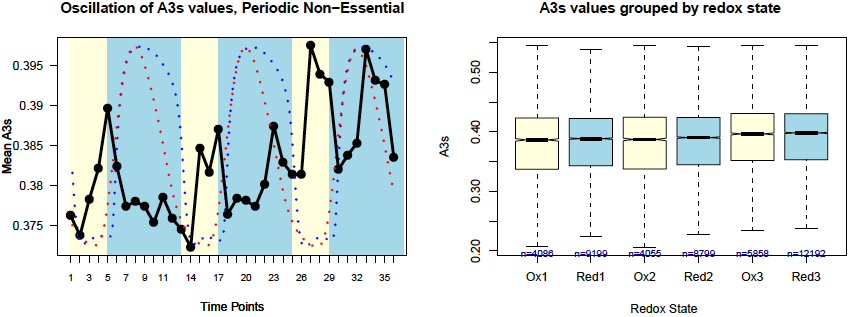
**

**
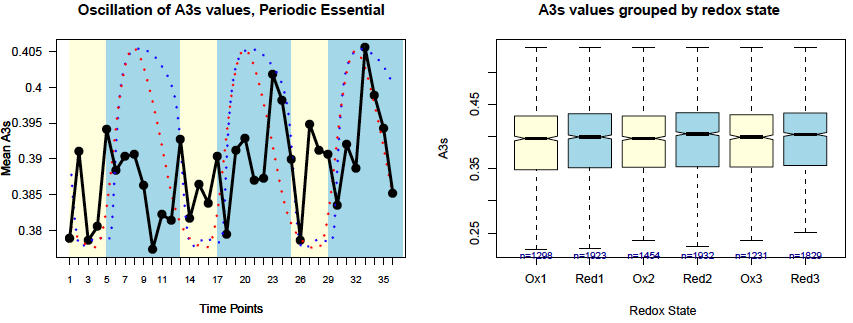
**

**
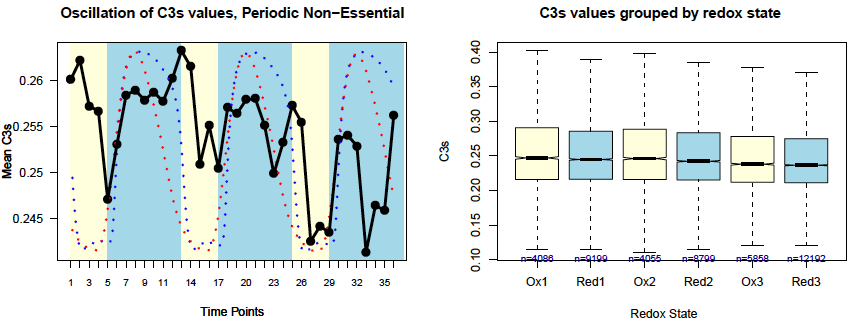
**

**
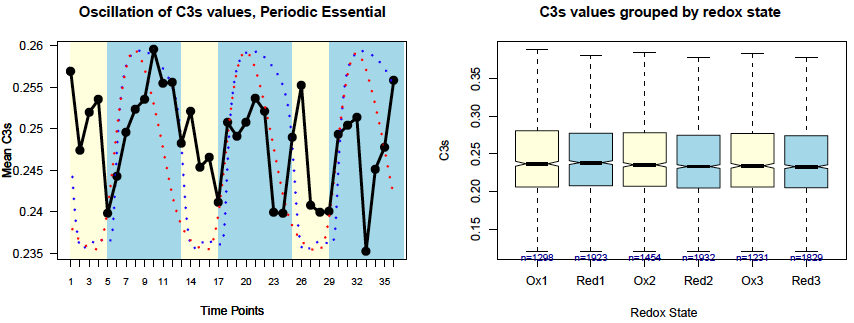
**

**Fig. S8** The relationship between mean occurrence of a specific nucleotide in the third base in synonymous codons of *S. cerevisiae* genes and the redox cycle of *S. cerevisiae*. Yellow color (time points 1-4) is the oxidative phase, and the blue color (time points 5-12) is the reductive phase. Boxes show median values with statistical significance with notches (if two boxes' notches do not overlap this is a ‘strong evidence’ that their medians differ (2)), first quantile (25%) and third quantile (75%); whiskers indicate minimum and maximum values.

**
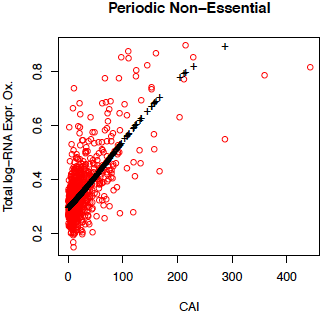

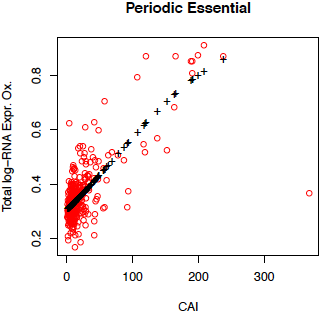
**

**Fig. S9** The relationship between CAI values and total log-normalized RNA expression level in the oxidative phase (time points 1-4) for each gene in *S. cerevisiae*. Red points represent the measured values, and the black points represent the best non-linear fit.

|  | Oxidative (1-4) | Reductive (5-12) |
| --- | --- | --- |
| CAI Non-Ess | 0.362±0.135(SD, N=4086)  0.369±0.140(SD, N=4055)  0.363±0.134(SD, N=5858) | 0.336±0.093(SD, N=9199)  0.337±0.099(SD, N=8799)  0.339±0.103(SD, N=12192) |
|  | p-value = 8.993e-13, p-value < 2.2e-16, p-value < 2.2e-16 | |
| CAI Ess | 0.386± 0.151(SD, N=1298)  0.395± 0.150(SD, N=1454)  0.387± 0.149(SD, N=1231) | 0.360± 0.121(SD, N=1923)  0.364± 0.130(SD, N=1932)  0.359± 0.126(SD, N=1829) |
|  | p-value = 9.467e-05, p-value = 9.16e-11, p-value = 7.326e-08 | |
| CAI 6 Non-Ess | 0.355± 0.120(SD, N=19839)  0.357± 0.121(SD, N=20341)  0.356± 0.121(SD, N=18630) | 0.347± 0.108(SD, N=44687)  0.348± 0.110(SD, N=43380)  0.348± 0.111(SD, N=41187) |
|  | p-value = 1.581e-06, p-value = 8.553e-13, p-value = 4.601e-10 | |
| CAI 6 Ess | 0.379± 0.142(SD, N=6592)  0.379± 0.140(SD, N=7340)  0.375± 0.140(SD, N=6330) | 0.366± 0.125(SD, N=9870)  0.367± 0.133(SD, N=10309)  0.366± 0.132(SD, N=9720) |
|  | p-value = 2.517e-05, p-value = 1.596e-10, p-value = 4.748e-05 | |
| CAI Hum Non-Ess | 0.380± 0.144(SD, N=3960)  0.386± 0.145(SD, N=4056)  0.388± 0.146(SD, N=3668) | 0.370± 0.142(SD, N=7127)  0.370± 0.141(SD, N=6851)  0.372± 0.140(SD, N=6986) |
|  | p-value = 0.0001643, p-value = 3.888e-09, p-value = 2.857e-07 | |
| CAI Hum Ess | 0.403± 0.151(SD, N=2109)  0.407± 0.149(SD, N=2150)  0.409± 0.153(SD, N=1954) | 0.375± 0.153(SD, N=3570)  0.375± 0.148(SD, N=3894)  0.379± 0.153(SD, N=3801) |
|  | p-value = 6.357e-13, p-value < 2.2e-16, p-value = 8.238e-14 | |
| Expr Non-Ess | 7.081± 12.334(SD, N=4086)  6.880± 10.354(SD, N=4055)  6.559± 9.938(SD, N=5858) | 5.624± 8.777(SD, N=9199)  5.190± 8.004(SD, N=8799)  4.805± 7.312(SD, N=12192) |
|  | p-value = 0.04752, p-value = 3.553e-15, p-value < 2.2e-16 | |
| Expr Ess | 7.223± 12.153(SD, N=1298)  7.083± 9.213(SD, N=1454)  7.754± 9.876(SD, N=1231) | 7.313± 10.969(SD, N=1923)  6.355± 9.003(SD, N=1932)  5.846± 8.231(SD, N=1829) |
|  | p-value = 0.003922, p-value = 7.017e-06, p-value = 4.986e-12 | |

**Table S1.** Table of mean CAI values for *S. cerevisiae*, *Saccharomyces sensu stricto* and *H. sapiens*, mean log-normalized RNA expressions, standard deviations, sample gene number, and two-tailed Wilcoxon *p*-values for oxidative and reductive phases of *S. cerevisiae* cycle.

|  | Oxidative (1-4) | Reductive (5-12) |
| --- | --- | --- |
| Sim 6 Non-Ess | 79.317± 15.457(SD, N=30969)  78.780± 15.556(SD, N=32321)  78.435± 15.580(SD, N=28723) | 77.012± 15.717(SD, N=70862)  77.063± 15.835(SD, N=67239)  77.170± 15.711(SD, N=66147) |
|  | p-value < 2.2e-16, p-value < 2.2e-16, p-value < 2.2e-16 | |
| Sim 6 Ess | 80.222± 16.389(SD, N=8181)  81.222± 16.079(SD, N=8110)  80.577± 15.970(SD, N=6968) | 80.027± 16.189(SD, N=14965)  80.215± 16.177(SD, N=16051)  79.752± 16.389(SD, N=15088) |
|  | p-value = 0.4141, p-value = 2.078e-05, p-value = 0.01838 | |
| Sim Hum Non-Ess | 63.681± 13.257(SD, N=5118)  63.074± 12.907(SD, N=5114)  62.173± 12.961(SD, N=4717) | 62.125± 12.094(SD, N=11156)  62.017± 12.128(SD, N=9864)  61.633± 12.008(SD, N=10623) |
|  | p-value = 5.221e-08, p-value = 0.0002054, p-value = 0.4764 | |
| Sim Hum Ess | 64.210± 12.530(SD, N=2736)  63.651± 11.612(SD, N=2686)  62.726± 11.322(SD, N=2342) | 64.629± 12.161(SD, N=4630)  63.790± 11.484(SD, N=4781)  63.460± 10.933(SD, N=4636) |
|  | p-value = 0.05138, p-value = 0.3582, p-value = 0.001529 | |

**Table S2.** Table of mean pair wise phylogenetic sequence similarity values between *S. cerevisiae* and *Saccharomyces sensu stricto*, and between *S. cerevisiae* and *H. sapiens*, standard deviations, sample gene number, and two-tailed Wilcoxon *p*-values for oxidative and reductive phases of *S. cerevisiae* cycle.

| Frequency of sequence variation | Oxidative (1-4) | | Reductive (5-12) |
| --- | --- | --- | --- |
| ORFs Non-Ess | 0.00155± 0.00104(SD, N=4086)  0.00153± 0.00102(SD, N=4055)  0.00153± 0.00102(SD, N=5857) | | 0.00165± 0.00104(SD, N=9199)  0.00162± 0.00102(SD, N=8799)  0.00161± 0.00103(SD, N=12184) |
|  | p-value = 2.464e-09, p-value = 2.205e-06, p-value = 1.020e-06 | | |
| ORFs Ess | 0.00140± 0.00101(SD, N=1296)  0.00140± 0.00100(SD, N=1452)  0.00140± 0.00100(SD, N=1230) | | 0.00148± 0.00102(SD, N=1915)  0.00150± 0.00103(SD, N=1925)  0.00146± 0.00101(SD, N=1821) |
|  | p-value = 0.009672, p-value = 0.003585, p-value = 0.07197 | | |
| Introns Non-Ess | 0.00251± 0.00234(SD, N=210)  0.00252± 0.00227(SD, N=212)  0.00284± 0.00283(SD, N=274) | | 0.00157± 0.00202(SD, N=219)  0.00177± 0.00212(SD, N=233)  0.00212± 0.00261(SD, N=377) |
|  | p-value = 4.869e-07, p-value = 3.776e-06, p-value = 4.305e-06 | | |
| Introns Ess | 0.00331± 0.00335(SD, N=79)  0.00315± 0.00302(SD, N=101)  0.00355± 0.00362(SD, N=80) | 0.00270± 0.00343(SD, N=104)  0.00226± 0.00260(SD, N=111)  0.00288± 0.00322(SD, N=110) | |
|  | p-value = 0.02609, p-value = 0.003695, p-value = 0.07791 | | |
| 5’-UTRs Non-Ess | 0.00470± 0.00619(SD, N=1903)  0.00486± 0.00657(SD, N=1898)  0.00504± 0.00785(SD, N=2664) | 0.00426± 0.00545(SD, N=4253)  0.00422± 0.00559(SD, N=3988)  0.00451± 0.00685(SD, N=5503) | |
|  | p-value = 0.02955, p-value = 0.001531, p-value = 0.03248 | | |
| 5’-UTRs Ess | 0.00531± 0.00867(SD, N=587)  0.00500± 0.00756(SD, N=648)  0.00572± 0.01013(SD, N=555) | 0.00428± 0.00486(SD, N=897)  0.00434± 0.00615(SD, N=900)  0.00445± 0.00509(SD, N=842) | |
|  | p-value = 0.543, p-value = 0.911, p-value = 0.9117 | | |
| 3’-UTRs Non-Ess | 0.00286± 0.00339(SD, N=2386)  0.00282± 0.00317(SD, N=2366)  0.00275± 0.00303(SD, N=2067) | 0.00304± 0.00359(SD, N=5500)  0.00298± 0.00354(SD, N=5285)  0.00307± 0.00357(SD, N=5016) | |
|  | p-value = 0.09204, p-value = 0.1748, p-value = 0.009627 | | |
| 3’-UTRs Ess | 0.00342± 0.00635(SD, N=758)  0.00325± 0.00523(SD, N=841)  0.00324± 0.00410(SD, N=715) | 0.00376± 0.00743(SD, N=1082)  0.00376± 0.00734(SD, N=1127)  0.00369± 0.00643(SD, N=1030) | |
|  | p-value = 0.05439, p-value = 0.01068, p-value = 0.07224 | | |

**Table S3.** Table of mean frequencies of sequence variation per nucleotide over the time of divergence that resulted in the 63 strains (in ORFs, introns, 5’ and 3’UTRs), standard deviations, sample gene number, and two-tailed Wilcoxon *p*-values for the oxidative and reductive phases of *S. cerevisiae* redox cycle.

**References**

1. Lloyd, D. and Murray, D.B. (2006) The temporal architecture of eukaryotic growth. FEBS Lett 580: 2830-2835.

2. Chambers, J., Cleveland, W., Kleiner, B. and Tukey, P., (1983) *Graphical Methods for Data Analysis*, Wadsworth. P. 63.

3. Tu, B.P., Kudlicki, A., Rowicka, M., McKnight, S.L. (2005) Logic of the yeast metabolic cycle: temporal compartmentalization of cellular processes. Science 310: 1152-1158.

4. Klevecz, R.R., Bolen, J., Forrest, G., Murray, D.B. (2004) A genomewide oscillation in transcription gates DNA replication and cell cycle. Proc Natl Acad Sci USA 101: 200-205.

5. Akaike, Hirotugu, (1974) A new look at the statistical model identification. IEEE Transactions on Automatic Control 19: 716–723.

6. Wichert, S., Fokianos, K., and K. Strimmer (2004) "Identifying Periodically Expressed Transcripts in Microarray Time Series Data", Bioinformatics 20: 5-20.

7. Cooley, J. W. and Tukey, J.W. (1965) An Algorithm for the Machine Computation of the Complex Fourier Series," Mathematics of Computation*,* 19: 297-301.

8. Peden, J. F., *CodonW*. (1999) PhD Thesis, University of Nottingham.

9. SGD project. "Saccharomyces Genome Database" <http://downloads.yeastgenome.org/> 01/2010 release. Accessed December 1, 2010

10. Nagalakshmi, U., Wang, Z., Waern, K., Shou, C., Raha, D., Gerstein, M., and Snyder, M., (2008) The Transcriptional Landscape of the Yeast Genome Defined by RNA Sequencing. Science 320: 1344-1349.

11. Samanta, M.P., Tongprasit, W., Sethi, H., Chin, C.S., Stolc, V., (2006) Global identification of noncoding RNAs in Saccharomyces cerevisiae by modulating an essential RNA processing pathway. Proc Natl Acad Sci U S A 103: 4192-4197.

12. Doniger, S.W., Kim, H.S., Swain, D., Corcuera, D., Williams, M., Yang, S.P., Fay, J.C., (2008) A catalog of neutral and deleterious polymorphism in yeast. PLoS Genet 4: e1000183.

13. Schacherer, J., Shapiro, J.A., Ruderfer, D.M., Kruglyak, L. (2009) Comprehensive polymorphism survey elucidates population structure of Saccharomyces cerevisiae. Nature 458: 342-345.

14. Efron, Bradley (1981) Censored data and the bootstrap, *Journal of the American Statistical Association* 76: 312-319.

15. Schwarz, Gideon E., (1978) Estimating the dimension of a model. *Annals of Statistics* 6: 461–464.

16. Ikemura, T., (1981) Correlation between the abundance of *Escherichia coli* transfer RNAs and the occurrence of the respective codons in its protein genes: a proposal for a synonymous codon choice that is optimal for the *E. coli* system. Journal of Molecular Biology 151: 389-409.

17. Sharp, P. M., and W. H. Li, (1987) The codon adaptation index a measure of directional synonymous codon usage bias, and its potential applications. Nucleic Acids Research 15: 1281-1295.

18. Wright, F., (1990) The effective number of codons used in a gene. Gene 87: 23-29.
